# A diverse Late Cretaceous vertebrate tracksite from the Winton Formation of Queensland, Australia

**DOI:** 10.7717/peerj.11544

**Published:** 2021-06-17

**Authors:** Stephen F. Poropat, Matt A. White, Tim Ziegler, Adele H. Pentland, Samantha L. Rigby, Ruairidh J. Duncan, Trish Sloan, David A. Elliott

**Affiliations:** 1 Australian Age of Dinosaurs Museum of Natural History, Winton, Queensland, Australia; 2 Faculty of Science, Engineering and Technology, Swinburne University of Technology, Hawthorn, Victoria, Australia; 3 School of Environmental & Rural Science, University of New England, Armidale, Armidale, New South Wales, Australia; 4 Palaeontology, Museums Victoria, Melbourne, Victoria, Australia

**Keywords:** Dinosauria, Sauropoda, Theropoda, Ornithopoda, Crocodyliformes, Testudines, Dipnoi, Actinopterygii, Swim tracks, Ichnology

## Abstract

The Upper Cretaceous ‘upper’ Winton Formation of Queensland, Australia is world famous for hosting Dinosaur Stampede National Monument at Lark Quarry Conservation Park, a somewhat controversial tracksite that preserves thousands of tridactyl dinosaur tracks attributed to ornithopods and theropods. Herein, we describe the Snake Creek Tracksite, a new vertebrate ichnoassemblage from the ‘upper’ Winton Formation, originally situated on Karoola Station but now relocated to the Australian Age of Dinosaurs Museum of Natural History. This site preserves the first sauropod tracks reported from eastern Australia, a small number of theropod and ornithopod tracks, the first fossilised crocodyliform and ?turtle tracks reported from Australia, and possible lungfish and actinopterygian feeding traces. The sauropod trackways are wide-gauge, with manus tracks bearing an ungual impression on digit I, and anteriorly tapered pes tracks with straight or concave forward posterior margins. These tracks support the hypothesis that at least one sauropod taxon from the ‘upper’ Winton Formation retained a pollex claw (previously hypothesised for *Diamantinasaurus matildae* based on body fossils). Many of the crocodyliform trackways indicate underwater walking. The Snake Creek Tracksite reconciles the sauropod-, crocodyliform-, turtle-, and lungfish-dominated body fossil record of the ‘upper’ Winton Formation with its heretofore ornithopod- and theropod-dominated ichnofossil record.

## Introduction

Australia’s dinosaur fossil record, for much of the Mesozoic Era, would be a ghastly blank (or close enough) were it not for ichnofossils. The only evidence for the presence of Triassic dinosaurs in Australia comprises theropod tracks from Queensland (Hill, Playford & Woods, 1965; Staines & Woods, 1964; Thulborn, 2000; Thulborn, 2003). The Australian Jurassic dinosaur record also largely constitutes tracks—all from Queensland—evincing the presence of theropods, ornithopods, and thyreophorans (Anonymous, 1951; Anonymous, 1952a; Anonymous, 1952b; Ball, 1933; Ball, 1934a; Ball, 1934b; Ball, 1946; Bartholomai, 1966; Bryan & Jones, 1946; Colliver, 1956; Cook, Saini & Hocknull, 2010; Haubold, 1971; Hill, Playford & Woods, 1966; Romilio, 2020a; Romilio, 2020b; Romilio et al., 2020; Romilio, Salisbury & Jannel, 2020; Staines, 1954; Thulborn, 1994; Thulborn, 1997; Turner et al., 2009). These supplement the sole Jurassic dinosaur body fossil from Queensland, the holotype specimen of the sauropod *Rhoetosaurus brownei* (Jannel et al., 2019; Longman, 1926; Longman, 1927a; Longman, 1927b; Nair & Salisbury, 2012; Todd et al., 2019). The geologically oldest Cretaceous dinosaur-bearing unit in Australia, the Valanginian–Barremian Broome Sandstone of Western Australia, preserves abundant dinosaur tracks representing a diverse fauna of sauropods, theropods, ornithopods, and thyreophorans (Colbert & Merrilees, 1967; Glauert, 1952; Romilio et al., 2017; Salisbury et al., 2017; Thulborn, 2012; Thulborn, Hamley & Foulkes, 1994), but has yielded no body fossils to date.

In Australia, it is only in the Barremian–Cenomanian interval that the information derived from the dinosaur body fossil record exceeds that derived from ichnofossils. Although the upper Barremian–lower Aptian upper Strzelecki Group (‘Wonthaggi Formation’) and the upper Aptian–lower Albian Eumeralla Formation in Victoria have produced both avian and non-avian dinosaur tracks (Martin, 2016; Martin et al., 2012; Martin et al., 2014) and possible dinosaur burrows (Martin, 2009), dinosaur body fossils in each unit are far more abundant, and are representative of a far greater faunal diversity, than ichnofossils (see Poropat et al. (2018) and references therein). The upper Albian marine units of the Eromanga Basin—the Toolebuc Formation, Allaru Mudstone, and Mackunda Formation—have (unsurprisingly) not produced dinosaur tracks, despite yielding body fossils (see Pentland & Poropat (2019) and references therein; and Poropat et al. (2017) and references therein). Lastly, whereas the dinosaur body fossil record of the Cenomanian Griman Creek Formation of New South Wales reflects a relatively high diversity fauna (see Bell et al. (2019) and references therein), very few dinosaur tracks have been reported (Molnar, 1991; Smith & Smith, 1999; Thulborn, 1990; Thulborn, 1994), and none have been formally described.

The Cenomanian–?lowermost Turonian ‘upper’ Winton Formation is the only Cretaceous unit in Queensland to have produced dinosaur tracks. Indeed, it hosts the most famous dinosaur tracksite in Australia: Dinosaur Stampede National Monument at Lark Quarry Conservation Park. This site, which preserves several thousand tridactyl tracks, has been the subject of much research since the 1970s (Romilio & Salisbury, 2011; Romilio & Salisbury, 2014; Romilio, Tucker & Salisbury, 2013; Thulborn, 2013; Thulborn, 2017; Thulborn & Wade, 1979; Thulborn & Wade, 1984; Thulborn & Wade, 1989; White, Cook & Rumbold, 2017), and has been widely popularised in a variety of media (Hocknull, 2013; Knowles, 1980; Meiklejohn, 2004; Poropat, 2017; Wade, 1979a; Wade, 1979b). And yet, to date, Lark Quarry (including the adjacent and laterally equivalent Seymour Quarry and New Quarry) is the only tracksite that has been formally reported from the Winton Formation. The only other possible dinosaur track from the Winton Formation that has been alluded to in the literature (QM F52282; Romilio, Tucker & Salisbury, 2013) is one of several undescribed tridactyl tracks derived from a single site on Elderslie Station (McKenzie, 2004).

In this article, we describe a new vertebrate ichnoassemblage from the Winton Formation: The Snake Creek Tracksite. This tracksite preserves the first sauropod tracks reported from the Winton Formation—and, indeed, from eastern Australia—as well as numerous tridactyl tracks made by small-bodied bipedal dinosaurs (theropods and ornithopods). Two clear sauropod trackways, one of which can be followed for more than 40 m, are preserved at the Snake Creek Tracksite, along with three less clear-cut trackways and several other less distinct sauropod tracks. Most of the tridactyl tracks are small, but the one exception—an isolated tridactyl penetrative track—might represent a medium-sized theropod. Of the small tridactyl tracks, a few are nearly identical to those of *Skartopus australis* from Lark Quarry (Thulborn & Wade, 1984), whereas others are indistinguishable from those of *Wintonopus latomorum* from the same site (Thulborn & Wade, 1984); herein, we maintain these as separate ichnotaxa, following Thulborn (2017).

In addition, the Snake Creek Tracksite preserves several trackways made by underwater walking crocodyliforms and ?turtles, as well as probable fish feeding traces; all of these reports are firsts for Australia in rocks of any age. Five trackways can be assigned to the crocodyliform ichnogenus *Hatcherichnus*, whereas other tracks appear to have been made by freshwater turtles. The several probable feeding traces identified along the western margin of the Tracksite are tentatively ascribed to lungfish and actinopterygian fish.

The Snake Creek Tracksite diminishes the apparent disparity between the ichnofossil and body fossil records of the Winton Formation by preserving ichnites of ornithopods and theropods, which have until now dominated the ichnofossil record, alongside those of sauropods and crocodyliforms, and possibly turtles and lungfish, all of which dominate the body fossil record.

## Geological setting

The Eromanga Basin is one of several interconnected basins that collectively comprise the Great Australian Superbasin (Cook, Bryan & Draper, 2013). The Euroka Arch separates the Eromanga Basin from the Carpentaria Basin to the northwest, whereas the Nebine Ridge forms the barrier between it and the Surat Basin to the southeast (Exon & Senior, 1976). Across its vast extent, the basement of the Eromanga Basin is heterogeneous, with the Galilee Basin and Maneroo Platform underlying the Winton region (Fergusson & Henderson, 2013). Although localised deposits within the Eromanga Basin are of Late Triassic age (Gray, McKillop & McKellar, 2002), broad scale sedimentary infill did not commence until the Early Jurassic (Cheng et al., 2020). Whereas fluviatile and lacustrine deposits characterise the Jurassic–earliest Cretaceous of the Basin (Senior, Mond & Harrison, 1978), much of the Early Cretaceous sequence comprises paralic to marine units that were deposited at the bottom of the epeiric Eromanga Sea (Day, 1969; Cook, 2012). The cessation of deposition of the Mackunda Formation and onset of Winton Formation deposition approximately corresponds with the final regression of the Eromanga Sea (Day, 1969; Cook, 2012; Cook, Bryan & Draper, 2013).

**Figure 1:**
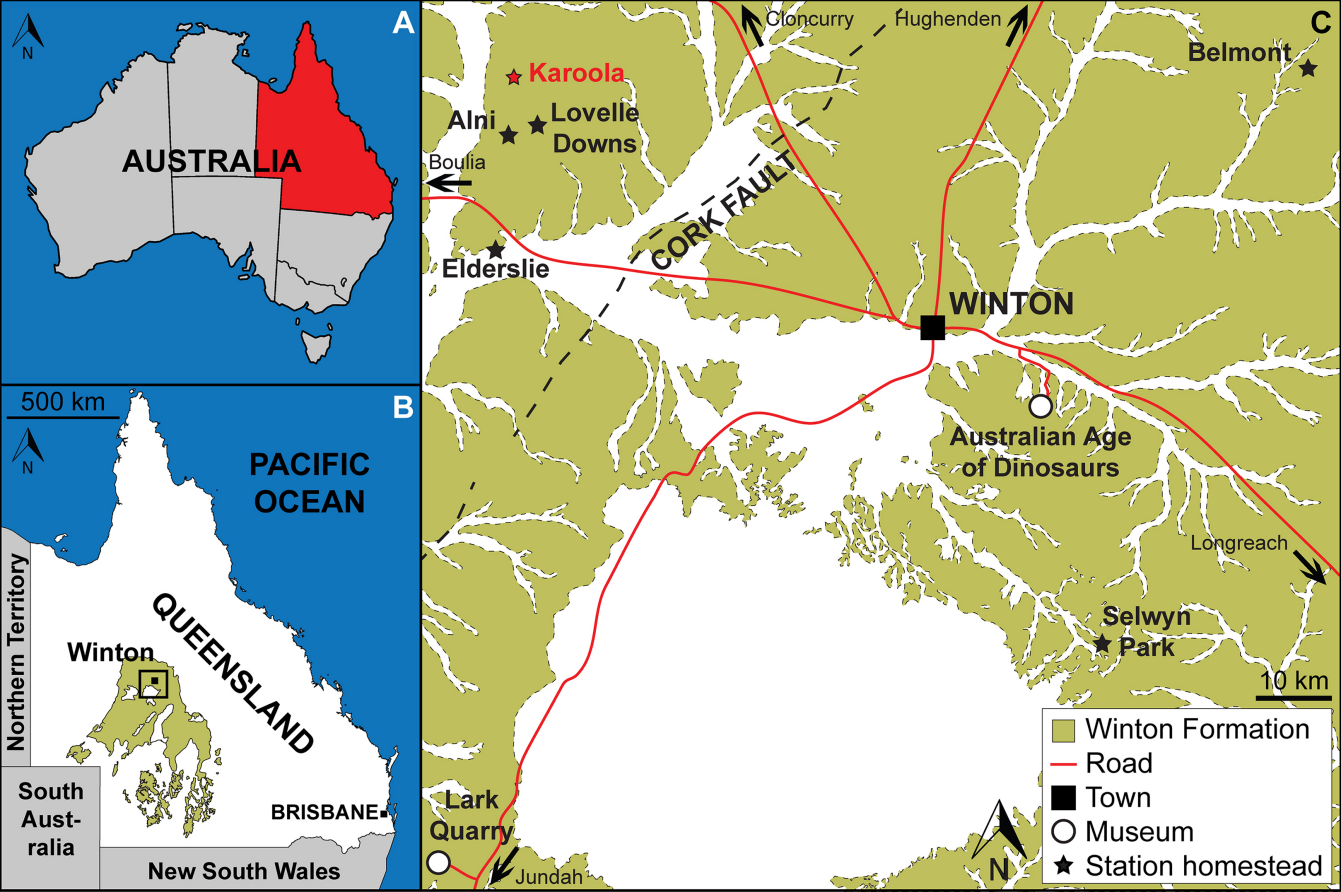
Location of the Snake Creek Tracksite. (A) Map of Australia showing the location of Queensland (modified from Poropat et al. (2016)). (B) Map of Queensland plotting the distribution of Winton Formation outcrop (modified from Poropat et al. (2016)). (C) Map of the Winton area illustrating the Winton Formation outcrop, the location of Karoola Station, and numerous other cattle/sheep stations and sites in the region from which dinosaur remains have been exhumed and/or in which they are on display. Map was drafted by the senior author (S.F.P.) in Adobe Illustrator CC 2017 (modified from Pentland et al. (2019)), incorporating geological information from Vine (1964) and Vine & Casey (1967) (© Commonwealth of Australia (Geoscience Australia) 2021. This product is released under the Creative Commons Attribution 4.0 International Licence. http://creativecommons.org/licenses/by/4.0/legalcode).

The Winton Formation (Fig. 1) is the geologically youngest Mesozoic unit in the Eromanga Basin (Vine & Day, 1965). It has a maximum thickness of over 1,000 m, and is situated at or just below the surface across much of its geographic range (Senior, Mond & Harrison, 1978). Despite this, it crops out only sporadically (Whitehouse, 1954), owing to deep weathering during the Cainozoic (Senior & Mabbutt, 1979). The ‘upper’ Winton Formation, within which the Snake Creek Tracksite is preserved, dominantly comprises blue-grey volcanogenic mudstones, siltstones, and sandstones, along with rare conglomerates (Exon & Senior, 1976; Senior, Mond & Harrison, 1978; Tucker et al., 2017). These sediments were deposited in a generally low-relief floodplain environment, characterised by a temperate climate with high rainfall (Fletcher, Moss & Salisbury, 2018).

**Figure 2:**
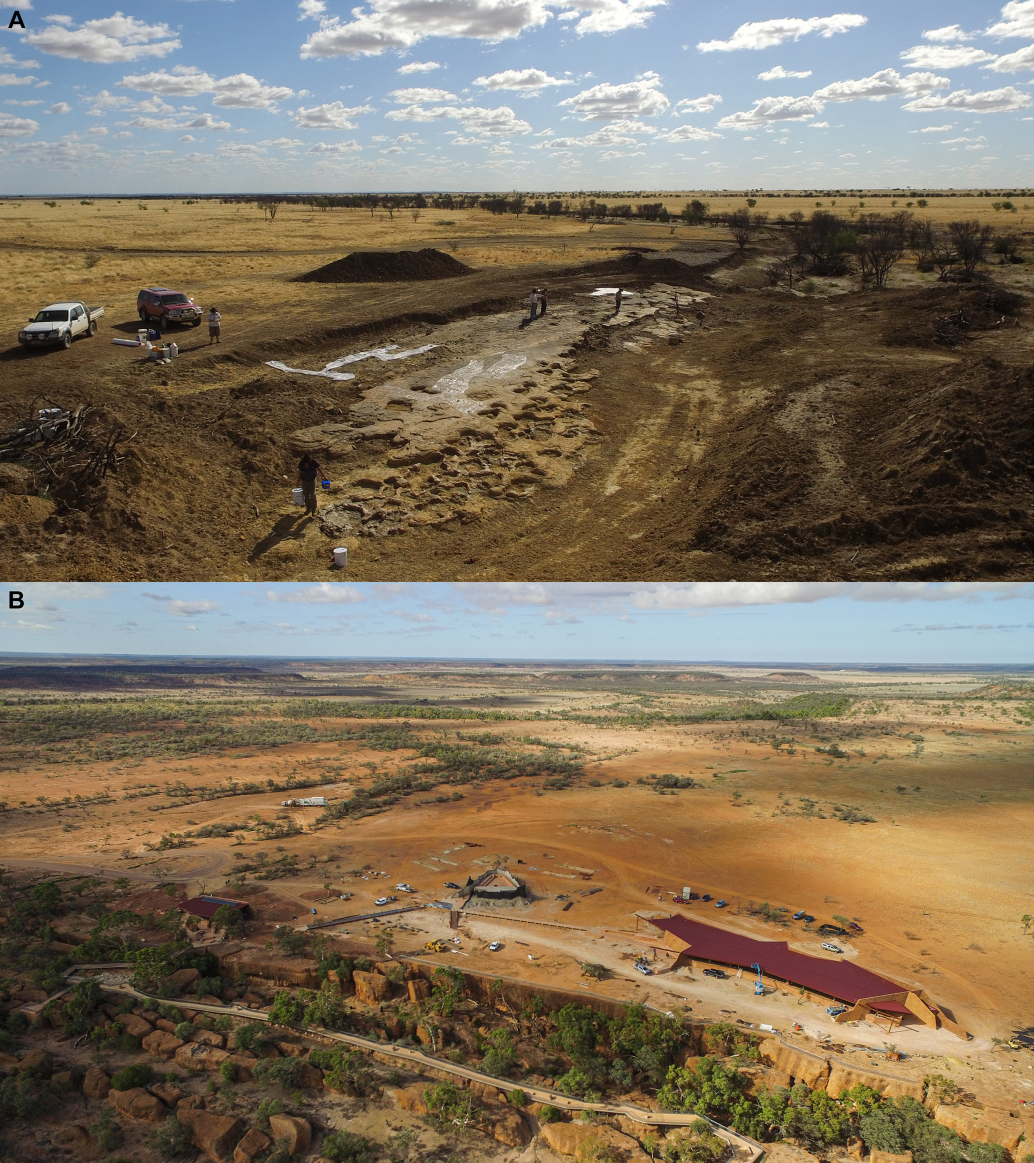
Snake Creek Tracksite in situ and relocated. (A) Drone photograph of the Snake Creek Tracksite in situ, taken on 29 May 2018 from the southeast. Although one section of the site preserving small dinosaur tracks was covered in latex when this photo was taken, the sauropod tracks can clearly be seen. (B) Drone photograph of Stages 3.1 and 3.2 of the *Australian Age of Dinosaurs Museum of Natural History*. The structures depicted are *Dinosaur Canyon* (left and foreground), the *Gondwana Stars Observatory* (centre), and the *March of the Titanosaurs* building (right), wherein the Snake Creek Tracksite now resides.

**Figure 3:**
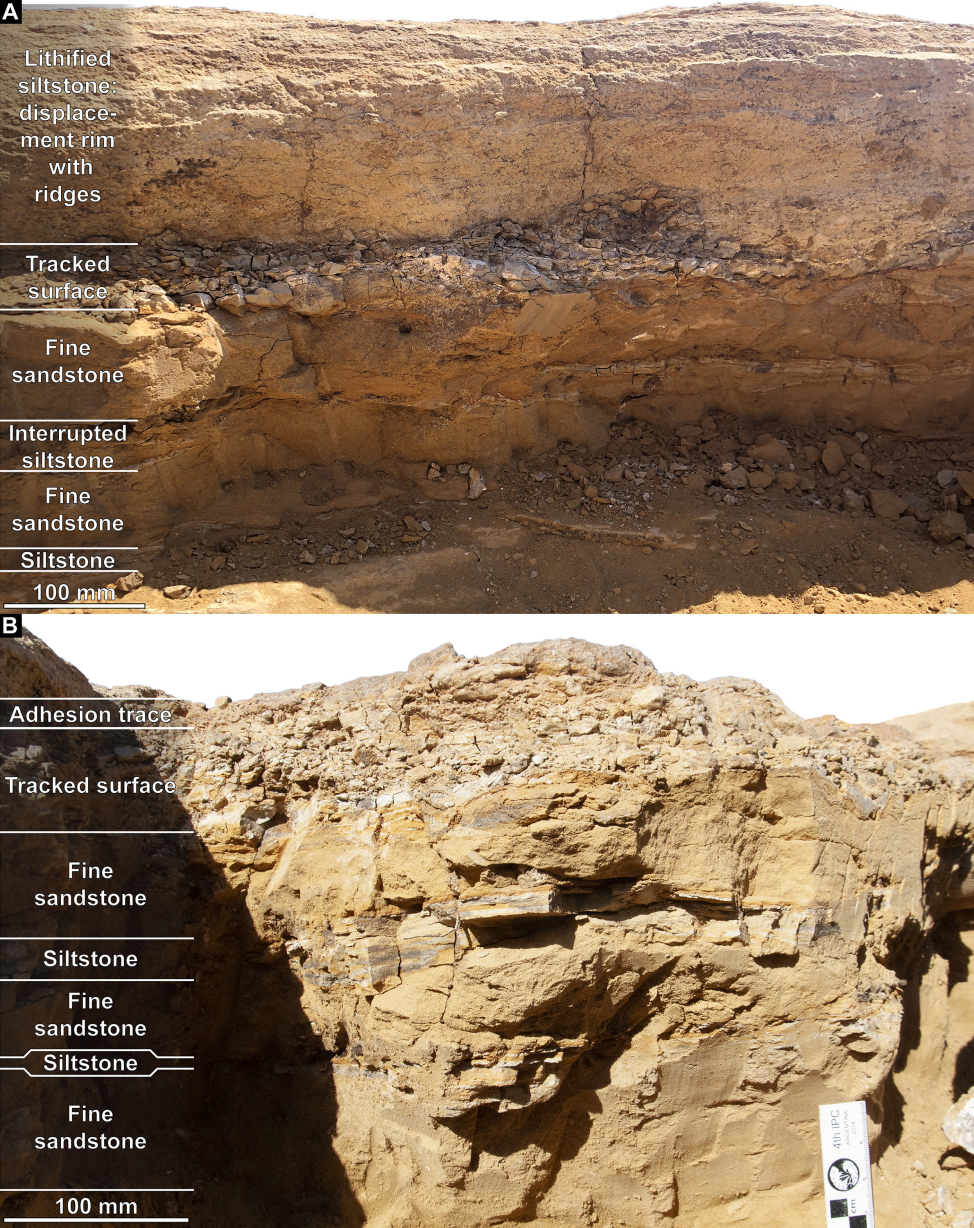
Snake Creek Tracksite stratigraphic sections. (A) Photo taken within sauropod right pes track AODF 904.S1.08, showing the lateral (northeast-facing) wall, depicting a ~350 mm thick sedimentary sequence. (B) Photo of the 350 mm thick sedimentary sequence that immediately underlies, and includes, the flattened sediment pad immediately posterior to the sauropod right pes track AODF 904.S1.10 (northeast-facing surface).

Prior to its excavation, the Snake Creek Tracksite was situated on Karoola Station, northwest of Winton, Queensland (Figs. 1C, 2). Specifically, the tracksite was located in the bed of Snake Creek, approximately 4.5 km east of the property’s homestead (GPS locality data is available from the Australian Age of Dinosaurs Museum of Natural History on request). The tracks at the Snake Creek Tracksite are preserved in a tan–grey siltstone layer that is approximately 20 mm thick (Fig. 3A); however, when the median adhesion traces preserved within some sauropod tracks are included as part of the tracked surface, it is ~70 mm thick (Fig. 3B). Immediately beneath the tracked surface, the succession comprises three thick sandstone layers interspersed with two thin siltstone layers (Fig. 3). The intermittent sandstone and siltstone layers are laterally continuous for tens of meters beneath the tracked surface.

**Figure 4:**
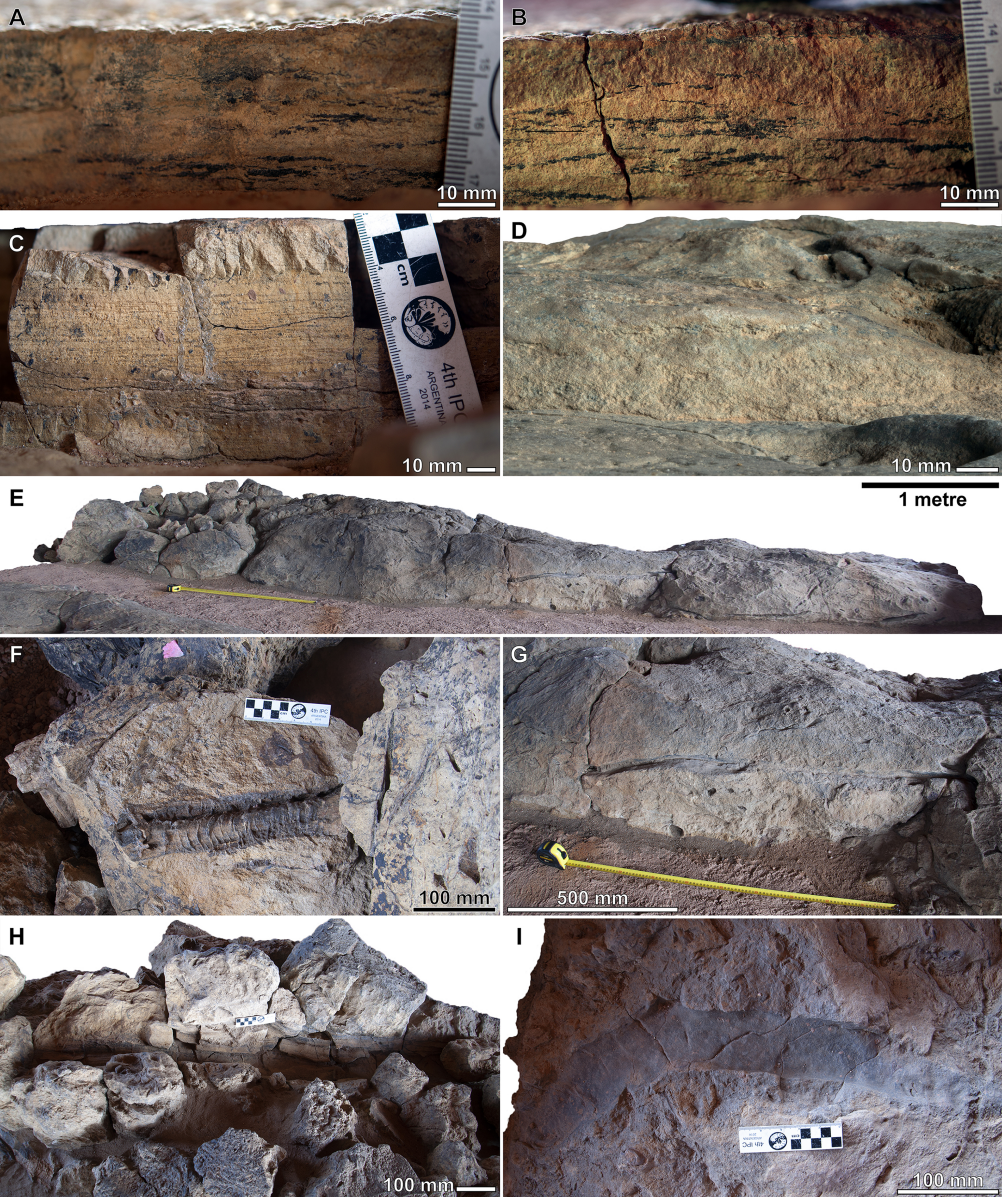
Snake Creek Tracksite stratigraphic sections. (A) Photo of a 30 mm thick sedimentary sequence, with the tracked surface at the top, lateral to the anterior margin of the sauropod manus track AODF 904 S2.13 and exposed on its southwest face. (B) Photo of a 40 mm thick sedimentary sequence, with the tracked surface at the top, lateral to the anterior margin of the sauropod pes track AODF 904 S2.11 and exposed on its southwest face. (C) Photo of an 80 mm thick sedimentary sequence, slightly below (and not including) the tracked surface, anterolateral to the anterior margin of the sauropod manus track AODF 904 S2.13 and exposed on its southwest face. (D) Photo of a 40 mm thick sedimentary sequence, immediately above (and showing) the tracked surface, lateral and anterolateral to a probable turtle track (pictured) in the southeast section of the Snake Creek Tracksite and exposed to the east. (E) Siltstone levee present at the northern end of the site, the long axis of which runs west–east, preserving sporadic plant fossils and possible burrows. (F) Presumed conifer branch at the western end of the bar. (G) Tree branch aligned with its long axis parallel to that of the levee, situated at the mid-length of the section transported to the Museum. (H) Tree branch aligned with its long axis perpendicular to that of the levee, situated in the western third of the section transported to the Museum. (I) Dorsally exposed putative burrow at the eastern end of the section of the levee transported to the Museum.

Along the western margin of the Tracksite, the 2 mm thick uppermost layer of the tracked surface is sepia-coloured in section and often has a pustular surface texture, potentially evincing the formation of microbial mats at the site (Figs. 4A–4B). Beneath this, the tracked horizon comprises siltstones that possess multiple sepia-coloured laminations when freshly exposed (Fig. 4C), although these siltstones lose their lamination when weathered. The tracked surface is exposed across much of the Tracksite; however, large sections in the west of the Tracksite have been weathered such that lower levels are exposed, and small sections in the southeast are overlain by a massive, occasionally gypsum-rich, fine sandstone (Fig. 4D). The tracked surface has an eastward dip of 5°.

North–northwest of the end of the Tracksite, a massive, well-sorted siltstone deposit is present, with its long axis striking west–east (Fig. 4E). This siltstone is interpreted as a levee, deposited on the bank of a meandering river channel. Notably, this levee contains several conifer(?) branches that are well-separated from one another by intervening matrix (Fig. 4F). These branches show no preferred orientation: some have their long axes aligned parallel with that of the levee (Fig. 4G), whereas others are aligned perpendicular (Fig. 4H). There is some suggestion that the siltstone levee was laterally burrowed into (Fig. 4I), although the incomplete preservation of the putative burrow (i.e. the absence of its upper half/two-thirds, assuming it was approximately cylindrical) precludes rigorous assessment of the possible burrower(s).

**Figure 5:**
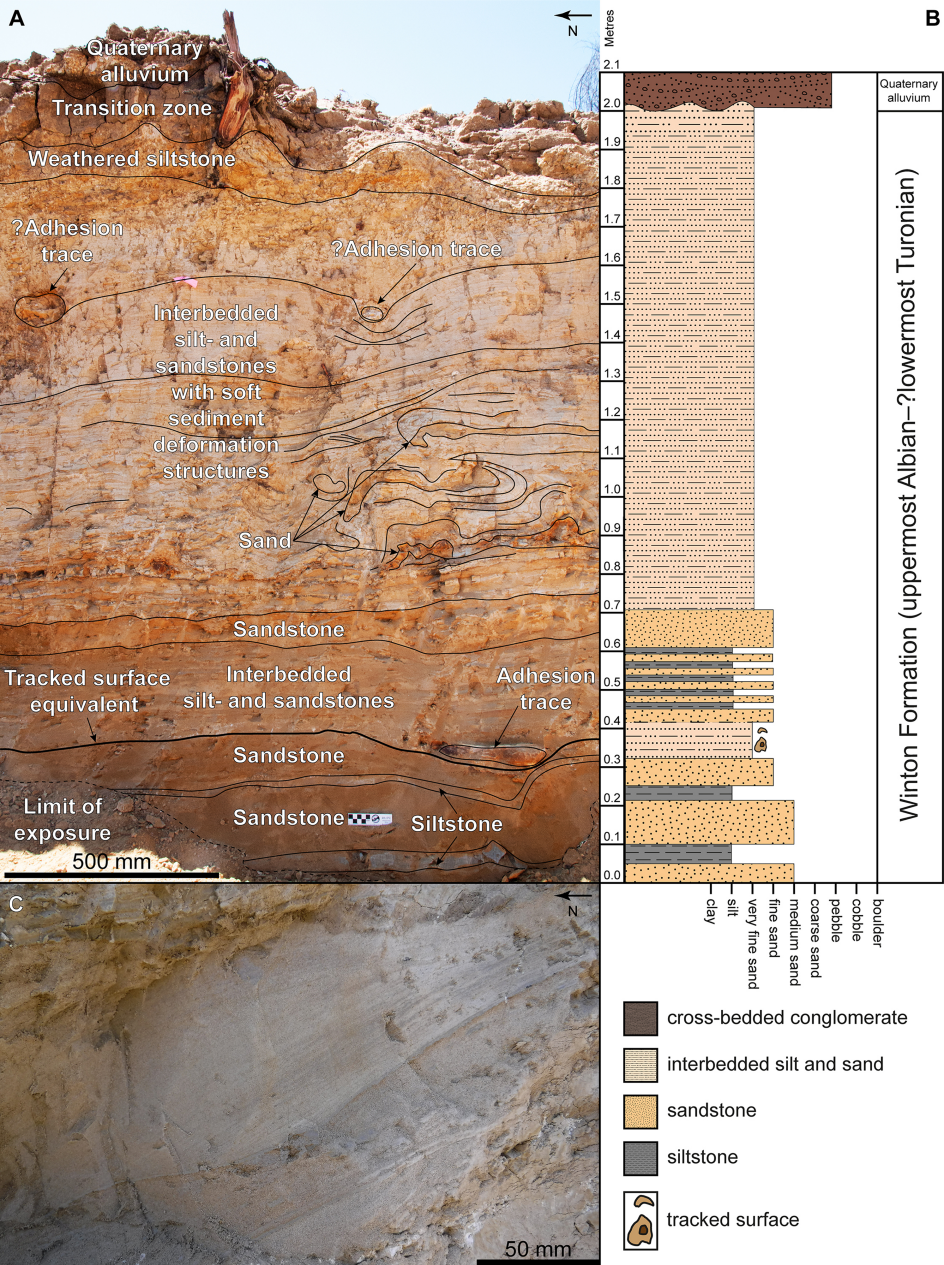
Snake Creek Tracksite adjacent stratigraphic section. (A) Sedimentary sequence, situated less than 20 m east of the southeast end of the Snake Creek Tracksite, exposed using earth-moving equipment in mid-September 2018. The tracked surface was correlated with this section based on its dip angle (5°N). The presence of an apparent adhesion trace exposed in cross section supported this correlation, as did the observation of the same alternating sedimentary layers (thick sandstone–thin siltstone–thick sandstone–thin siltstone) situated directly beneath both the track layer in this section and the lithified tracks nearest to it. (B) Stratigraphic column of the same section (and to the same scale), showing alternation between silt- and sandstones throughout. (C) Cross-section through an apparent sauropod track, infilled with sediment, with cross-lamination indicative of a northward palaeocurrent flow.

Extrapolation of the dip angle of the tracked surface of the Snake Creek Tracksite enabled the identification of laterally equivalent, non-cemented strata near the base of a sedimentary succession approximately 20 m east of the southeast end of the Tracksite (Fig. 5). The presence of several concretions at this level, similar to those found within sauropod tracks on the tracked surface, and the presence of a well-preserved sequence of alternating thick siltstones and thin sandstones below the tracked surface in both successions, supports this correlation. The tracked surface in the adjacent section is underlain by alternating thick siltstone and thin sandstone layers (Figs. 5A–5B), as is the case underneath the Tracksite itself (Fig. 3). One cross-laminated sandstone lens—interpreted as a cross-section through an infilled sauropod track (Fig. 5C)—indicates a south–north palaeoflow in the layer immediately above the tracked surface. The tracked surface in this sedimentary section is superimposed by a sequence of fine-grained sedimentary rocks similar to those found overlying the tracked surface on the Tracksite; however no sign of plant debris was evident, implying that the levee present at the northern end of the site was restricted in lateral extent, and possibly in place prior to the traverse of any of the trackmakers. Above the aforementioned fine-grained sedimentary rocks, the sedimentary succession adjacent to the Tracksite comprises interbedded coarse siltstones and very fine sandstones (grain size varying from 0.5–1.0 mm). Convolute lamination of the interbedded strata has produced discontinuous siltstone layers, with sandstones frequently affected by soft sediment deformation caused by subsequent “dinoturbation” events; higher in the sequence, these were identified based on localised bed compression, irregularly associated with sediment infilling or lithified adhesion traces. Although a transitional, montmorillonite-rich vertisol layer (colloquially termed “black soil”) is present towards the top of the adjacent section, and originally blanketed much of the Tracksite proper, the uppermost layer in the adjacent sedimentary sequence comprises Quaternary gravels and coarse sands deposited by more recent floodwaters in Snake Creek.

The sedimentary succession at the Snake Creek Tracksite is indicative of overbank deposits adjacent to a meandering river, probably near an abandoned channel that became an oxbow lake (Cant, 1982; Boggs, 2001; Reineck & Singh, 1980). Vertical accretion appears to have been the dominant mode of sediment deposition, since few ripples or other indicators of lateral flow are present below or within the tracked surface, or in overlying or underlying strata. A freshwater palaeoenvironment is inferred on the basis of both prior work on the Winton Formation (see Fletcher, Moss & Salisbury (2018) and references therein), the lack of marine fossils, and on the presence of probable lungfish feeding traces (owing to the fact that post-Palaeozoic lungfish are predominantly non-marine (Schultze, 2004; Clack, Sharp & Long, 2011)).

## Methods

The Snake Creek Tracksite was cleared of vegetation and overburden using a front-end loader, a small excavator and a 40 mm high pressure pneumatic hose. Brooms and hand tools were used to remove smaller quantities of sediment. Broken sections of the northern margin of the tracked surface (including the lateral borders of three left manus-pes sets from the main sauropod trackway) were restored where possible using araldite and superglue. Fragile sections of the Tracksite were consolidated using solutions of 10–25% w/v Paraloid B72 in acetone.

Following the guidelines outlined by Falkingham et al. (2018), the Snake Creek Tracksite was photographed in situ for photogrammetry on 13/04/2018 using a Canon EOS 6D camera with the following settings: ISO 200; F-stop of f/4; and exposure time 1/4,000 seconds. In all, 3,005 photographs were taken, and these were converted into a 3D model using Agisoft PhotoScan/Metashape (www.agisoft.com) by Christopher “Kit” Nelson (Abergower Digital). In addition, numerous aerial shots of the Snake Creek Tracksite were taken with a DJI FC300XW camera mounted on a DJI Phantom 3 4K Professional Drone (www.dji.com/au/phantom3-4k). Several sections of the site were also surface scanned with an Artec Space Spider (www.artec3d.com/portable-3d-scanners/artec-spider-v2), and the resultant three-dimensional models (and the photogrammetric model of the entire site) were scaled, manipulated and (in some cases) aligned in Artec Studio 15 Professional (www.artec3d.com/3d-software/artec-studio). The horizontal plane of each surface was estimated in this software package (attempts to do so in CloudCompare (www.cloudcompare.org) proved futile), and colour depth maps were generated in ParaView (www.paraview.org). Images were assembled in Adobe Photoshop 2021 and outlined and annotated in Adobe Illustrator 2021.

The entire Snake Creek Tracksite is registered as AODF 904. Specific trackways on the site have been numbered based on the clade by which they are interpreted to have been made: S, sauropod; T, theropod; O, ornithopod; and C, crocodyliform. Tracks within each trackway are numbered from least to most recently formed, with left tracks invariably assigned odd numbers and right tracks assigned even numbers. In cases where gaps are interpreted to exist in a trackway, the numbering sequence is adjusted to account for the missing tracks. In the most questionable trackways, tracks are assigned letters rather than numbers.

**Figure 6:**
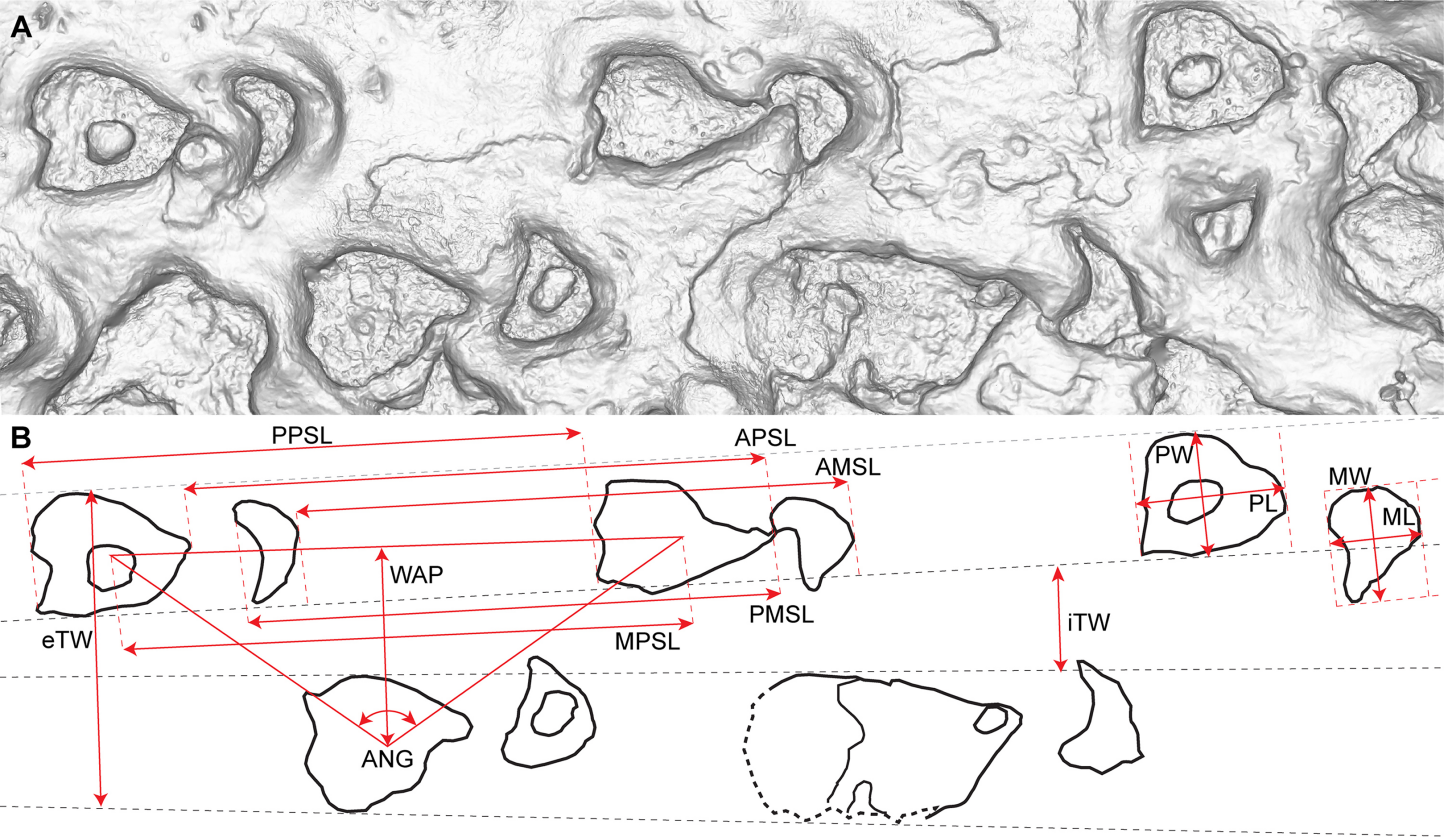
Key to measurements used in this paper. (A) Orthographic projection of the monochromatic photogrammetric rendering of the Snake Creek Tracksite, zoomed to a section of the AODF 904.S1 sauropod trackway (AODF 904.S1.07–11). (B) Schematic derived from the image below, showing track outlines and the following track and trackway measurements: AMSL, anterior manus stride length; APSL, anterior pes stride length; ANG, pace angulation; eTW, external trackway width; iTW, internal trackway width; ML, manus track length; MPSL, middle pes stride length; MW, manus track width; PL, pes track length; PMSL, posterior manus stride length; PPSL, posterior pes stride length; PW, pes track width; WAP, width of the pedal track angulation pattern.

Descriptive terminology used in this paper follows Marty, Falkingham & Richter (2016). Track dimensions and stride and pace lengths were measured directly on the track surface using a retractable tape measure. The thicknesses of the beds both on the tracksite itself and in the adjacent sedimentary section were also measured with a retractable tape measure. Maximum track length was measured anteroposteriorly on each track, and maximum track width was measured perpendicular to this measurement. Four measurements of stride length were obtained from the main sauropod trackway: the distance between the anterior tips of two successive manus tracks; the distance between the posterior tips of two successive manus tracks; the distance between the anterior tips of two successive pes tracks; and the distance between the posterior tips of two successive pes tracks (Fig. 6). Pace and stride length in non-sauropodan tracks were each measured from the middle of the posterior margin of one track to the same position on the next. The degree of heteropody in the sauropod trackways was determined following the methodology outlined by Salisbury et al. (2017)

Between April 2018 and November 2020, the entire Snake Creek Tracksite was relocated to the Australian Age of Dinosaurs Museum of Natural History, southeast of Winton, Queensland. This work was carried out by systematically disassembling the tracksite along existing fractures and loading each section onto a three-tonne trailer for transport to the Museum. Each trailer-load of Tracksite was repositioned in its new location at the Museum before the next load was dismantled and collected. Excavation, relocation, and preservation of the trackway was led by Judy Elliott and David Elliott. The excavation and removal of the Snake Creek Tracksite—the lateral dimensions of which are ~54 m × ~15 m, and the mass of which is estimated to be ~300 tonnes—might be the largest scale dinosaur trackway relocation project ever completed; the only comparable undertaking of which we are aware is the 1940 relocation of several sections of a sauropod and theropod trackway from the Paluxy River, Texas, and in this case the section removed was 9 m × 3.65 m (Bird, 1941; Bird, 1954; Bird, 1985; Falkingham, Bates & Farlow, 2014; Farlow & Lockley, 1989; Lockley & Hunt, 1995).

Prior to the discovery of the Snake Creek Tracksite, the tracked surface and the layers immediately underlying it had suffered from substantial weathering and periodic inundation by floodwaters. Trees had grown within or alongside several tracks, and the northeast margin of the site (especially near the sauropod tracks AODF 904.S1.13–20) had been heavily impacted by erosion, such that the medial margins of several left sauropod manus–pes couplets had deteriorated. However, this otherwise detrimental process had two fortuitous consequences that were exploited during excavation. First, the extensive fracturing of the Tracksite meant that few blocks with surface areas exceeding one square metre remained intact. Second, the sedimentary layers beneath the Tracksite had weathered to the point that they comprised soft, unconsolidated sandstone, meaning that the Tracksite was effectively sitting on loose sediment. The combination of these factors meant that very few new breaks needed to be made to facilitate dismantling of the entire Tracksite for transportation. The track walls in the southeast section of the site were undercut and encased in field jackets to ensure safe retrieval.

Relocation methods varied according to block size and fragility. Exceptionally large, robust blocks were loaded by tractor or front-end loader on to the bed of a three-tonne trailer, where sandbags were positioned to cushion and spread their weight. More manageable blocks were rolled onto wooden pallets, whereas smaller pieces were numbered or named according to their position in the site and packed in large plastic trays or plastic bags for transportation. To ease reassembly, counterpart joining surfaces of all portions of the site were marked with paint pens as they were pulled apart, and mobile phone cameras were used to record their positions as an added precaution. Drone photographs of the site taken prior to removal of the Tracksite were referred to throughout the repositioning and restoration process.

The Snake Creek Tracksite has been positioned at its new site in the same orientation (i.e., with its long axis projecting northwest–southeast) and with the same dip angle (~5° E) as that in which it was found. This was achieved by obtaining survey height readings and triangulation measurements that correlated to a centreline marked along the full length of the Tracksite prior to removal. Surveying was undertaken by Hoffmann Surveyors, who also documented the outer margins and orientation of the Tracksite. This enabled a compacted gravel pad to be built at the Museum that reflected the exact shape and orientation of the original site. This survey data was also used to design an 885 m^2^ site-specific building to house the trackway. Named *March of the Titanosaurs*, the building project was funded by the Queensland Government and construction of the new facility commenced in November 2019, running parallel with the Tracksite relocation until November 2020. The temperature-controlled facility, which opened to the public on 8 May 2021, will ensure that the Snake Creek Tracksite is protected from degradation by weathering, vegetation growth, channel flow, and agriculture, and made accessible to tourists and researchers, in perpetuity.

## Distribution and preservation of the tracks

**Figure 7:**
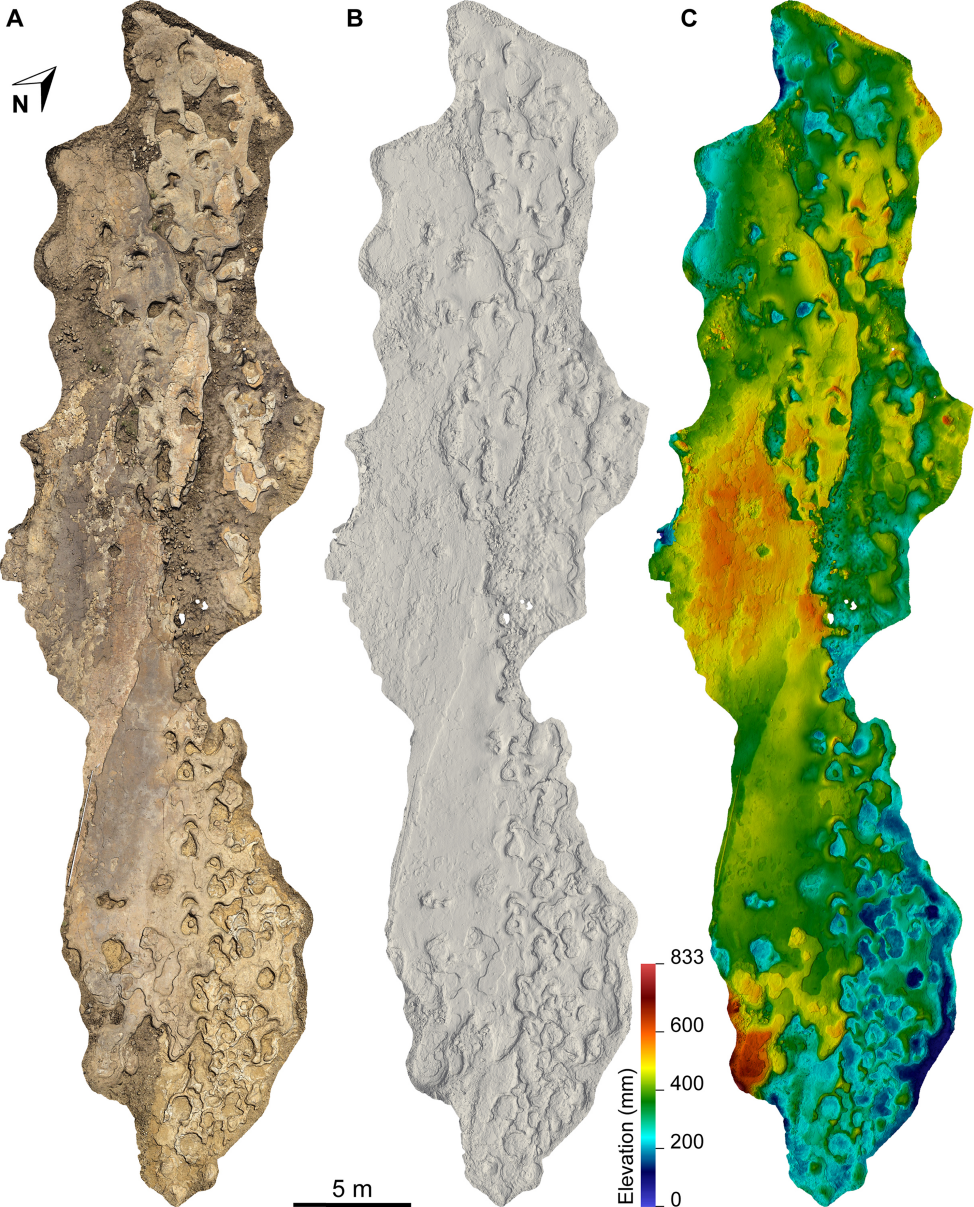
Maps of the Snake Creek Tracksite. All images in this figure are orthographic projections of the photogrammetric rendering of the Snake Creek Tracksite in situ, derived from photographs taken on 13 April 2018. The white gaps are ‘holes’ in the 3D surface, which correspond to the positions of modern tree stumps that were removed from the model. (A) Aerial view with colour texture overlay (derived from photograph data). (B) Aerial view in monochrome, illuminated from the southwest. (C) Aerial view colour depth map, with the highest topographic points in red and the lowest in violet.

**Figure 8:**
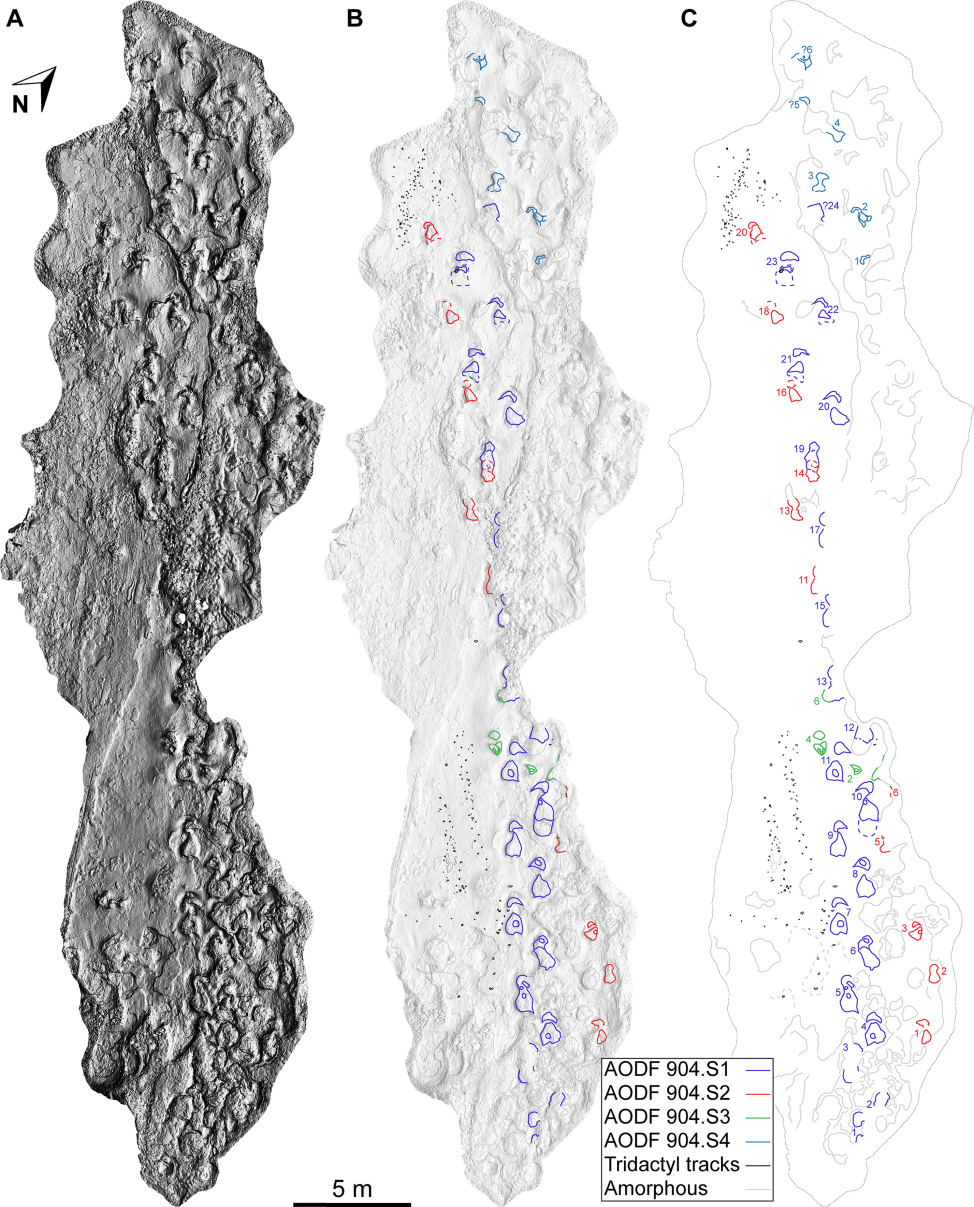
Maps of the Snake Creek Tracksite. All images in this figure are orthographic projections of the photogrammetric rendering of the Snake Creek Tracksite in situ, derived from photographs taken on 13 April 2018. The small white gaps are ‘holes’ in the 3D surface, which correspond to the positions of modern tree stumps that were edited out of the model. (A) Aerial view in monochrome, illuminated from the southwest, equalised in Photoshop to emphasise topography. (B) Aerial view in monochrome, illuminated from the southeast, with tracks and amorphous structures outlined. (C) Aerial view outline with tracks and amorphous structures outlined and annotated.

**Figure 9:**
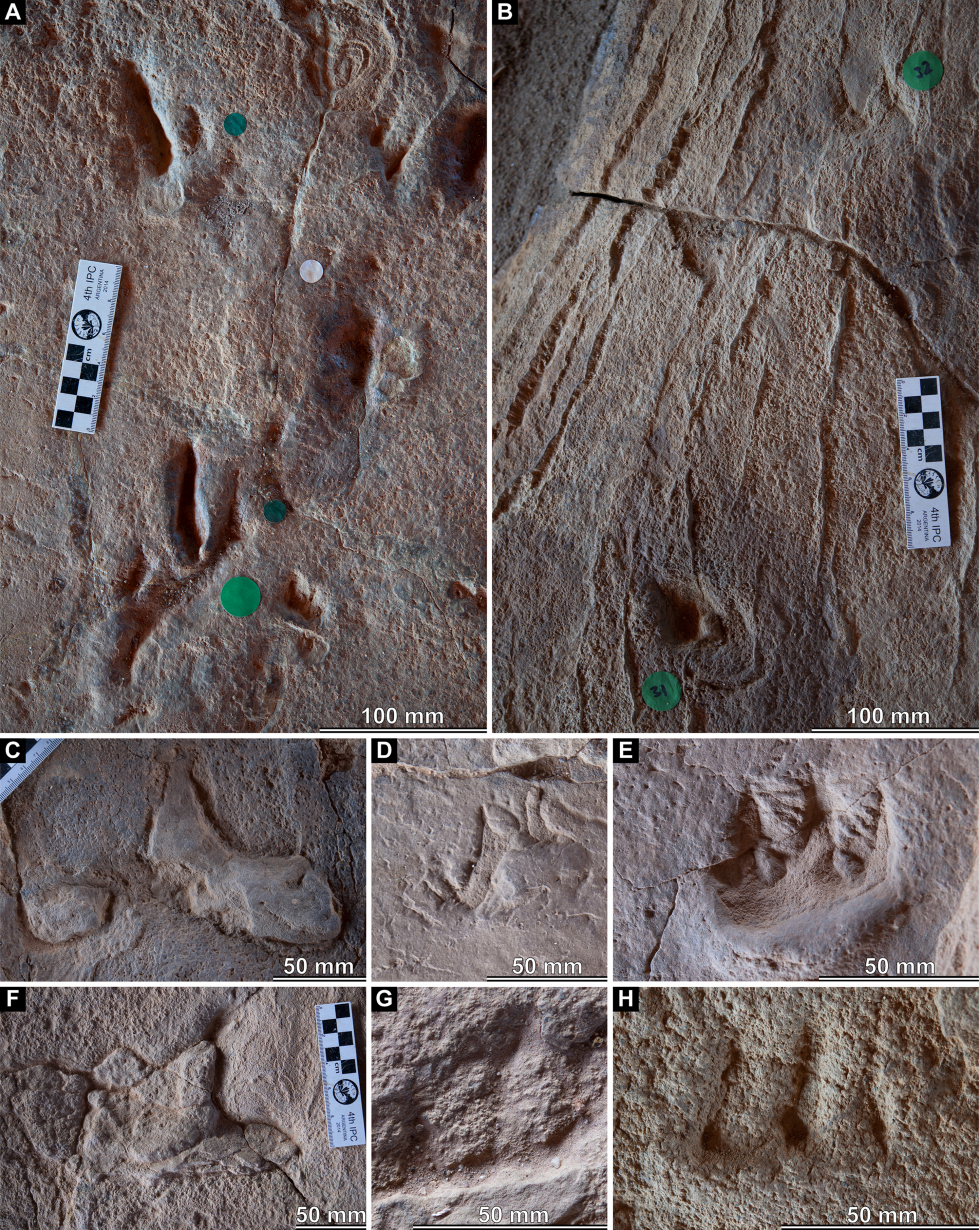
Track preservation at the Snake Creek Tracksite. (A) Two possibly associated, seemingly tridactyl and mesaxonic tracks, preserved as concave epireliefs, alongside a possible horsetail stem in cross section (circular structure, top right) and a tiny crocodyliform left pes track (to the right of the large green circle). (B) Two tracks, preserved as concave epireliefs, made by a crocodyliform walking underwater (*Hatcherichnus*: AODF 904.C1.31–32), that have disrupted the ridges on the displacement rim of the sauropod left pes track AODF 904.S1.07, implying that the crocodyliform traversed the site after the sauropod. (C) ?*Wintonopus* pes track, preserved as an infilled concave epirelief. (D) ?Turtle track, preserved as an infilled concave epirelief. (E) ?Turtle track, preserved as a partially infilled concave epirelief. (F) ?*Wintonopus* pes track, preserved as an infilled concave epirelief. (G) ?Turtle track, preserved as a transmitted track through overlying fine sandstone. (H) ?Turtle track, preserved as a transmitted track through overlying fine sandstone.

The Snake Creek Tracksite is approximately 54 m long (northwest–southeast) and 15 m wide (northeast–southwest) (Figs. 7–8). All tracks are preserved as moulds (concave epireliefs; Figs. 9A–9B), with a minority of tridactyl tracks preserving their corresponding casts (convex hyporeliefs; Figs. 9C–9F) and others evidently exposed as transmitted tracks in the overlying, coarser-grained fine sandstone (Figs. 9G–9H). Most of the sauropod tracks preserve track walls, displacement rims, and—irregularly—median adhesion traces sensu Carey et al. (2011). Unlike the track walls and the tracked surface, the sedimentary layer that hosted the floors of most of the sauropod true tracks is not lithified. Nevertheless, it was possible to identify the true tracked surface in section as a sandstone–siltstone contact, level with the bases of the adhesion traces (Fig. 3A). The tridactyl theropod, ornithopod, and crocodyliform tracks, and the tetradactyl ?turtle tracks, are invariably true tracks and are substantially shallower than the sauropod tracks.

Belvedere & Farlow (2016) introduced a numerical scale to enable the quantification of preservation quality in vertebrate tracks, and this was further developed by Marchetti et al. (2019). Following this scale, which runs from preservation grade 0.0 (the least-well preserved tracks) to 3.0 (the best-preserved), many of the sauropod tracks at the Snake Creek Tracksite are preservation grade 1.0 or lower. However, a few exceptions, like AODF 904.S1.20, could conceivably be classified as preservation grade 2.0. Thus, although we can distinguish these tracks from all other named sauropod tracks worldwide, we err on the side of caution by not naming a new ichnotaxon. By contrast, virtually all the non-sauropod tracks at the Snake Creek Tracksite can easily be assigned a preservation grade of 2.0, since all are complete and well-defined, but generally lack digit pad impressions. Most of these tracks are assigned to existing ichnotaxa, although some are left unassigned.

**Figure 10:**
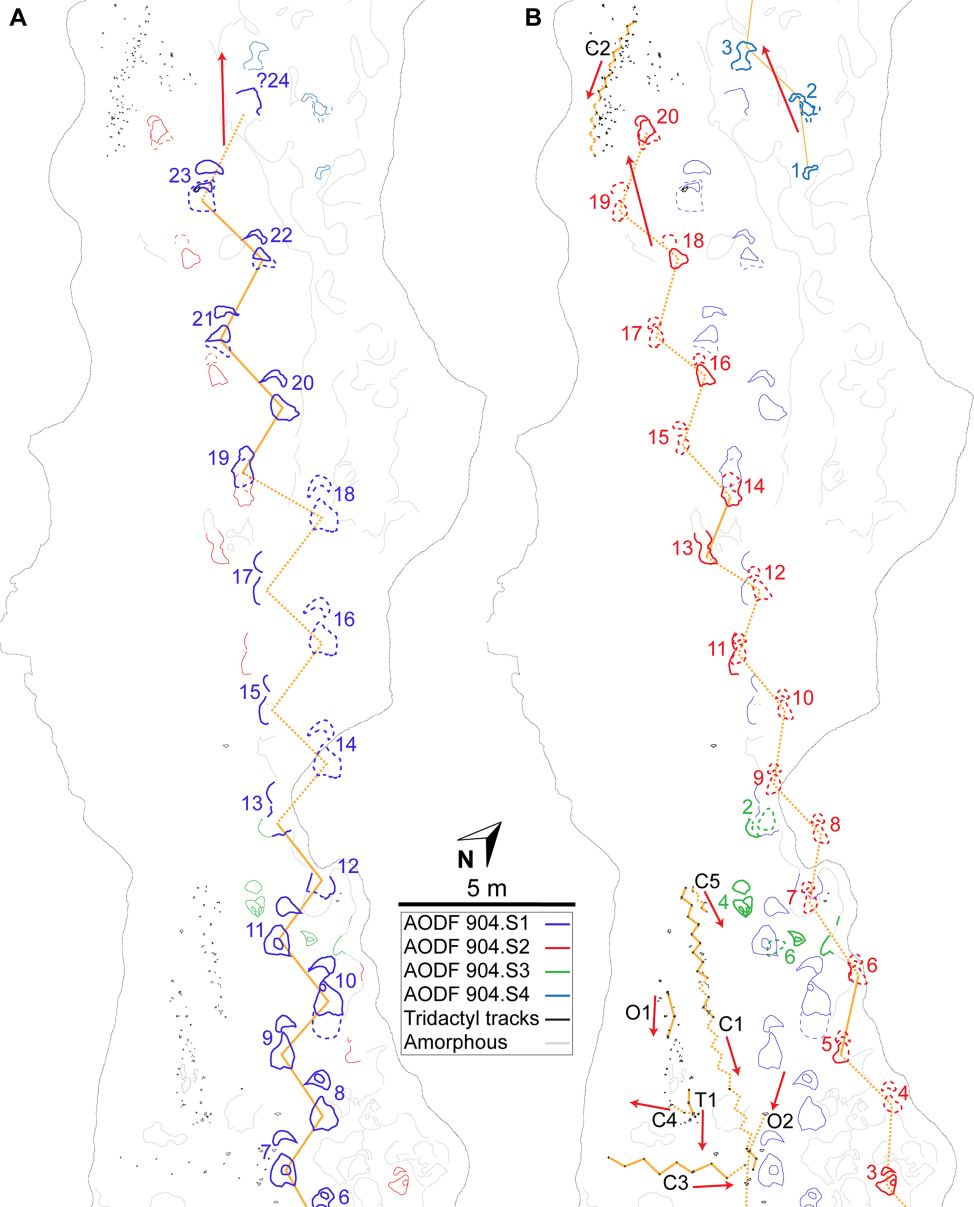
Close-up map of the central section of the Snake Creek Tracksite. (A) Close-up map with the sauropod trackway AODF 904.S1 annotated. (B) Close-up map with the sauropod trackway AODF 904.S2, sauropod “turning circle” trackway AODF 904.S3, the start of sauropod trackway AODF 904.S4, and multiple crocodyliform, theropod, and ornithopod trackways annotated. Also note the possible theropod penetrative track in AODF 904.S1.23.

The main sauropod trackway at the Snake Creek Tracksite (AODF 904.S1) extends from the southeast to the northwest (Figs. 7–8, 10). The southeast section of the site preserves sauropod tracks pertaining to at least three individuals: four tracks pertaining to the main sauropod track maker (AODF 904.S1.01–04), some tentatively attributed to a second sauropod trackway (AODF 904.S2.01–03, 05–06), and others not easily assigned to trackways. However, the tracked surface in this area is degraded or absent, presumably owing to weathering: only transmitted track walls remain. The best-preserved sauropod tracks at the Snake Creek Tracksite (AODF 904.S1.05–11) are situated immediately northwest of the indistinct sauropod track section. These sauropod tracks lack floors (adhesion traces notwithstanding) but preserve track walls and displacement rims. Two relatively well-preserved sauropod tracks in this section are distinctive: a flattened patch is present, and seemingly continuous with, the pes track of one (AODF 904.S1.10), whereas the manus track of the next (AODF 904.S1.11) has the tallest anterior displacement rim observed anywhere on site. Immediately anterior to the manus track of AODF 904.S1.10, and medial to AODF 904.S1.11, a sauropod right manus track is present (AODF 904.S3.06), with its convex surface directed to the northeast (i.e. perpendicular to the main trackway). Anterolateral to the pes track of AODF 904.S1.11, a smaller sauropod right manus–pes couplet (AODF 904.S3.04) is present, directed northwest–southeast (i.e. parallel with, but directionally opposed to, the main trackway). These smaller sauropod tracks are interpreted as being part of a turning trackway, AODF 904.S3 (Fig. 10B).

**Figure 11:**
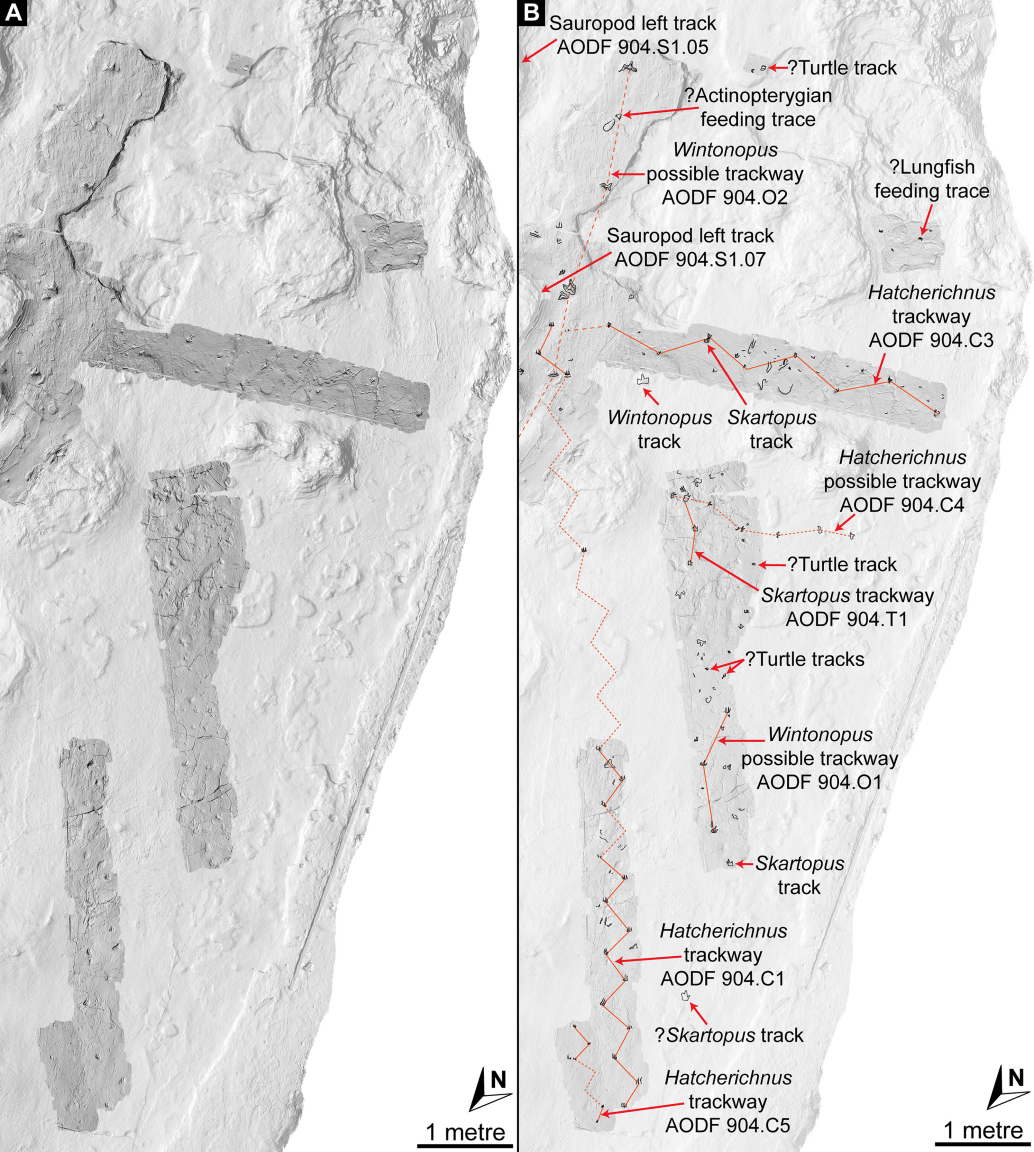
Close-up map of the southwest section of the Snake Creek Tracksite. (A) Close-up map showing laser scanned sections overlaid on the photogrammetric model. (B) Close-up map with several crocodyliform, ornithopod, and theropod trackways annotated, along with possible turtle tracks and probable lungfish and actinopterygian feeding traces.

Southwest of the best-preserved sauropod tracks (AODF 904.S1.05–11), numerous small tridactyl tracks are present (Fig. 11), including a minimum of four trackways that run parallel with, but in the opposite direction to, the sauropod trackways (i.e., northwest–southeast). Two of these, which run approximately parallel to each other, are attributable to crocodyliform trackmakers (AODF 904.C1 and AODF 904.C5); the distal tracks in the former trackway (AODF 904.C1.31 and AODF 904.C1.32) interrupt the ridges present in the displacement rim surrounding sauropod track AODF 904.S1.07, implying that this crocodyliform traversed the Tracksite after the maker of the main sauropod trackway (Fig. 9B). The other two tridactyl trackways in this section of the site, each of which is two paces long, are respectively tentatively attributed to a small-bodied theropod (AODF 904.T1) and a similarly small-bodied ornithopod (AODF 904.O1). Another series of larger ornithopod tracks might form a trackway (AODF 904.O2), but this is tentative. Two additional crocodyliform trackways preserved in this section of the site—one definite (AODF 904.C3) and one tentative (AODF 904.C4)—are directed approximately perpendicular to the other trackways (AODF 904.C3 from west–east; AODF 904.C4 from east–west). Finally, this section of the site also preserves some tridactyl and tetradactyl tracks that are tentatively ascribed to turtles, ∩-shaped feeding traces tentatively attributed to lungfish, and a possible actinopterygian fish feeding trace (Fig. 11).

Further northwest of AODF 904.S1.11, several tracks from the two main sauropod trackways are incomplete; it is presumed that they were lost, in whole or in part, to recent erosion during periodic channel reactivation in Snake Creek (although it is possible that they were never preserved). In this section of the site, the left manus–pes couplets of the main trackway (AODF 904.S1.13, 15, 17) are represented only by their lateral margins and displacement rims, whereas the corresponding right manus–pes couplets (AODF 904.S1.14, 16, 18) are missing. When their inferred positions were included based on the average stride lengths of the left manus–pes couplets, a continuous right manus–pes track sequence is apparent along the trackway. The left manus–pes couplet AODF 904.S1.13 appears to have overprinted a sauropod right manus track (AODF 904.S3.02) that is directed from northeast–southwest (perpendicular to the main trackway) and is interpreted to be part of the turning trackway AODF 904.S3.

**Figure 12:**
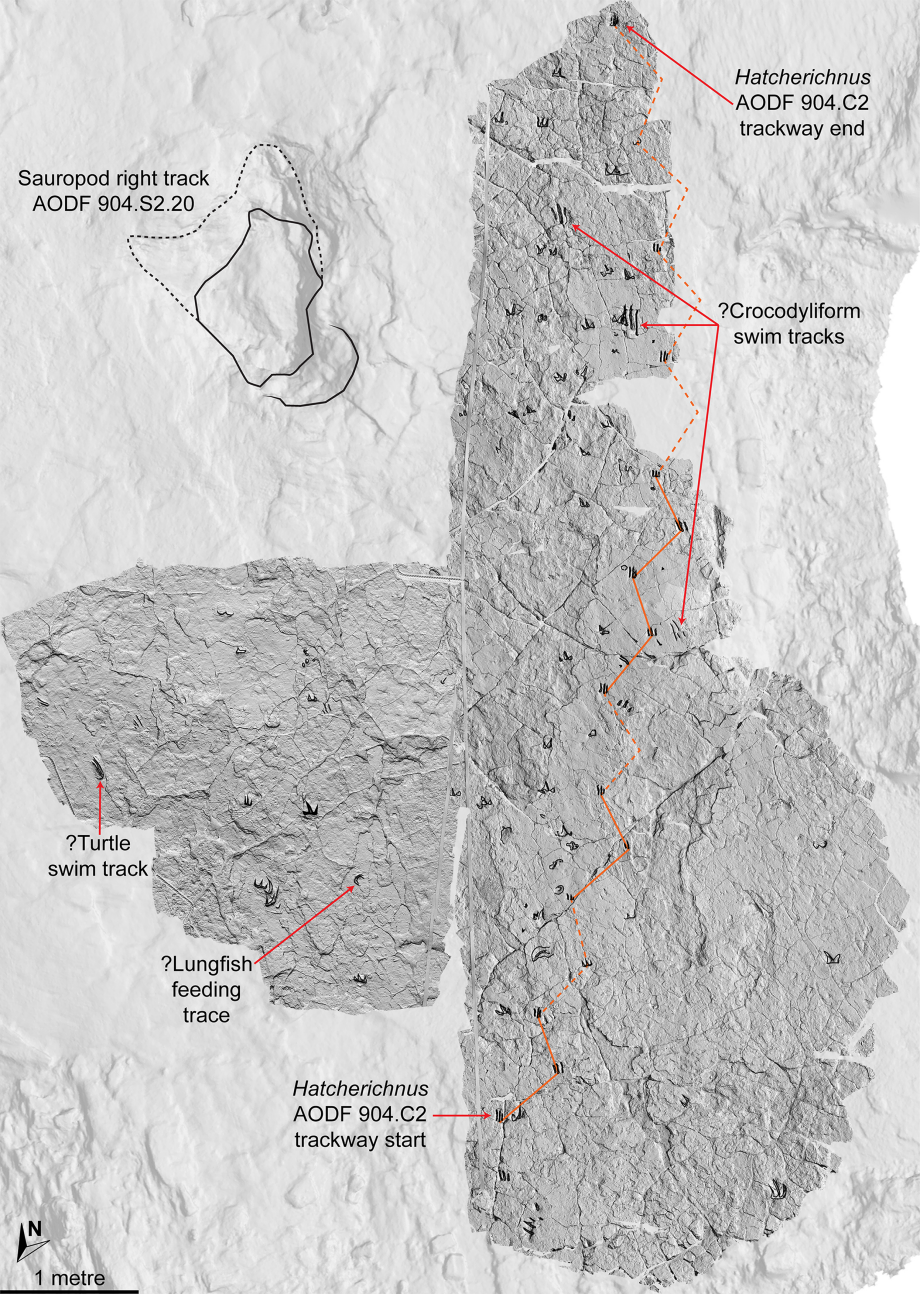
Close-up map of the northwest section of the Snake Creek Tracksite. The sauropod track AODF 904.S2.20 and the *Hatcherichnus* crocodyliform trackway AODF 904.C2 trackway are annotated, along with other isolated crocodyliform swim tracks, and possible turtle tracks and lungfish feeding traces.

In the northwest of the site, the second sauropod trackway (AODF 904.S2) runs to the left of, and practically parallel with, the first (AODF 904.S1). However, the earliest formed portions of these trackways in this section of the site intersect: the left manus–pes couplet AODF 904.S1.19 appears to have overprinted the right manus–pes couplet AODF 904.S2.14. Although this indicates that the two main sauropod trackways were not formed synchronously, their similar preservation quality implies that relatively little time separated their formation. In this section of the Snake Creek Tracksite, two manus tracks from the main sauropod trackway (AODF 904.S1.20 and AODF 904.S1.22) preserve complete track walls and floors. The right pes tracks in trackway AODF 904.S2 lack floors, whereas the associated right manus tracks preserve indistinct floors (the tracked surface proper having been mostly lost to weathering). West–northwest of the last preserved manus–pes couplet of the smaller sauropod trackway (AODF 904.S2.20), numerous small, tridactyl tracks are present, most of which are ascribed to crocodyliforms (Fig. 12). One set of small crocodyliform tracks forms a distinct trackway (AODF 904.C2), again projecting virtually parallel with, albeit in the opposite direction to, the sauropod trackways. Also in this section are numerous crocodyliform and turtle swim tracks (many of which cannot be easily organised into trackways), and ∩-shaped feeding traces attributed tentatively to lungfish. Finally, within the anterior section of the sauropod pes track AODF 904.S1.23, a possible medium-sized theropod track (directed approximately south–north) is preserved (Fig. 10B). This theropod track must have been made before sauropod track AODF 904.S1.23, since the latter appears to have caused deformation of the former.

## Description of track morphotypes

**Sauropod tracks.**

Ichnogen. et ichnosp. indet.

**Figure 13:**
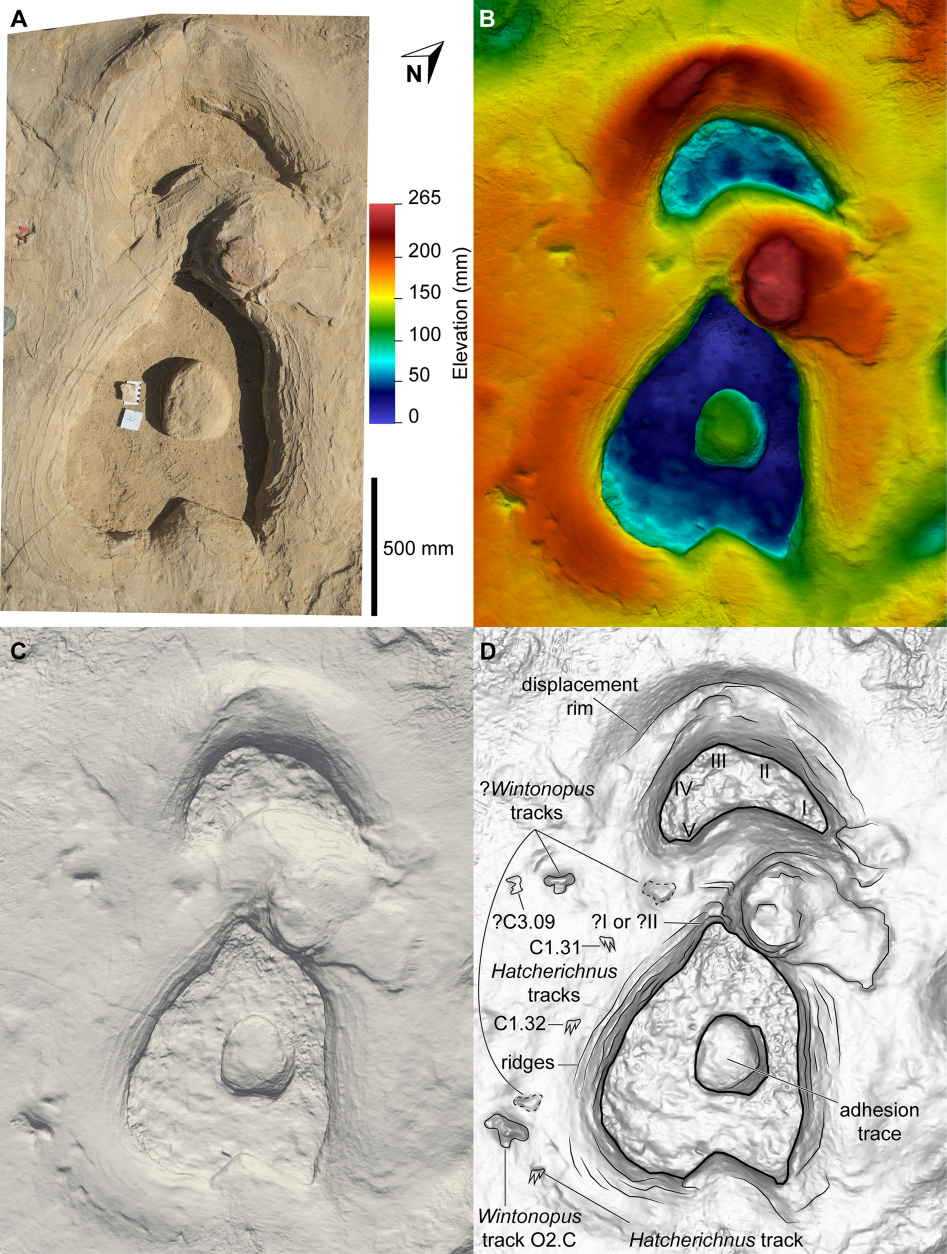
Sauropoda ichnogen. et ichnosp. indet. left manus–pes couplet AODF 904.S1.07, associated with both *Hatcherichnus* and *Wintonopus* tracks. (A) Photograph, lit from the east (lower right); (B) colour depth map; (C) monochrome surface model, lit from the northwest (top); (D) outline drawing on monochrome surface model, lit from the northwest (top).

**Figure 14:**
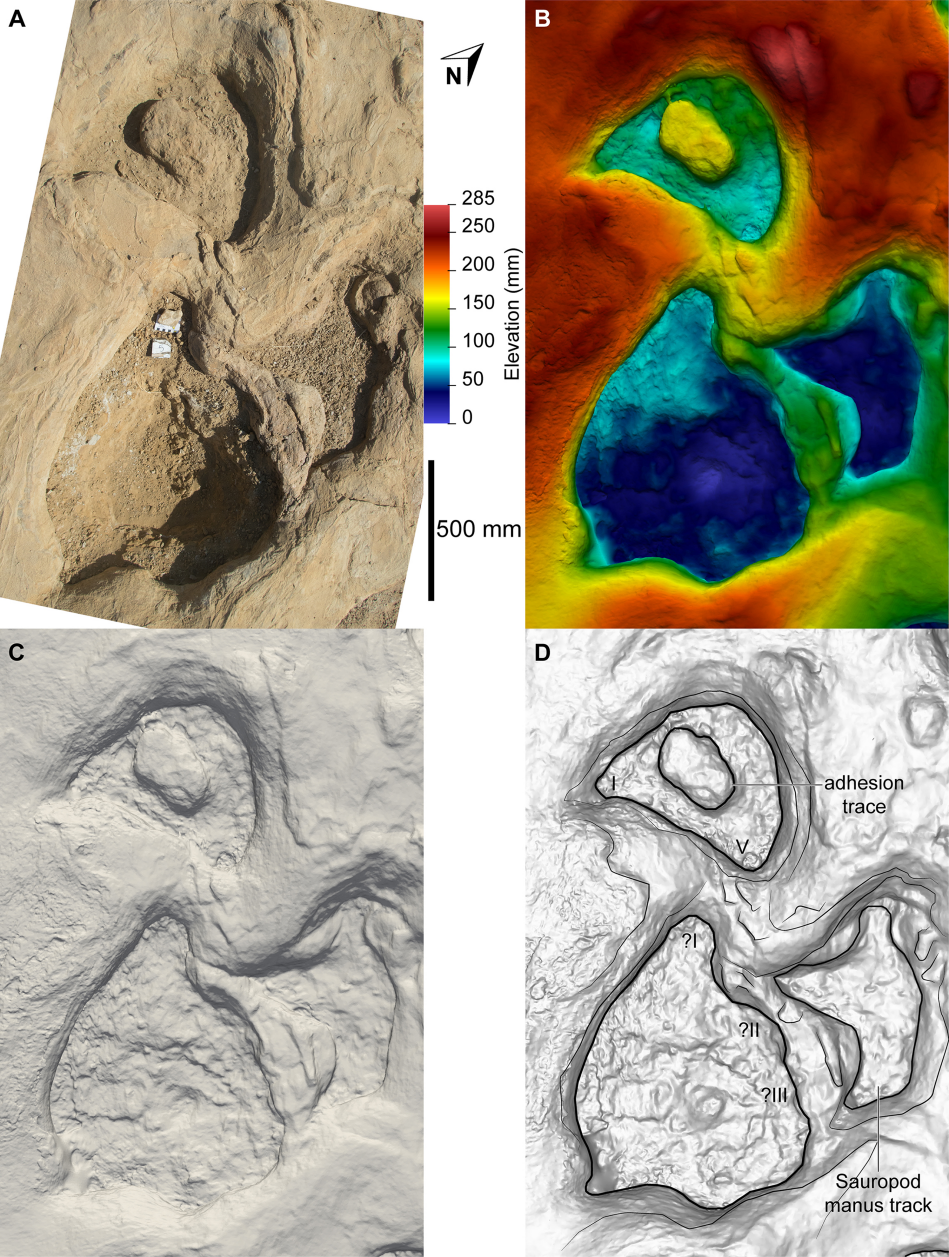
Sauropoda ichnogen. et ichnosp. indet. right manus–pes couplet AODF 904.S1.08, associated with a sauropod manus track. (A) Photograph, lit from the east (lower right); (B) colour depth map; (C) monochrome surface model, lit from the northwest (top); (D) outline drawing on monochrome surface model, lit from the northwest (top).

**Figure 15:**
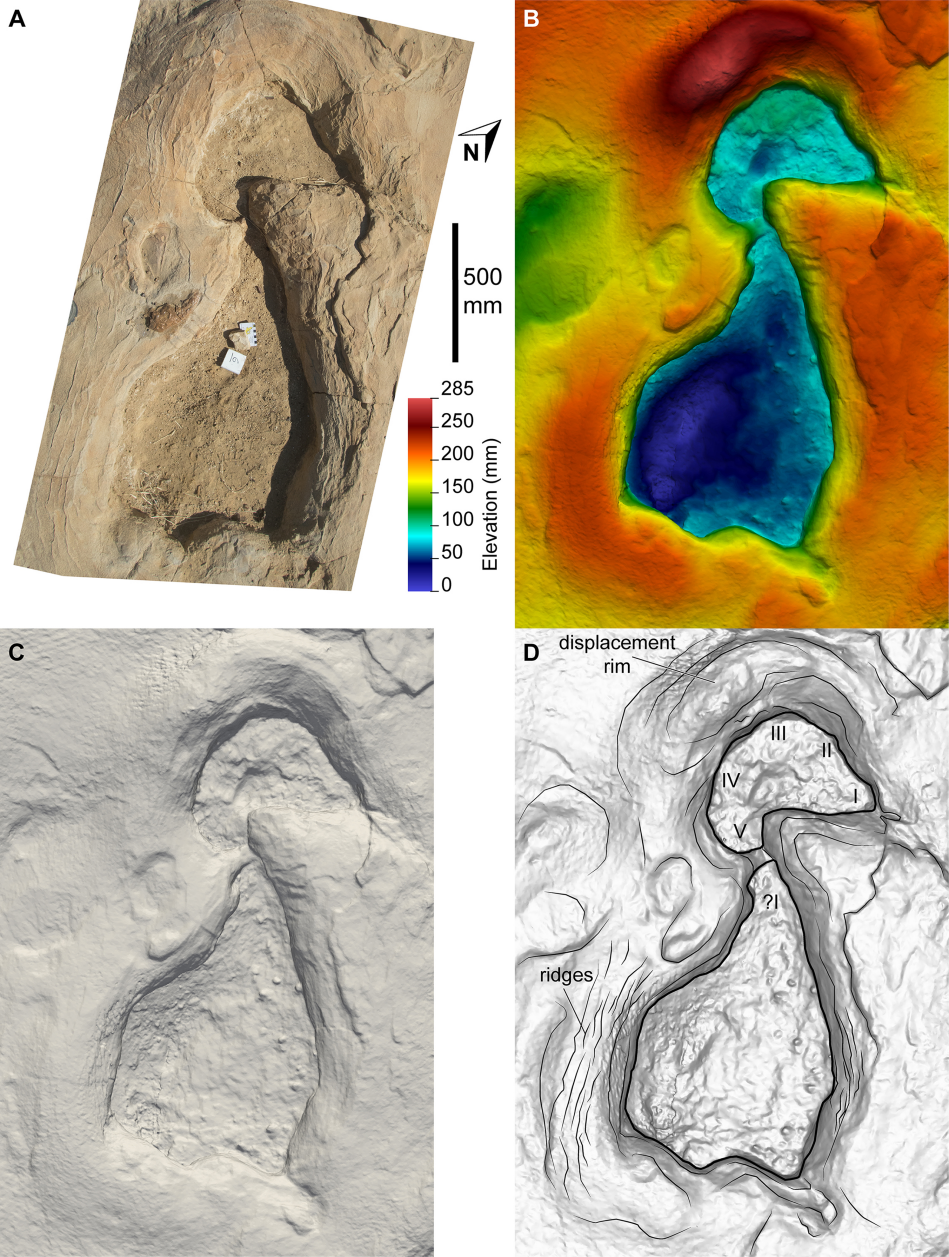
Sauropoda ichnogen. et ichnosp. indet. left manus–pes couplet AODF 904.S1.09. (A) Photograph, lit from the east (lower right); (B) colour depth map; (C) monochrome surface model, lit from the northwest (top); (D) outline drawing on monochrome surface model, lit from the northwest (top).

**Figure 16:**
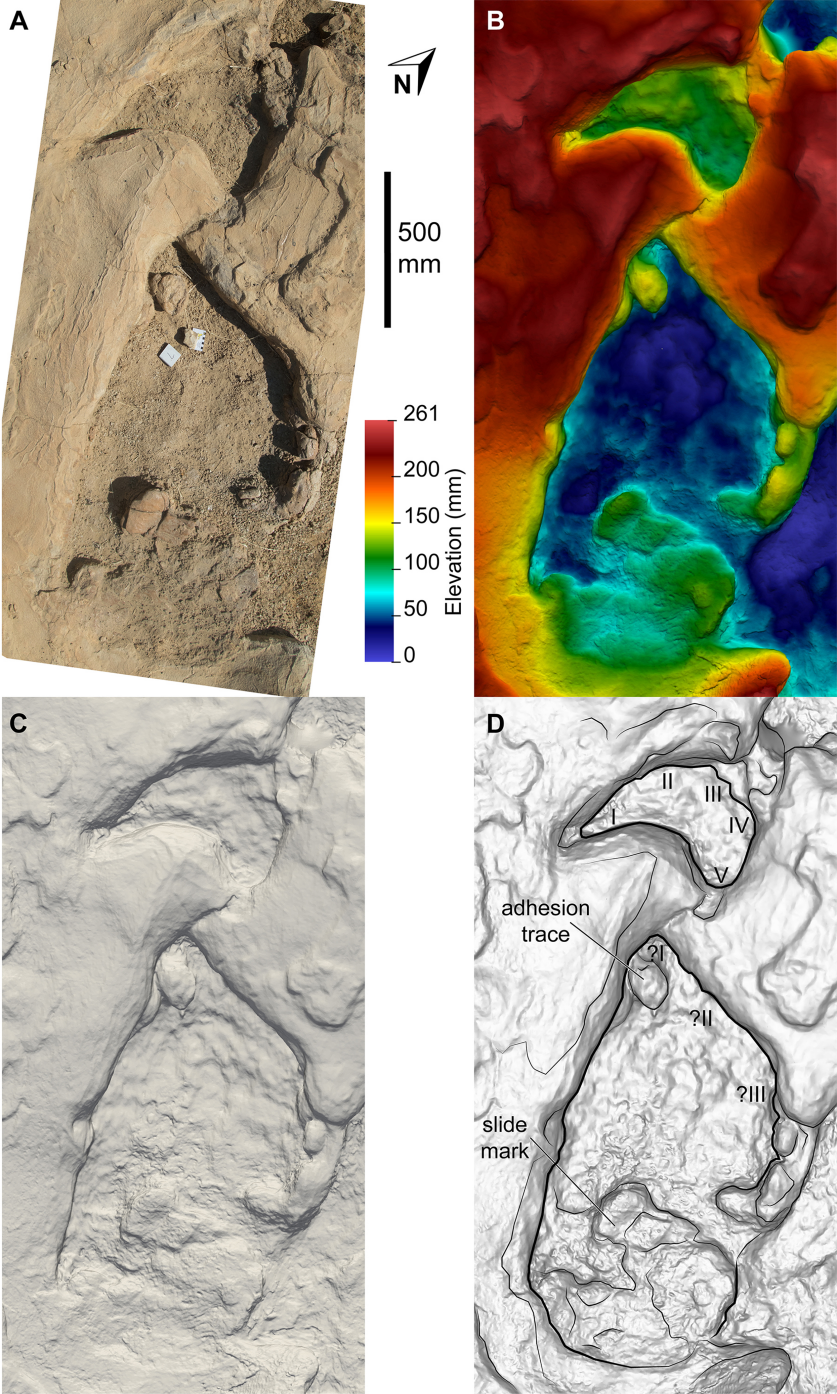
Sauropoda ichnogen. et ichnosp. indet. right manus–pes couplet AODF 904.S1.10, showing a flattened section of sediment immediately posterior to, and continuous with, the pes track. (A) Photograph, lit from the east (lower right); (B) colour depth map; (C) monochrome surface model, lit from the northwest (top); (D) outline drawing on monochrome surface model, lit from the northwest (top).

**Figure 17:**
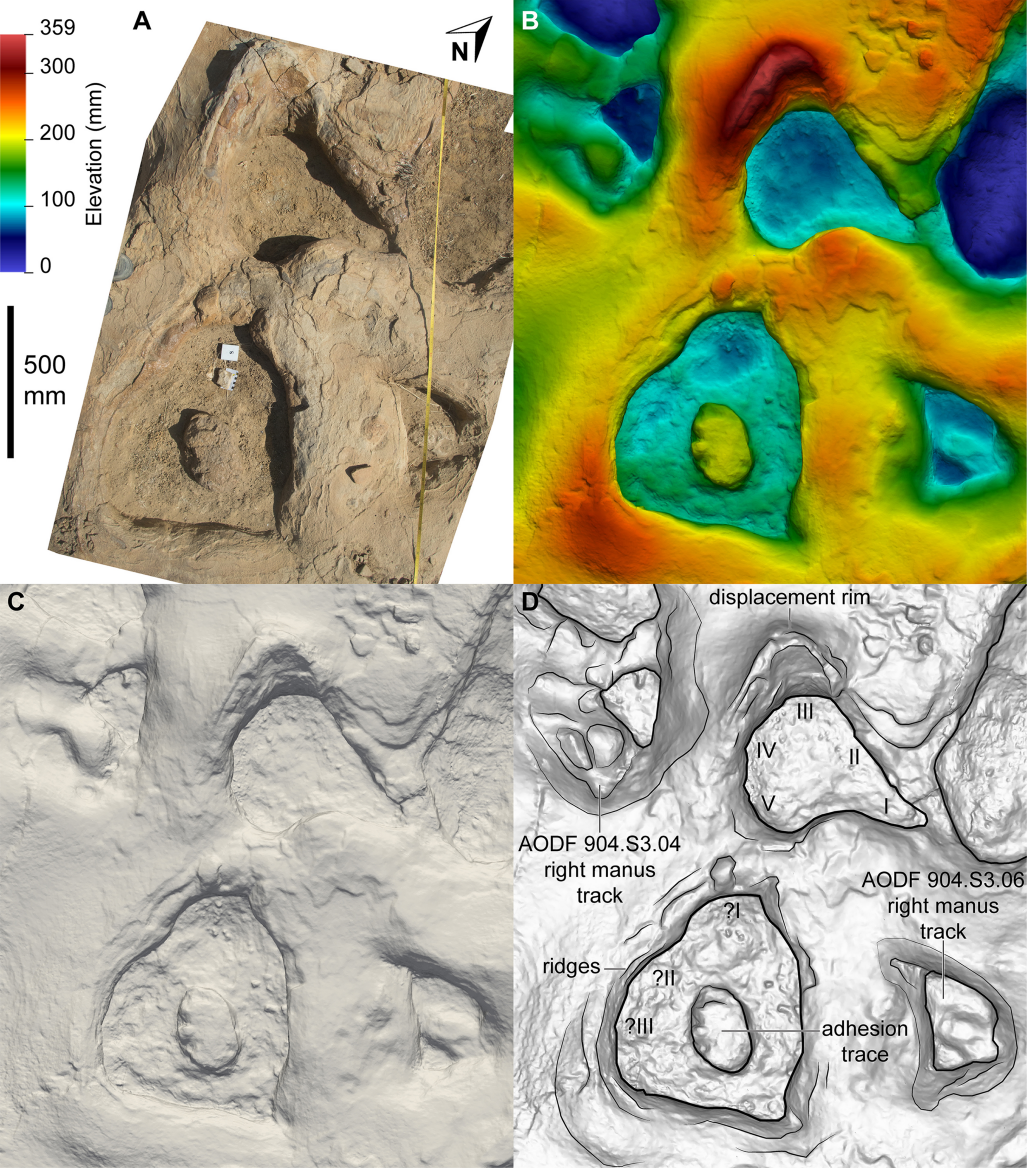
Sauropoda ichnogen. et ichnosp. indet. left manus–pes couplet AODF 904.S1.11, associated with right-sided tracks from sauropod trackway AODF 904.S3. (A) Photograph, lit from the east (lower right); (B) colour depth map; (C) monochrome surface model, lit from the northwest (top); (D) outline drawing on monochrome surface model, lit from the northwest (top).

**Figure 18:**
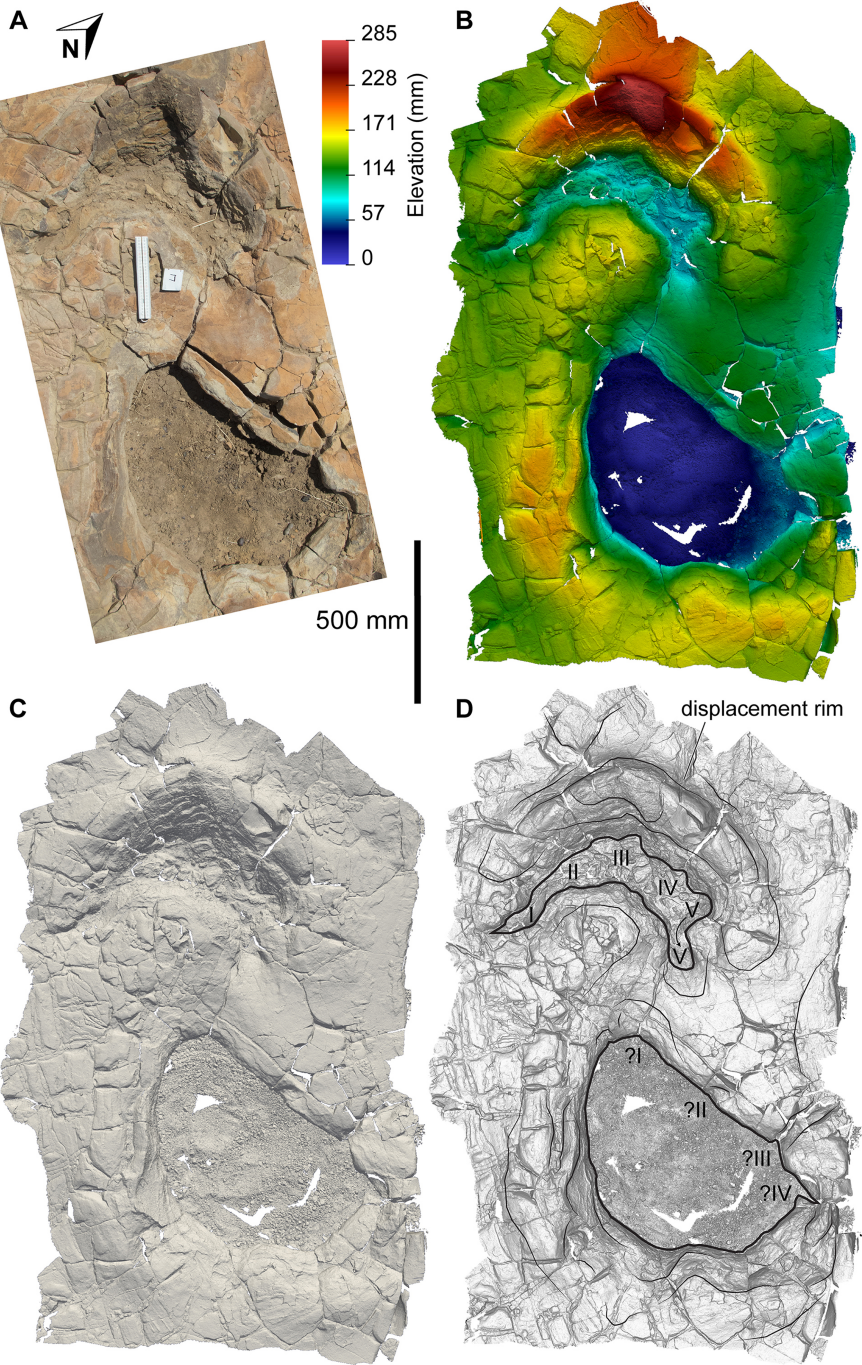
Sauropoda ichnogen. et ichnosp. indet. right manus–pes couplet AODF 904.S1.20, showing one of the few preserved sauropod manus track floors from the Snake Creek Tracksite. (A) Photograph, lit from the east (lower right); (B) colour depth map; (C) monochrome surface model, lit from the northwest (top); (D) outline drawing on monochrome surface model, lit from the northwest (top).

**Referred tracks.** AODF 904.S1—a reasonably well-preserved, essentially straight trackway (Figs. 7–8, 10, 13–18; Table 1), directed to the northwest, comprising: twelve consecutive alternating manus–pes couplets preserving track walls, displacement rims and (rarely) adhesion traces (AODF 904.S1.1–12), three incomplete consecutive left manus–pes couplets preserving track walls and displacement rims (AODF 904.S1.13, AODF 904.S1.15, and AODF 904.S1.17), and five consecutive alternating manus–pes couplets preserving track walls and displacement rims (AODF 904.S1.19–23); AODF 904.S2—a reasonably well-preserved, essentially straight trackway (Figs. 7–8, 10; Table 1), directed to the northwest, comprising: three consecutive alternating tracks (AODF 904.S2.01–03), a gap equivalent to one right track, two consecutive alternating tracks (AODF 904.S2.05–06), a gap equivalent to five consecutive alternating tracks, two consecutive left tracks (AODF 904.S2.11 and AODF 904.S2.13), and four consecutive right tracks (AODF 904.S2.14, AODF 904.S2.16, AODF 904.S2.18, and AODF 904.S2.20); AODF 904.S3—an anticlockwise ‘turning’ sauropod trackway (Figs. 7–8, 17), comprising three consecutive right tracks (AODF 904.S3.02, AODF 904.S3.04 and AODF 904.S3.06); AODF 904.S4—a short and somewhat dubious trackway in the northeast of the site (Figs. 7–8, 10B); and other isolated sauropod tracks (Figs. 7–8).

**Table 1:** Snake Creek Tracksite sauropod track measurements (all in mm).

AODF 904.S1	PL	PW	ML	MW	HI	WAP	ANG	TF–TT	AMSL	PMSL	APSL	PPSL
04	1005	875	480	618	39.1	–	–	04–06	3,000	3,100	3,300	3,130
05	924	720	495	530	28.2	–	–	05–07	3,320	3,500	3,280	3,320
06	980	670	310	560	42.2	–	–	06–08	3,340	3,240	3,390	3,350
07	945	710	392	630	43.5	–	–	07–09	3,280	3,160	3,330	3,300
08	995	800	465	630	51.9	1.17	96°	08–10	3,230	3,220	3,180	3,250
09	1050	650	470	580	38.9	1.25	105°	09–11	3,390	3,260	3,380	3,240
10	1600† (975)	815	700	470	37.5	1.37	110°	10–12	–	–	–	3,150
11	825	710	550	700	46.6	–	–	11–13	3,340	3,250	3,500	3,330
12	–	785	–	–	–	–	–	–	–	–	–	–
13	870	–	530	–	–	–	–	13–15	–	–	3,300	3,120
15	830	–	–	–	–	–	–	–	–	–	–	–
17	–	–	–	–	–	–	–	17–19	–	–	–	3,280
19	860	565	440	610	–	–	–	–	–	–	–	–
20	750	670	400	710	38.9	–	–	20–22	4,000	3,900	4,040	4,040
21	945	590	370	630	34.3	–	–	21–23	4,110	4,070	4,140	4,010
22	910	650	360	590	28.9	–	–	–	–	–	–	–
23	865	670	370	710	43.6	–	–	–	–	–	–	–
Mean	915.3	705.7	452.3	612.9	39.5	1.26	103.7	Mean	3,445.6	3,411.1	3,484	3,376.7
**AODF 904.S2**	**PL**	**PW**	**ML**	**MW**	**HI**	**WAP**	**ANG**	**TF–TT**	**AMSL**	**PMSL**	**APSL**	**PPSL**
11	700	–	390	–	–	–	–	11–13	–	–	–	3,260
13	460	405	–	430	–	–	–	13–15	–	–	–	–
14	–	605	–	–	–	–	–	14–16	–	–	–	3,430
16	530	450	300	490	–	–	–	16–18	3,480	–	3,380	3,360
18	550	495	350	515	–	–	–	18–20	–	3,740	–	3,680
20	610	440	290	400	–	–	–	–	–	–	–	–
Mean	570	479	332.5	458.75	–	–	–	Mean	3,480	3,740	3,380	3,432.5

**Note:**

Abbreviations as follows: PL, pes length (anteroposterior); PW, pes width (mediolateral); ML, manus length (anteroposterior); MW, manus width (mediolateral); HI, heteropody index (percentage calculated from track outlines in Adobe Illustrator); WAP, width of the pedal track angulation pattern; ANG, pace angulation; TF–TT, track measured from–track measured to; AMSL, anterior manus stride length (measured between anterior margins of successive ipsilateral manus tracks); PMSL, posterior manus stride length (measured between posterior margins of successive ipsilateral manus tracks); APSL, anterior pes stride length (measured between anterior margins of successive ipsilateral pes tracks); PPSL, posterior pes stride length (measured between posterior margins of successive ipsilateral pes tracks). The measurement marked with a dagger (†) includes a flattened section of sediment immediately posterior to the pes track; the measurement excluding this flattened sediment section appears immediately below.

**Horizon and locality.** ‘Upper’ Winton Formation, Cenomanian–?lowermost Turonian (Tucker et al., 2013); Snake Creek Tracksite (AODL 251), Karoola Station, NW of Winton, Queensland, Australia.

**Description.** Although the sauropod trackways at the Snake Creek Tracksite appear to show a unique combination of characters (mild–moderate heteropody (~40%) sensu Salisbury et al. (2017); manus tracks with clear digit I ungual impressions; wide-gauge trackway (manus width: trackway width ratio ~28%; pes width: trackway width ratio ~32%)), they are herein left unassigned to an ichnotaxon because of their preservation quality (Marchetti et al., 2019). The most prominent sauropod trackways at the site are two mostly parallel, northwest-trending trackways (AODF 904.S1 and AODF 904.S2). These are supplemented by an abbreviate trackway made by a turning sauropod (AODF 904.S3), an indistinct and incomplete trackway (AODF 904.S4), and other isolated sauropod tracks at the Snake Creek Tracksite. The description below is based on AODF 904.S1, except where noted.

Based on the average stride length of the preserved tracks, the complete AODF 904.S1 trackway (as constrained herein) would have comprised 24 alternating manus–pes couplets (Figs. 8C, 10A). All of the manus tracks in the AODF 904.S1 trackway are crescentic to semi-circular, broadly conforming with sauropod manus tracks generally (Milàn, Christiansen & Mateus, 2005; Thulborn, 1990; Wright, 2005). They do not match any of the manus track morphologies identified by Castanera et al. (2016) from the Iberian Peninsula (although the absence of Albian–Campanian-aged tracks in their sample might explain this). All but one of the sauropod manus tracks at the Snake Creek Tracksite is wider mediolaterally than it is long anteroposteriorly (Table 1; average manus length (ML)/manus width (MW) = 0.73); the sole exception is the manus track AODF 904.S1.11 (Fig. 17), which would have been formed either concurrently with, or shortly after, the most structurally divergent pes track (AODF 904.S1.10) in the trackway (Fig. 16; see below). This ratio is closer to that obtained from reniform sauropod manus tracks from the Tithonian–Berriasian of the Iberian Peninsula than to Aptian and Maastrichtian tracks from the same region (Castanera et al., 2016). Within the AODF 904.S1 trackway, manus track length ranges from 310–550 mm (notwithstanding the outlier AODF 904.S1.10, wherein this measurement is 700 mm), and manus track width ranges from 470–710 mm (Table 1). Although few of the manus tracks preserve more than track walls and central adhesion traces, there are two notable exceptions: AODF 904.S1.20 and AODF 904.S1.22, which represent successive right manus tracks. Of these, AODF 904.S1.20 is better preserved and more complete.

Impressions of five digits appear to be preserved in the floor and wall of the manus track of AODF 904.S1.20 (Fig. 18). The impression of digit I is posteromedially directed, triangular, and medially tapered, in both AODF 904.S1.20 and in less complete and less well-preserved tracks (e.g. AODF 904.S1.07 (Fig. 13); AODF 904.S1.08 (Fig. 14)), irrespective of the manus–pes (MP) interautopodial distance (see below). In tracks where this feature appears relatively reduced (e.g. AODF 904.S1.07–09), there is evidence to suggest that substrate rebound occurred: the sediment situated at the posteromedial extremity of the manus track in each case appears ‘pinched-in’ (Figs. 13–15). On the balance of the evidence available, we suggest that a manual pollex ungual was present in the trackmaker. The remaining four manual digit impressions that we suggest are present in AODF 904.S1.20 are short, broad, and rounded, with their respective external margins directed anteromedially (II), anteriorly (III), anterolaterally (IV) and laterally–posterolaterally (V). Two impressions corresponding to digit V appear to be present: one situated posteromedial to the other. The posteromedially situated impression is consistently shallower than the impressions of all other digits, whereas the depth of its counterpart is comparable to those of the more medial digits. The posterior margin of digit V is often situated further posteriorly than the posterior margin of digit I (e.g. AODF 904.S1.09 (Fig. 15)).

All of the pes tracks in the AODF 904.S1 trackway are longer anteroposteriorly than they are mediolaterally wide (Table 1; average pes length (PL)/pes width (PW) = 1.30). Although the longest pes track (AODF 904.S1.10 (Fig. 16)) is almost 600 mm longer than the next longest pes track (AODF 904.S1.09 (Fig. 15)), it should be noted that this anomalous measurement includes a 625 mm-long, flattened section at the posterior end that appears to represent a layer of sediment compacted by another sauropod that traversed this section of the site prior to the AODF 904.S1 track maker. This interpretation is borne out by the presence of another seemingly overprinted sauropod track, the vestiges of which can be identified immediately lateral to the pes track of AODF 904.S1.09 (Fig. 15B). All other pes tracks in the AODF 904.S1 trackway are similar in size to AODF 904.S1.09 (Table 1). Most pes tracks comprise track walls and displacement rims, with subcircular median adhesion traces irregularly present (see below). However, there are some notable exceptions: the track floors of the pes tracks of AODF 904.S1.10 (posterior third; Fig. 16) and AODF 904.S1.22 (posterior two-thirds) are preserved; and the shallowly-impressed pes track of AODF 904.S1.23 is complete, albeit indistinct. The few preserved track floors do not exhibit skin impressions or foot topography; they are generally smooth, with some degree of sediment compaction. Where preserved, pes track floors are deeper anteriorly than posteriorly. Few of the AODF 904.S1 pes tracks provide clear evidence of digits, beyond an anterior taper that was probably caused by a dragging digit I ungual (or possibly, but less likely, digit II ungual). Each pes track is broadest at or near the posterior margin. The posterior margin is essentially flat in most pes tracks, but concave forwards in others.

Displacement rims are present around many of the AODF 904.S1 tracks. These are especially prominent along the anterior margin of the manus tracks, and along the anterior and lateral margins of the pes tracks. The best-preserved displacement rims (e.g. those surrounding left manus–pes couplets AODF 904.S1.07 (Fig. 13) and AODF 904.S1.09 (Fig. 15)) host numerous ridges that run approximately parallel with the external margin of the track and with each other. Consequently, the best-preserved tracks are bordered, in whole or in part, by several effectively concentric rings of variable extent.

Several manus tracks and many pes tracks were found with rounded lithified bodies present centrally. These do not contact the external margins of the tracks, and are underlain by weathered, unconsolidated sedimentary strata. They are interpreted herein as adhesion traces sensu Carey et al. (2011), equivalent to the “pressure pads” of Fornós et al. (2002). In the manus tracks, the adhesion traces are situated centrally, approximately in line with where digit III would have been (e.g. AODF 904.S1.08 (Fig. 14)), whereas in the pes tracks the adhesion traces are situated approximately at the mid-length, equidistant from the lateral and medial margins (e.g. AODF 904.S1.07 (Fig. 13); AODF 904.S1.11 (Fig. 17)). The approximately central position of the adhesion traces within each track implies that the slope of the tracked surface was negligible at the time of track formation.

Based on length and width measurements of the manus and pes tracks (Table 1), the heteropody index (HI) sensu González Riga & Calvo (2009) of the AODF 904.S1 trackway is 42.9%, thereby constituting mild heteropody sensu Salisbury et al. (2017). By contrast, the average HI based on areas of track outlines sketched on aerial photographs of the tracks in Adobe Illustrator (Figs. 8, 10) is 39%, which constitutes moderate heteropody sensu Salisbury et al. (2017). Given that these calculations of HI index in AODF 904.S1 fall effectively on the boundary between mildly or moderately heteropodous sensu Salisbury et al. (2017), it seems prudent not to assign them to either category at this stage, especially given that the track dimensions vary substantially along the trackway.

The average stride length measured on the AODF 904.S1 trackway—derived for both manus and pes tracks—was 3.43 m (Figs. 7–8, 10; Table 1). The average external trackway width, which varies only slightly along the length of the trackway, is 2.2 m. Using this value, and the average values of MW and PW, the average manus width: trackway width ratio (MTR) is 27.9%, whereas the average pes width: trackway width ratio (PTR) is 32.1%. Using either of these measurements, the AODF 904.S1 trackway is wide-gauge sensu Romano, Whyte & Jackson (2007), since neither manus nor pes tracks overlap the midline (Farlow, 1992; Lockley, Farlow & Meyer, 1994a; Wilson & Carrano, 1999). The lateral margins of the manus tracks are invariably situated nearer to the midline than those of the pes tracks.

The width of the pedal track angulation pattern (WAP) varies slightly along the only section of the trackway on which it is measurable (i.e., tracks AODF 904.S1.07–11). The WAP values in this section range from 1.17 to 1.37 m, whereas the pace angulation (ANG) ranges from 96° to 110° (Table 1). The WAP/PL ratio (Marty, 2008) increases slightly in the measured section, from 1.11 (AODF 904.S1.08) to 1.19 (AODF 904.S1.09) to 1.40 (AODF 904.S1.10).

In the AODF 904.S1 trackway, ipsilateral manus and pes tracks made at the end of the same step cycle are generally situated close to one another, with the posterior margin of the manus track level (or, rarely, confluent) with the anterior margin of the pes track (Figs. 6–8, 10). By contrast, the distance between consecutive ipsilateral manus–pes couplets (measured from the anterior margin of the manus track in the first couplet to the posterior margin of the pes track in the second) often equals or exceeds the overall length of an individual manus–pes couplet (Fig. 10). Thus, the AODF 904.S1 trackway is characterised by having short manus–pes (MP) interautopodial distances (within individual couplets) and long pes–manus (PM) interautopodial distances (between two successive ipsilateral couplets) sensu Salisbury et al. (2017).

**Comparisons.** The presence of a manual pollex ungual impression differentiates the AODF 904.S1 trackway from *Occitanopodus gandi* (Moreau et al., 2020), *Oobardjidama foulkesi* (Salisbury et al., 2017), *Titanosaurimanus nana* (Dalla Vecchia & Tarlao, 2000), *Brontopodus plagnensis* (Mazin, Hantzpergue & Olivier, 2017), *Brontopodus pentadactylus* (Kim & Lockley, 2012), *Brontopodus birdi* (Farlow, Pittman & Hawthorne, 1989), *Sauropodichnus giganteus* (Calvo, 1991; Calvo, 1999), *Titanopodus mendozensis* (González Riga & Calvo, 2009; González Riga, 2011; González Riga & Tomaselli, 2019) and *Calorckosauripus lazari* (Meyer, Marty & Belvedere, 2018). By contrast, *Polyonyx gomesi* and *Polyonyx* isp. from the Middle Jurassic of Portugal (Dos Santos et al., 1994; Santos, Moratalla & Royo-Torres, 2009), cf. *Polyonyx* from the Middle–?Upper Jurassic of Morocco (Oukassou et al., 2019), a sauropod manus cast from the Upper Jurassic of Portugal (Milàn, Christiansen & Mateus, 2005), and an un-named sauropod track from the Early Cretaceous of Italy (Dalla Vecchia, 1999; Dalla Vecchia & Tarlao, 2000), all preserve evidence of a prominent ungual on manual digit I.

Impressions of manual digits II–V, which are visible in some tracks in the AODF 904.S1 trackway, are also evident in *Polyonyx gomesi*, but are much more distinct in this ichnotaxon (Santos, Moratalla & Royo-Torres, 2009). The degree of lateral rotation of the manus tracks in the AODF 904.S1 trackway is far lower than in many sauropod trackways, such as those from the ?Middle Jurassic of Morocco (Lallensack et al., 2019), the Berriasian tracks of Las Cerradicas Tracksite in Spain (Castanera et al., 2011), the mid-Cretaceous Hangsheng Formation of China (Xing et al., 2019b), and *Titanopodus mendozensis* (González Riga & Calvo, 2009; González Riga & Tomaselli, 2019).

The effective absence of distinct ungual impressions in the pes tracks of the AODF 904.S1 trackway distinguishes it from many sauropod tracks, such as *Occitanopodus gandi* (Moreau et al., 2020), *Polyonyx gomesi* (Santos, Moratalla & Royo-Torres, 2009), unnamed sauropod tracks from Niger (Ginsburg et al., 1966), unnamed sauropod tracks from Spain (Pascual Arribas et al., 2008; Pascual-Arribas & Hernández-Medrano, 2010), *Brontopodus birdi* (Farlow, Pittman & Hawthorne, 1989), *Brontopodus pentadactylus* (Kim & Lockley, 2012), and unnamed titanosaur tracks from the Anacleto Formation of Mendoza, Argentina (González Riga et al., 2015). Although the near absence of distinct digit impressions in the track walls is noteworthy, it is unclear whether or not this is a consequence of poor preservation, reduced pedal digits in the trackmaker, distortion caused by the movement of the pedal unguals during the step cycle, or a combination of some or all of these factors. The pes tracks of the AODF 904.S1 trackway, which taper anteriorly and have flat to anteriorly concave posterior margins, differ from those of many other sauropod ichnotaxa. Several of these have anteriorly-broad, piriform pes tracks, including *Polyonyx gomesi* (Santos, Moratalla & Royo-Torres, 2009), *Brontopodus birdi* (Farlow, Pittman & Hawthorne, 1989) and *Oobardjidama foulkesi* (Salisbury et al., 2017), whereas other have subcircular to oval pes tracks, like *Occitanopodus gandi* (Moreau et al., 2020). Some of the sauropod pes tracks from the Briar Site in Arkansas (Pittman & Gillette, 1989) bear a distinct similarity to those of the AODF 904.S1 trackway, inasmuch as they are broadest posteriorly, tapered anteriorly (albeit to a lesser degree), and have posterior margins that are, at least in part, concave forwards. However, the concave portion of the posterior margin of the Briar Site tracks is generally limited to the lateral half, rather than situated centrally as in the AODF 904.S1 trackway. A sauropod trackway (#20) from the Era del Peladillo 3 Tracksite in Spain also has pes tracks that are broadest near their posterior margins (Casanovas et al., 1995).

The borderline mild/moderate heteropody displayed in the AODF 904.S1 trackway is in sharp contrast to the pronounced heteropody seen in, for example, *Occitanopodus gandi* (Moreau et al., 2020). The short MP interautopodial distances and long PM interautopodial distances in the AODF 904.S1 trackway distinguish it from *Titanopodus mendozensis* (González Riga & Calvo, 2009; González Riga, 2011; González Riga & Tomaselli, 2019), wherein the PM interautopodial distance exceeds the length of the largest pes track, and from *Oobardjidama foulkesi* (Salisbury et al., 2017) and a referred trackway of *Sauropodichnus giganteus* (Calvo, 1999; Calvo & Mazzetta, 2004), wherein the PM interautopodial distance is almost equivalent to the length of an average-sized pes track.

The wide-gauge nature of the AODF 904.S1 trackway distinguishes it from narrow-gauge sauropod trackways like *Breviparopus taghbaloutensis* (Dutuit & Ouazzou, 1980), *Parabrontopodus mcintoshi* (Lockley, Farlow & Meyer, 1994a), and a trackway from the Cenomanian of Argentina (Heredia et al., 2019). Trackways assigned to *Titanopodus mendozensis* (González Riga & Calvo, 2009; González Riga, 2011; González Riga & Tomaselli, 2019), as well as unnamed trackways from the Fumanya site in Spain (Vila et al., 2013), are relatively wider-gauge (with significantly lower MTR and PTR values) than the AODF 904.S1 trackway. Following the table presented by Romano, Whyte & Jackson (2007), the Cretaceous sauropod trackway with MTR and PTR values closest to those of the AODF 904.S1 trackway is the VA13 *Brontopodus*-like trackway from La Rioja, Spain (Casanovas et al., 1997; Moratalla et al., 1994; Pérez-Lorente, 2015). The slight variation in the width of the AODF 904.S1 trackway is not as marked as that seen in some other sauropod trackways, like that of *Parabrontopodus distercii* (Meijide Fuentes, Fuentes Vidarte & Meijide Calvo, 2001; Castanera et al., 2012; Stevens, Ernst & Marty, 2016).

**Locomotion of the AODF 904.S1 sauropod trackmaker.** As mentioned above, some manus tracks appear to preserve two impressions that correspond to digit V, suggesting that this digit shifted closer to the ‘palm’ as the forefoot was lifted from the substrate. The AODF 904.S1.20 manus track implies that the central three digits (II–IV) were more tightly bound than the outer digits (I and V) when the foot was placed on the ground. Such a morphology has been noted in *Brontopodus birdi* (Farlow, Pittman & Hawthorne, 1989) and many other wide-gauge sauropod trackways thought to pertain to titanosauriform sauropods (Platt & Hasiotis, 2006; Wilson & Carrano, 1999). If the binding of the metacarpals in the AODF 904.S1 trackmaker was similar to that evident in the *Brontopodus birdi* trackmaker (Farlow, Pittman & Hawthorne, 1989), then the removal of the foot from the tacky substrate might have caused the adhesion trace to be concentrated behind the middle three digits.

The anterior tapering of the pes tracks in the AODF 904.S1 trackway, which seldom interrupts the manus tracks (e.g., AODF 904.S1.09 (Fig. 15)), was presumably caused by drag from pedal ungual I (or, far less likely, II) when the foot was lifted from the substrate and propelled forward. The fact that some sauropod trackways, notably those assigned to *Polyonyx gomesi* (Dos Santos et al., 1994; Santos, Moratalla & Royo-Torres, 2009), possess pes tracks with digit I directed anteriorly (Bonnan, 2005) supports the interpretation that these drag marks were caused by pedal digit I. Although the presence of pads of soft tissue underlying the pedes of sauropods is well-established (Farlow, Pittman & Hawthorne, 1989; Gallup, 1989; Hall, Fragomeni & Fowler, 2016; Jannel et al., 2019; Platt & Hasiotis, 2006; Romano, Whyte & Manning, 1999; Thulborn, 1990), the degree of flexibility of each pad, particularly the plantar one, has not been constrained. The anteriorly concave posterior margins of many of the pes tracks, coupled with the presence of median adhesion traces, makes it tempting to hypothesise that the pedal soft tissue pad of the AODF 904.S1 trackmaker was quite malleable. However, the anterior tapering of the pes tracks implies that the substrate was partially, if not mostly, responsible for the unusual morphology of the pes tracks of the AODF 904.S1 trackmaker. The irregular distribution of the adhesion traces within the AODF 904.S1 trackway, their almost total absence from the AODF 904.S2 trackway, and their complete absence from tracks with track floors, supports this hypothesis. Similarly, the concave forward posterior margins of some pes tracks are most likely attributable to substrate rebound: whereas the heel was lifted before the digits, allowing the posterior margin to deform in a ductile fashion, at least one pedal ungual dragged through the anterior rim, preventing the same sort of rebound from occurring here.

**Trackmaker interpretation.** The somewhat unusual morphology of the AODF 904.S1 tracks relative to other Cretaceous sauropod tracks appears to be at least partly attributable to the anatomy of the trackmaker. Prior to the discovery of the Snake Creek Tracksite, the only known sauropod trackways that were both wide-gauge and had manus tracks with impressions of an ungual on digit I were those of *Polyonyx gomesi*, an ichnotaxon known only from the Middle Jurassic and definitively not made by a titanosaur (Santos, Moratalla & Royo-Torres, 2009). Following Day et al. (2004), the sauropod tracks from the Snake Creek Tracksite are intermediate between Categories 2 and 3: they are undoubtedly wide-gauge (as in Category 3), but also preserve pollex ungual impressions (some tracks in Category 2). Wright (2005) predicted that titanosaur trackways should be wide-gauge and lack manual digit impressions; however, it has now been established that at least some basal titanosaurs possessed manual phalanges on several digits (Hocknull et al., 2009; Poropat et al., 2015b; Poropat et al., 2016; Poropat et al., 2020), meaning that Wright’s (2005) prediction might hold true only for derived titanosaurs (González Riga et al., 2019).

Several unusual features of the morphology of the AODF 904.S1 trackway are congruent with the skeletal anatomy of the early-branching titanosaurs *Diamantinasaurus matildae* and *Savannasaurus elliottorum*, both of which are represented by body fossils from the Winton Formation. Both *Diamantinasaurus* and *Savannasaurus* had manual phalanges (Hocknull et al., 2009; Poropat et al., 2015b; Poropat et al., 2016; Poropat et al., 2020), and a manual phalangeal formula of 2-1-1-1-1—including a prominent ungual phalanx on digit I—has been proposed for *Diamantinasaurus* specifically (Hocknull et al., 2009; Poropat et al., 2015b). Furthermore, both *Diamantinasaurus* and *Savannasaurus* had wide-gauge stances (Klinkhamer et al., 2018; Klinkhamer et al., 2019; Poropat et al., 2015b; Poropat et al., 2016; Poropat et al., 2020). Thus, one of these taxa might have been the AODF 904.S1 trackmaker. Pelvic elements of the third Winton Formation sauropod, the early-branching somphospondylan *Wintonotitan wattsi*, imply a narrower-gauge stance and gait than that seen in *Diamantinasaurus* and *Savannasaurus* (Hocknull et al., 2009; Poropat et al., 2015a), suggesting that it was not the AODF 904.S1 trackmaker. Although we concede that the AODF 904.S1 trackmaker might be an as yet unidentified sauropod taxon, on the balance of the available evidence we consider *Diamantinasaurus matildae* and *Savannasaurus elliottorum* to be plausible candidates.

**Behavioural implications.** The effectively parallel sauropod trackways AODF 904.S1 and AODF 904.S2, the numerous tracks not attributed to trackways in the southeast of the site, and a short trackway in the northeast (AODF 904.S4), indicate that at least four sauropods traversed the Snake Creek Tracksite in the same direction within a very short timeframe. This is supported by the similar deformation features identified in the individual tracks of each trackmaker, which indicate similar substrate conditions—particularly with respect to water content—at the time of track formation. This implies that at least some sauropods in the Winton Formation might have been gregarious, a behaviour implied by several sauropod body fossil deposits (Myers & Fiorillo, 2009; Salgado et al., 2012) and many sauropod trackways around the world (Barnes & Lockley, 1994; Castanera et al., 2011; García-Ortiz & Pérez-Lorente, 2014; Lockley, Meyer & Santos, 1994; Lockley et al., 2002; Lockley et al., 2012; Myers & Fiorillo, 2009; Ostrom, 1972), including several trackways from the Broome Sandstone of Western Australia (Thulborn, 2012). As noted above, the two main trackways (AODF 904.S1 and AODF 904.S2) were not made simultaneously (as they appear to cross over), but little time evidently separated their formation based on their similar quality of preservation.

The middle section of the Snake Creek Tracksite (Figs. 10B, 17) preserves several tracks that are herein designated AODF 904.S3. These indicate a medium-sized sauropod (average PL 570 mm (Table 1), hip height <2.3 m) performing a rather adroit 180° turn (Figs. 10B, 17), with no evidence of ‘off-tracking’ sensu Ishigaki & Matsumoto (2009). Trackways made by turning sauropods (recently reviewed by Lockley et al. (2021)) have been described from: the Lower Jurassic of China (Lockley & Matsukawa, 2009; Xing et al., 2016a); the Upper Jurassic of Morocco (Ishigaki & Matsumoto, 2009), Portugal (Meyer et al., 1994; Castanera et al., 2014), Switzerland (Lockley & Meyer, 2000: pp. 12, 169–171; Meyer, 1990; Meyer, 1993; Stevens, Ernst & Marty, 2016), and the USA (Lockley & Hunt, 1995: pp. 164–165; Goodell et al., 2021); the Lower Cretaceous of China (Xing et al., 2015a); the upper Lower Cretaceous of China (Xing et al., 2015b); the lower Upper Cretaceous of Croatia (Dalla Vecchia, 1994; Leghissa & Leonardi, 1990; Mezga & Bajraktarević, 1999); and the Upper Cretaceous of Spain (Vila et al., 2008). However, although some of these trackways record a 180° turn by their trackmaker, in most cases the degree of directional change is much more gradual than that evinced by AODF 904.S3, wherein the three preserved tracks imply that a 180° turn was achieved in just two strides (Fig. 10B). Some exceptions are sauropod Trackway no. 6 from the Zhaojue Tracksite of China (Xing et al., 2015a), and Trackway #33 from the Fumanya site of Spain (Vila et al., 2008: figure 7). The former records a >180° turn achieved in two strides (as in AODF 904.S3), albeit with ‘off-tracking’, whereas the latter records several turns, notably one that seems to be almost as adroit as that of AODF 904.S3 (three strides for a 180° turn). Given the Maastrichtian age of the Fumanya site, and the fact that no sauropods other than titanosaurs survived until the Maastrichtian (Upchurch, Barrett & Dodson, 2004), it is probable that that trackway was made by a titanosaur (Castanera et al., 2016; Vila et al., 2013). If, as we hypothesise, the Snake Creek Tracksite sauropod trackmaker was also a titanosaur—specifically, an early-branching one—then the AODF 904.S3 trackway supports the notion that titanosaurs were more agile than other sauropods (García et al., 2015; Klinkhamer et al., 2019; Wilson & Carrano, 1999), and implies that increased manoeuvrability appeared relatively early in their evolution.

The pes track in the AODF 904.S1 trackway that is continuous with what appears to be an earlier-formed sauropod track (AODF 904.S1.10; Fig. 16) and the manus track with the most exaggerated anterior displacement rim (AODF 904.S1.11; Fig. 17) are situated immediately southeast of the AODF 904.S3 turning trackway (Fig. 10B). We suggest that the AODF 904.S3 trackmaker left its tracks before the AODF 904.S1 trackmaker traversed this part of the site, and that the pes track associated with manus track AODF 904.S3.06 was obliterated by the pes track of AODF 904.S1.11. The enlarged displacement rim anterior to the manus track of AODF 904.S1.11 suggests that the sediment in this section of the site had been disturbed prior to the traverse of the trackmaker, presumably by the turning sauropod.

**Size and speed of the trackmakers.** Alexander (1976) considered pes track length in sauropods to be equivalent to one-quarter of hip height. Following this, the AODF 904.S1 trackmaker was 3.66 m tall at the hips. Salisbury et al. (2017) inferred that, in sauropod trackways, the glenoacetabular length was approximately equivalent to the hip height of the trackmaker. Following Lallensack et al. (2019), we calculated the glenoacetabular distance of the AODF 904.S1 trackmaker using the formula 3/4SL + MPDp, where SL = stride length and MPDp = manual-pedal print distance (the distance between the centre of a pes track and the centre of a manus track within an ipsilateral manus–pes couplet). For AODF 904.S1.07, MPDp was found to be 908 mm, whereas middle pes stride length (MPSL) to AODF 904.S1.09 was 3369 mm. Thus, following the formula provided by Lallensack et al. (2019), the glenoacetabular distance of the AODF 904.S1 trackmaker is 3.44 m—quite close to the 3.66 m estimate derived from Alexander’s (1976) formula.

By either of the measures outlined above, the AODF 904.S1 trackmaker was larger than any of the sauropods described from the Winton Formation so far. All three described taxa preserve forelimb elements, but only the holotype of *Diamantinasaurus matildae* preserves a nearly complete hind limb (Hocknull et al., 2009; Poropat et al., 2015b). Excluding the non-preserved pes, its height is approximately 2.35 m (femur 1.345 m, tibia 0.795 m, + 10% on each element for cartilage (Schwarz, Wings & Meyer, 2007)). Thus, the overall hip height estimate for *Diamantinasaurus* by Salisbury et al. (2017) of 2.8 m seems plausible. Salisbury et al. (2017) also estimated that the hip height of *Wintonotitan wattsi* was 3.15 m; however, no hind limb material is known for this taxon (Hocknull et al., 2009; Poropat et al., 2015a). This estimate was presumably based on the forelimb height, which (excluding the shoulder) was at least 2.46 m (humerus >0.92 m (incomplete proximally), ulna 0.91 m, metacarpal III 0.41 m, + 10% for cartilage). In *Diamantinasaurus*, the forelimb height (excluding the shoulder) is 2.396 m (humerus 1.07 m, ulna 0.70 m, metacarpal III 0.41 m, + 10% for cartilage), meaning that the forelimb: hind limb ratio (if we assume an overall hip height of 2.8 m) is 0.86. Assuming the same ratio for *Wintonotitan*, the hind limb height was at least 2.88 m, and 3.15 m does not seem unreasonable; however, given that *Wintonotitan* is a non-titanosaurian somphospondylan, its limbs might have had different relative proportions than those of the early-branching titanosaur *Diamantinasaurus*. The hind limb of *Savannasaurus* is represented only by an astragalus and a metatarsal (Poropat et al., 2016; Poropat et al., 2020), but based on the preserved forelimb elements, its forelimb height was somewhat greater than 2.4 m (humerus >1.03 m, radius 0.77 m, metacarpal III 0.38 m, + 10% for cartilage), meaning its hind limb height (assuming a forelimb: hind limb of 0.86) was slightly greater than 2.8 m. Although specimens pertaining to larger-bodied sauropod individuals are known from the ‘upper’ Winton Formation near both Winton (S. F. Poropat and S. L. Rigby, 2021, personal observation) and Eromanga (Anonymous, 2007; Hocknull et al., 2019), these remain undescribed.

Following the formula *λ*/*h* (wherein *λ* = stride length and *h* = hip height) presented by Alexander (1976), the relative stride length of the AODF 904.S1 trackmaker was somewhere between 0.94 (with estimated hip height equal to four times average pes track length) and 0.98 (with estimated hip height based on glenoacetabular distance–hip height equivalence), implying movement at a walking pace. If we assume a hip height of 3.66 m, the speed was ~1.34 m s^−1^ = 4.83 km h^−1^. Stride length, and likely speed, is quite consistent along much of the AODF 904.S1 trackway (Table 1), although a slight increase in the rate of progression is evident at the end of the trackway (e.g. AODF 904.S1.19–22), since some of the strides exceed 4 m in length.

**Medium-sized theropod track.**

Ichnogen. et ichnosp. indet.

**Figure 19:**
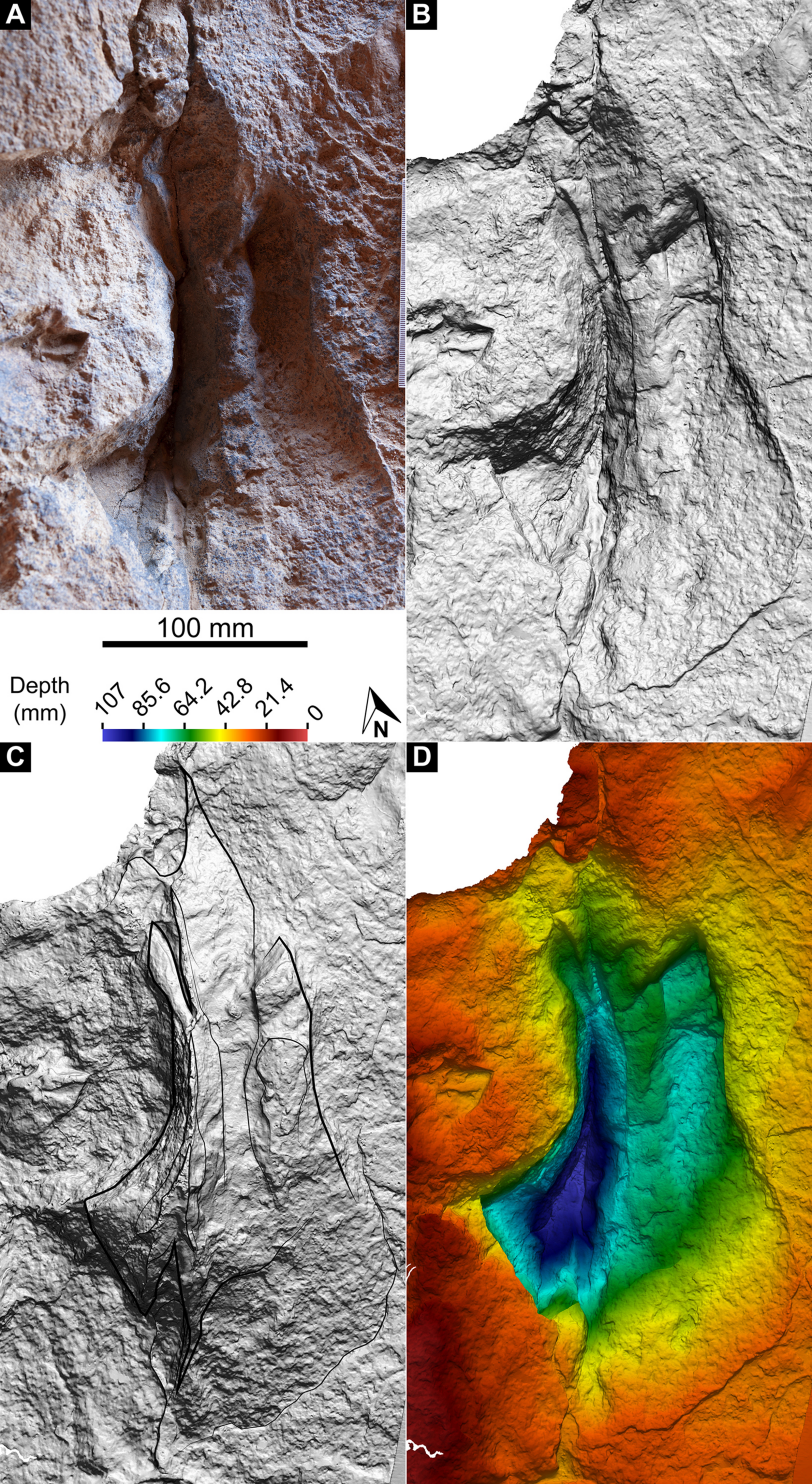
Theropoda ichnogen. et ichnosp. indet. penetrative track within sauropod pes track AODF 904.S1.23. (A) Photograph, lit from the east (right); (B) monochrome surface model, lit from the northwest (top left); (C) outline drawing on monochrome surface model, lit from the southwest (bottom left); (D) colour depth map.

**Referred track.** A single track (Fig. 19), situated anterolaterally within the sauropod pes track AODF 904.S1.23 in the northwest of the Tracksite, with its long axis running approximately south–north.

**Horizon and locality.** ‘Upper’ Winton Formation, Cenomanian–?lowermost Turonian (Tucker et al., 2013); Snake Creek Tracksite (AODL 251), Karoola Station, NW of Winton, Queensland, Australia.

**Description.** The track described herein as being from a medium-sized theropod (Fig. 19) is situated within a poorly-defined and weakly impressed sauropod pes track (AODF 904.S1.23) in the northwest of the Snake Creek Tracksite (Fig. 10). This track is anteroposteriorly elongate (270 mm) and mediolaterally narrow (130 mm), with impressions evidently corresponding to three digits. If we assume that the track corresponds to a right pes, and that the trackmaker was progressing from south to north, then the medial two digit impressions are substantially deeper than the lateral one. However, this might have been exaggerated by post-formational deformation: the sediment displaced by a sauropod manus track (AODF 904.S1.23) immediately west of the theropod track appears to have mediolaterally compressed, vertically deepened, and caused the coalescence of, the two medial digit impressions of the theropod track. Anteriorly, the digit impressions taper to sharp points, with the median digit forming a shallow, elongate furrow. The posterior margin of the medial digit impression terminates at a steep incline (possibly exacerbated by the sauropod manus track (AODF 904.S1.23)), whereas the median digit is extended posteriorly as a narrow but elongate furrow, mirroring the anterior projection of the same. The lateral digit impression, which is fusiform and shallowly impressed, fades out posterolaterally.

The only theropods heretofore reported from the Winton Formation are megaraptorids, notably *Australovenator wintonensis* (Hocknull et al., 2009; White et al., 2012; White et al., 2013; White et al., 2015a; White et al., 2015b; White et al., 2016; White et al., 2020). The type specimen of *Australovenator* preserves elements pertaining to both pedes, such that a composite pes can be assembled from left and right elements (Hocknull et al., 2009; Poropat et al., 2019b: Table S2; White et al., 2013; White et al., 2016). The dimensions of the pes of *Australovenator* appear to be slightly greater than those expected for the maker of the theropod track described herein. However, the distortion to which the track was subjected might have diminished its size, and/or the track might have been made by an immature *Australovenator*.

The elongate morphology of this track, coupled with the absence of a heel impression, implies that it is either a swim track (Milner & Lockley, 2016) or, more likely (as proposed by one of the reviewers of this manuscript (J. Lallensack)), a penetrative track (Gatesy & Falkingham, 2020). As described above, this theropod track appears to have been deformed by a sauropod manus track (AODF 904.S1.23), and occupies a position that corresponds to the anterolateral margin of the weakly impressed corresponding sauropod pes track. This implies that the theropod traversed the Tracksite (from south to north) before the AODF 904.S1 trackmaker, which would be congruent with a penetrative track formed when the substrate was less consistent. We tentatively interpret this track as that of a theropod, somewhat smaller than the *Australovenator wintonensis* type individual (which is ~5 m long).

**Small tridactyl tracks.**

Most of the non-sauropod tetrapod tracks preserved at the Snake Creek Tracksite are tridactyl. The authors originally thought that most of these were made by small-bodied theropods and were referable to *Skartopus australis*. However, one of the reviewers of this manuscript (M. G. Lockley) suggested that some, particularly the trackways now designated AODF 904.C1 and AODF 904.C2, were made by crocodyliforms or turtles locomoting underwater, and were very similar to tracks from elsewhere that have been designated as *Hatcherichnus*. Comparison of these tracks with published descriptions of fossilised crocodyliform underwater walking trackways (and those of extant crocodylians) suggests that the reviewer is correct; consequently, we have described these tracks as crocodyliform below.

In general, the trackways assigned herein to crocodyliforms are quite wide-gauge, with low pace angulation, and are comprised of tracks that are ectaxonic (although some tracks appear mesaxonic; Figs. 11–12). By contrast, the trackways assigned to small-bodied dinosaurs are narrow-gauge, with high pace angulation, and are comprised of mesaxonic tracks (Fig. 11). The theropod tracks are longer than wide and have sharp-tipped digit impressions, whereas the ornithopod tracks are wider than long and have blunter digit impressions (Thulborn & Wade, 1984). In instances where isolated tracks could not be attributed to a trackway, it was not always clear to which category they belonged; some could feasibly have been assigned to several. Generally, these isolated tracks have not been given more specific designations, nor have they been considered in detail herein. However, several of these tracks are morphologically congruent with turtle tracks, and are described as such.

**Theropod tracks.**

*Skartopus australis* Thulborn & Wade, 1984

**Holotype.** QM F10330—a single left track from Lark Quarry.

**Figure 20:**
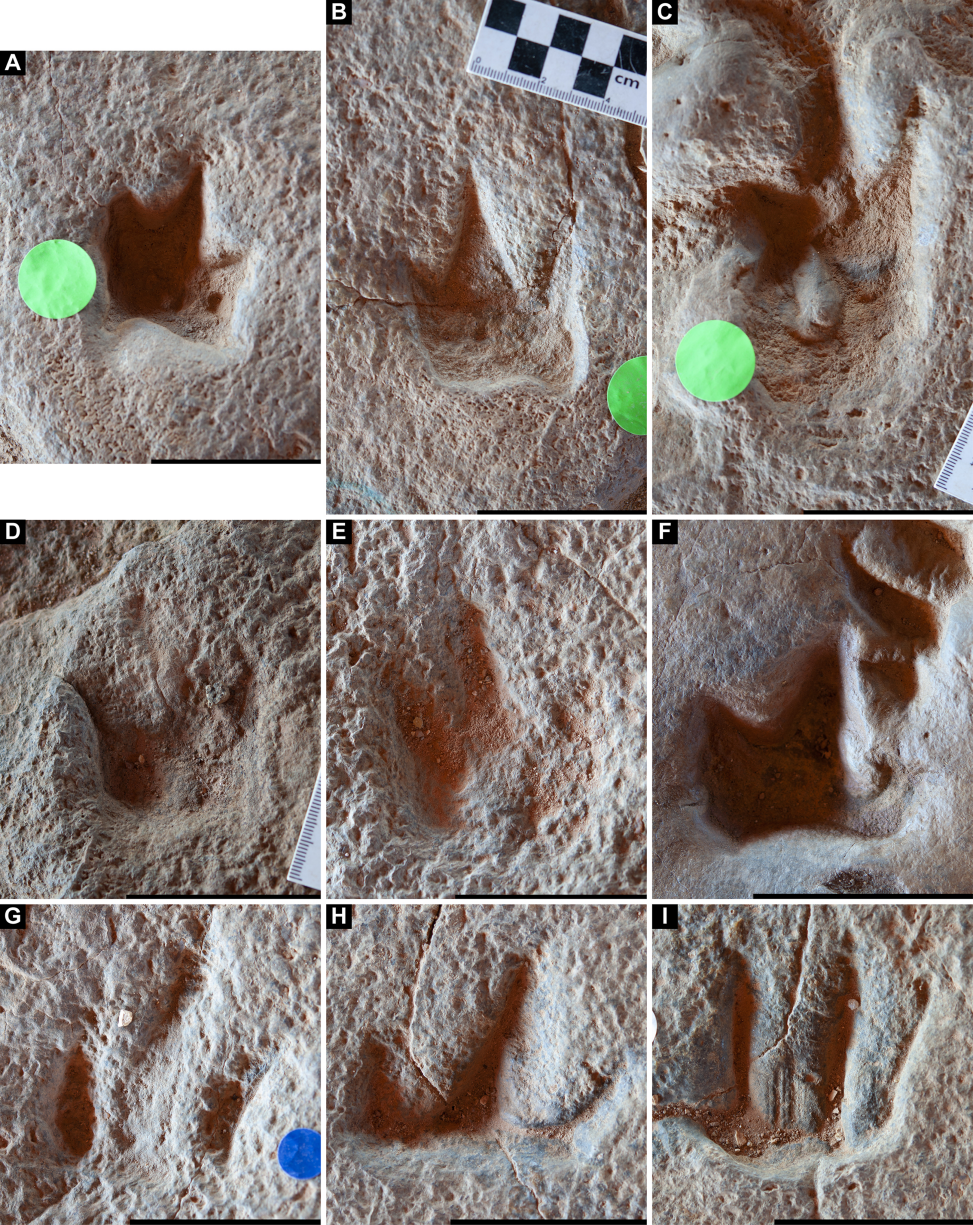
*Skartopus australis* and ?*Wintonopus latomorum* referred tracks. (A–C) Photographs of the pes tracks from the referred *Skartopus australis* trackway AODF 904.T1. All photographs are illuminated from the east (page left): (A) Left pes track AODF 904.T1.01; (B) Right pes track AODF 904.T1.02; and (C) Left pes track AODF 904.T1.03. (D–F) Photographs of the referred *Skartopus australis* tracks, possibly made by the same trackmaker as AODF 904.T1. All photographs are illuminated from the east (page left): (D) Right pes track; (E) Left pes track; and (F) Left pes track, evidently overprinted by a crocodyliform track (*Hatcherichnus*: AODF 904.C3.06). (G–I) Photographs of the pes tracks from the referred ?*Wintonopus latomorum* possible trackway AODF 904.O1. All photographs are illuminated from the east (page left): (G) Right pes track AODF 904.O1.02; (H) Left pes track AODF 904.O1.03; and (I) Right pes track AODF 904.O1.04. Scale bars = 50 mm.

**Figure 21:**
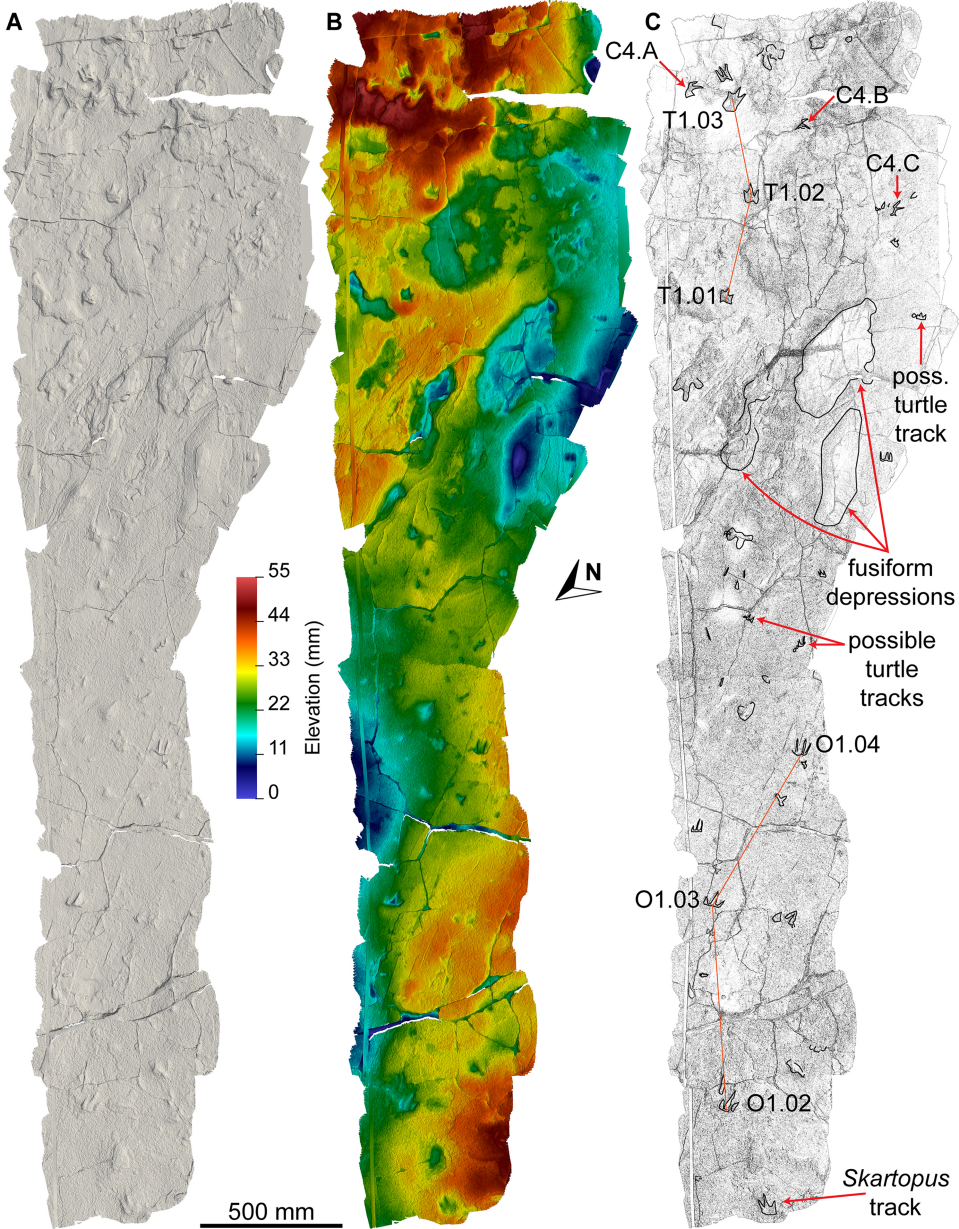
*Skartopus australis* referred trackway AODF 904.T1 and ?*Wintonopus latomorum* referred ?trackway AODF 904.O1, along with additional tridactyl tracks. (A) Monochrome surface model, lit from the southeast (top); (B) colour depth map; (C) outline drawing showing the tracks in the AODF 904.T1 and AODF 904.O1 trackways, along with other tridactyl tracks from this section of the site.

**Referred tracks.** AODF 904.T1, a short trackway wherein the heels of the tracks are particularly well-defined (Figs. 11, 20A–20C & 21); and several other abbreviate trackways and isolated tracks (Figs. 11, 20D–20F & 21).

**Horizon and locality.** ‘Upper’ Winton Formation, Cenomanian–?lowermost Turonian (Tucker et al., 2013); Snake Creek Tracksite (AODL 251), Karoola Station, NW of Winton, Queensland, Australia.

**Description.** The small theropod tracks from the Snake Creek Tracksite, especially those designated as AODF 904.T1, conform with the morphology of the *Skartopus australis* tracks from Lark Quarry and Seymour Quarry (Thulborn & Wade, 1984). The tracks are tridactyl, and appear to have been made by a digitigrade biped. In the AODF 904.T1 tracks, the impression of digit II (or possibly a weak impression of a reduced and non-weight-bearing digit I) extends further posteriorly than that of digit IV (Figs. 20A–20B). These latter two tracks suggest that one of the Lark Quarry *Skartopus* tracks, depicted by Thulborn & Wade (1984: pl. 15, fig. a) as a “?right footprint”, is actually a left track: the shape of its posterior margin is a mirror image of that of AODF 904.T1.02 (a right track) from the Snake Creek Tracksite.

Several additional tracks in the central and southeast sections of the Tracksite are similar to those assigned to AODF 904.T1, and all could conceivably belong to the same trackway. However, large gaps that lack tracks altogether or only preserve different track morphotypes, exist between these tracks. If we assume that they were made by the same trackmaker, then two of these tracks were formed prior to those in the AODF 904.T1 trackway (Figs. 20D–20E), whereas another was formed subsequently (Fig. 20F), based on their positions and the direction of the trackmaker’s movement (Fig. 11: tracks labelled “*Skartopus* track”).

**Ornithopod tracks.**

*Wintonopus latomorum* Thulborn & Wade, 1984

**Holotype.** QM F10319—a single right track from Lark Quarry.

**Figure 22:**
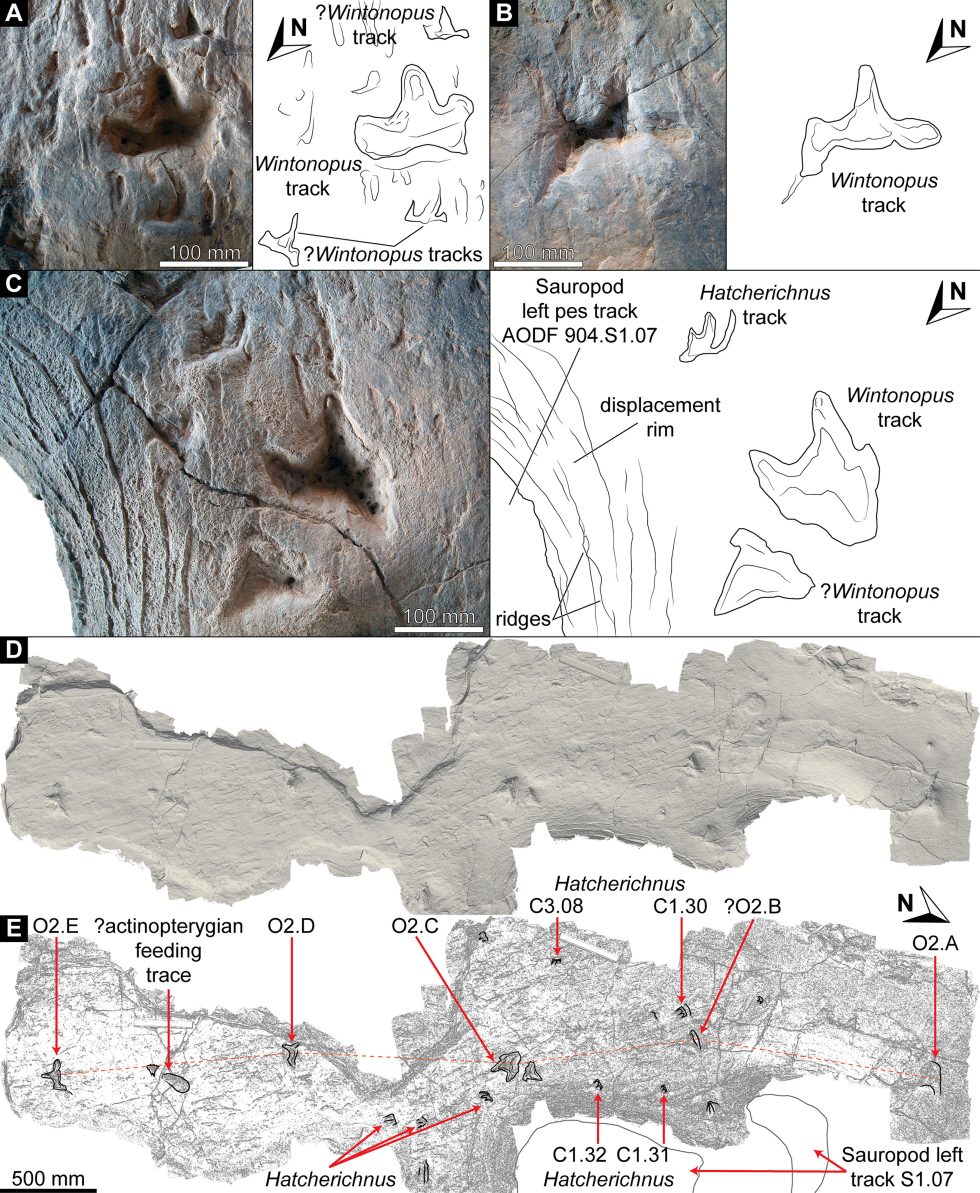
*Wintonopus latomorum* referred tracks. (A) Photograph of a large, isolated *Wintonopus latomorum* ?right pes track, surrounded by at least three other tridactyl tracks (?*Wintonopus*), illuminated from the east (page top left); (B) Photograph of *Wintonopus latomorum* ?left pes track AODF 904.O2.E, illuminated from the east (page top left); (C) Photograph of *Wintonopus latomorum* ?left pes track AODF 904.O2.C, associated with an isolated *Hatcherichnus* pes track and the sauropod left pes track AODF 904.S1.07, illuminated from the east (page top left). (D) Monochrome surface model of the *Wintonopus* possible trackway AODF 904.O2, lit from the southwest (top). (E) Outline drawing showing the tracks in the AODF 904.O2 trackway, along with other tracks and traces from this section of the site.

**Referred tracks.** AODF 904.O1—a possible trackway comprising three consecutive tracks (Figs. 11, 20G–20I, 21); AODF 904.O2—a possible trackway comprising five tracks (Figs. 11, 22B–22E); and several isolated tracks (Fig. 21A).

**Horizon and locality.** ‘Upper’ Winton Formation, Cenomanian–?lowermost Turonian (Tucker et al., 2013); Snake Creek Tracksite (AODL 251), Karoola Station, NW of Winton, Queensland, Australia.

**Description.** Both small (Figs. 20G–20I, 21) and large (Fig. 22) ornithopod tracks at the Snake Creek Tracksite conform with the original diagnosis of *Wintonopus latomorum* presented by Thulborn & Wade (1984). The two best preserved *Wintonopus* tracks at the Snake Creek Tracksite (Figs. 22A, 22C) have Size Index values (SI; sensu Thulborn & Wade (1984)) of 123.9 mm (120 mm long × 128 mm wide) and 129 mm (128 mm long × 130 mm wide: AODF 904.O2.C) respectively, whereas the broadest *Wintonopus* track at the Snake Creek Tracksite (AODF 904.O2.E: Fig. 22B) has a SI of 127.1 mm (101 mm long × 160 mm wide). Thus, these *Wintonopus* tracks are larger than all but the single largest set of *Wintonopus* tracks (SI = 266 mm) at Lark Quarry (Thulborn & Wade, 1984). If we follow Alexander (1976) and assume that hip height was four times pes length, the largest ornithopod trackmakers at the Snake Creek Tracksite ranged from 404–512 mm tall at the hips.

The *Wintonopus* tracks are tridactyl, wider mediolaterally than long anteroposteriorly, and are asymmetrical, giving the impression that they were rotated inward relative to the direction of travel. The digit impressions are generally mediolaterally broad and have blunt, rounded tips, although in one track (AODF 904.O2.E: Fig. 22B) the middle toe impression is somewhat more tapered anteriorly; Thulborn & Wade (1984) noted that some *Wintonopus* tracks at Lark Quarry show elongate digit III impressions, which they interpreted as having been extended via scrape-marks. In the ornithopod tracks from the Snake Creek Tracksite, no impressions of phalangeal pads were identified, digit III is longest, and digit II is shortest, and the interdigital angle between digits II and III is invariably higher than that between digits III and IV. The posterior margin of the track is concave forwards; in one track (AODF 904.O2.E: Fig. 22B) the digit impressions interrupt the posterior margin.

Almost all of the possible ornithopod tracks at the Snake Creek Tracksite can be arranged into trackways. The first trackway comprises three tracks (AODF 904.O1.02–04; Figs. 20G–20I), preserved northwest of the *Skartopus* trackway described above (AODF 904.T1; Figs. 11, 21). Although one of these tracks (AODF 904.O1.03: Fig. 20H) appears to relatively closely match some of the *Wintonopus* tracks from Lark Quarry (Thulborn & Wade, 1984), the morphology of the other two tracks (AODF 904.O1.02: Fig. 20G; and AODF 904.O1.04: Fig. 20I) is markedly divergent. Although it is not inconceivable that this trackway pertains to something other than an ornithopod, we note that in this apparent trackway, the trackway width is relatively lower, and the pace angulation relatively higher, than in the crocodyliform and possible turtle trackways described below. Differentiating them from the tracks assigned to *Skartopus australis* is more difficult—their short anteroposterior dimension and relative breadth might indicate a similar trackmaker, albeit one leaving only impressions of the tips of its digits. The other potential trackway (AODF 904.O2: Figs. 11, 22) comprises several poorly preserved tracks (AODF 904.O2.A–B, D) and two well-preserved tracks (AODF 904.O2.C (Fig. 22C) and AODF 904.O2.E (Fig. 22B)), all of which are easily differentiated from the *Skartopus* tracks described herein.

**Crocodyliform tracks.**

*Hatcherichnus isp*.

**Figure 23:**
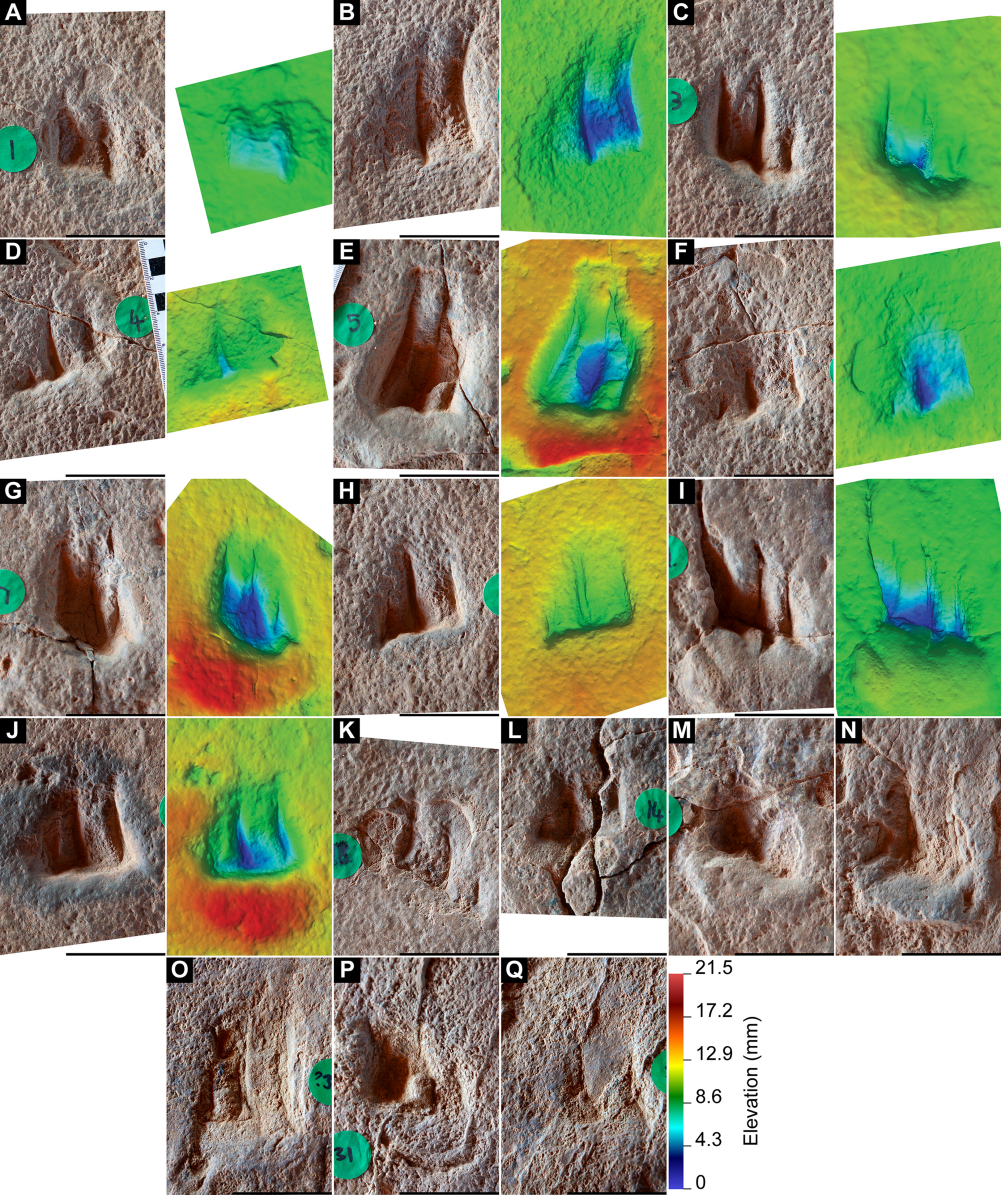
*Hatcherichnus* isp. referred tracks. (A–Q) *Hatcherichnus* isp. pes tracks from the referred trackway AODF 904.C1. Photographs (left) and colour depth maps (right) are presented for tracks AODF 904.C1.01–10, whereas for tracks AODF 904.C1. 13–15, 22, and 30–32, only photographs are included. All photographs of AODF 904.C1 tracks are illuminated from the southeast (page left): (A) Left pes track AODF 904.C1.01; (B) Right pes track AODF 904.C1.02; (C) Left pes track AODF 904.C1.03; (D) Right pes track AODF 904.C1.04; (E) Left pes track AODF 904.C1.05; (F) Right pes track AODF 904.C1.06; (G) Left pes track AODF 904.C1.07; (H) Right pes track AODF 904.C1.08; (I) Left pes track AODF 904.C1.09; (J) Right pes track AODF 904.C1.10; (K) Left pes track AODF 904.C1.13; (L) Right pes track AODF 904.C1.14; (M) Left pes track AODF 904.C1.15; (N) Right pes track AODF 904.C1.22; (O) Right pes track AODF 904.C1.30; (P) Left pes track AODF 904.C1.31; and (Q) Right pes track AODF 904.C1.32. Scale bars = 50 mm.

**Figure 24:**
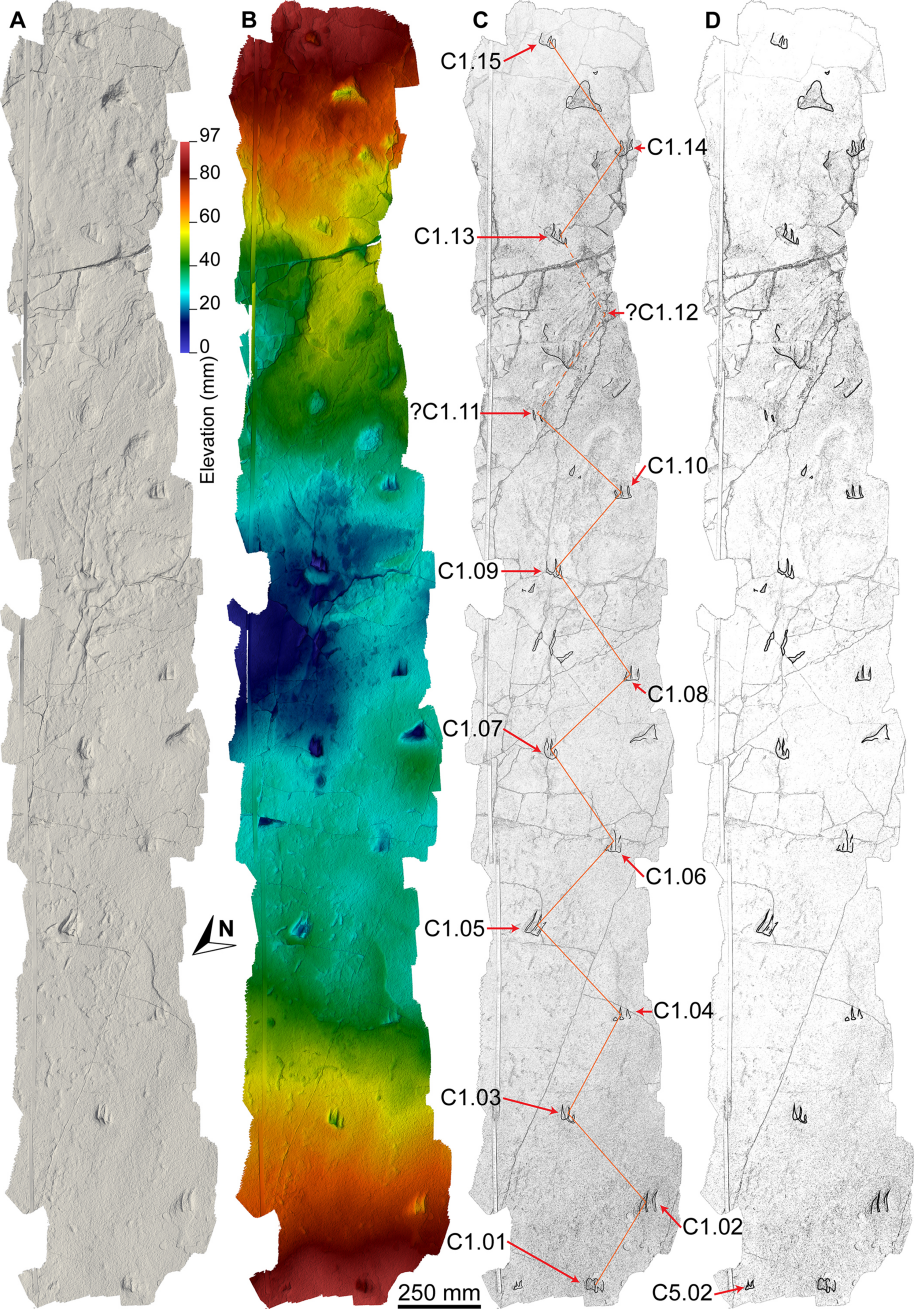
*Hatcherichnus* isp. referred trackway AODF 904.C1. (A) Monochrome surface model, lit from the southeast (top); (B) colour depth map; (C) outline drawing showing the sequence of fifteen tracks (two of which are inferred (AODF 904.C1.11 and AODF 904.C1.12)); (D) outline drawing showing the tracks in the AODF 904.C1 trackway, along with other tridactyl tracks from this section of the site.

**Figure 25:**
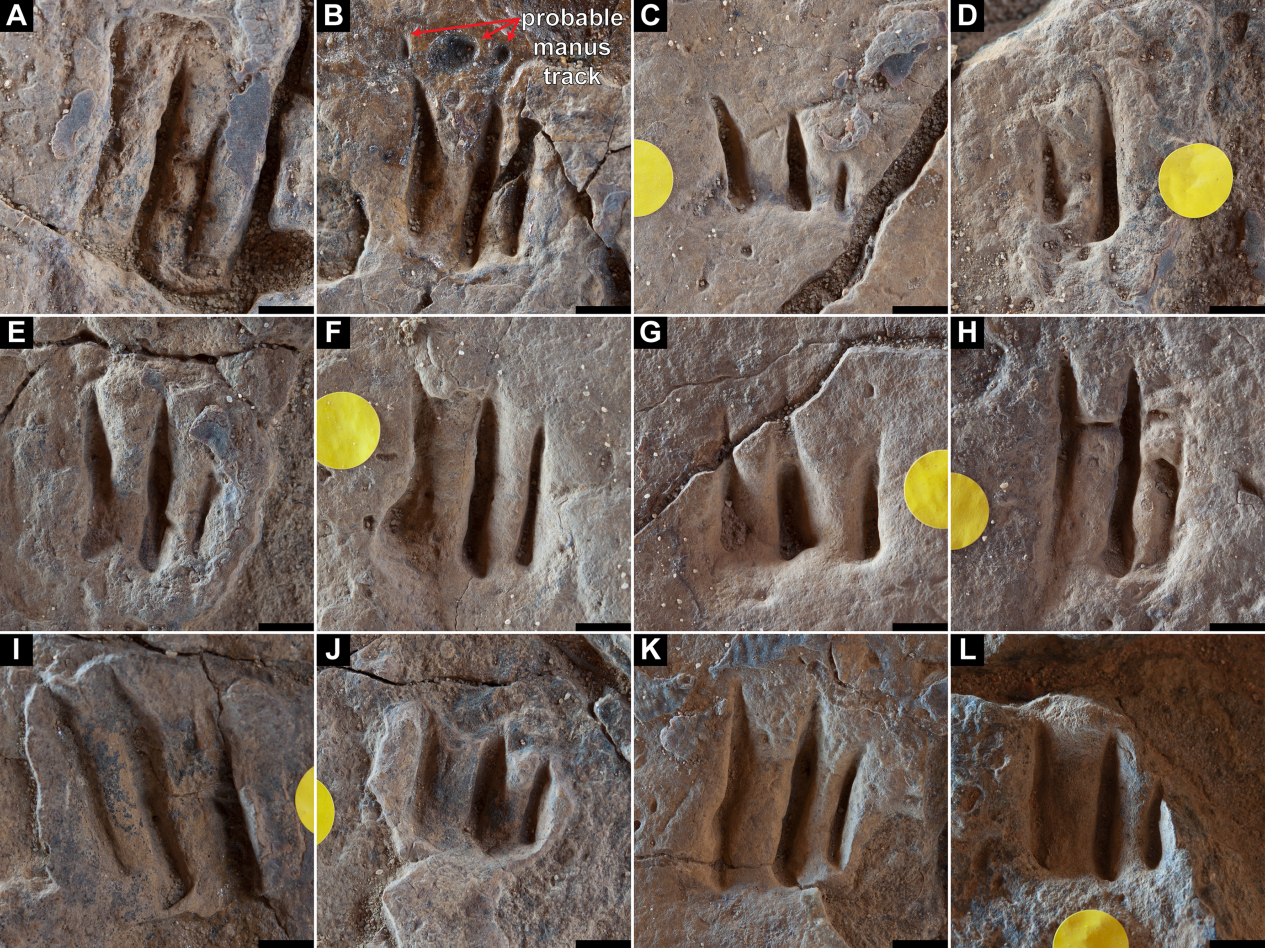
*Hatcherichnus* isp. referred tracks. (A–L) Photographs of the *Hatcherichnus* isp. pes tracks from the referred trackway AODF 904.C2. All photographs are illuminated from the southeast (page left): (A) left pes track AODF 904.C2.01; (B) left pes track AODF 904.C2.03; (C) left pes track AODF 904.C2.05; (D) right pes track AODF 904.C2.06; (E) left pes track AODF 904.C2.07; (F) left pes track AODF 904.C2.09; (G) right pes track AODF 904.C2.10; (H) left pes track AODF 904.C2.11; (I) right pes track AODF 904.C2.12; (J) left pes track AODF 904.C2.13; (K) left pes track AODF 904.C2.15; and (L) left pes track AODF 904.C2.17. Scale bars = 10 mm.

**Figure 26:**
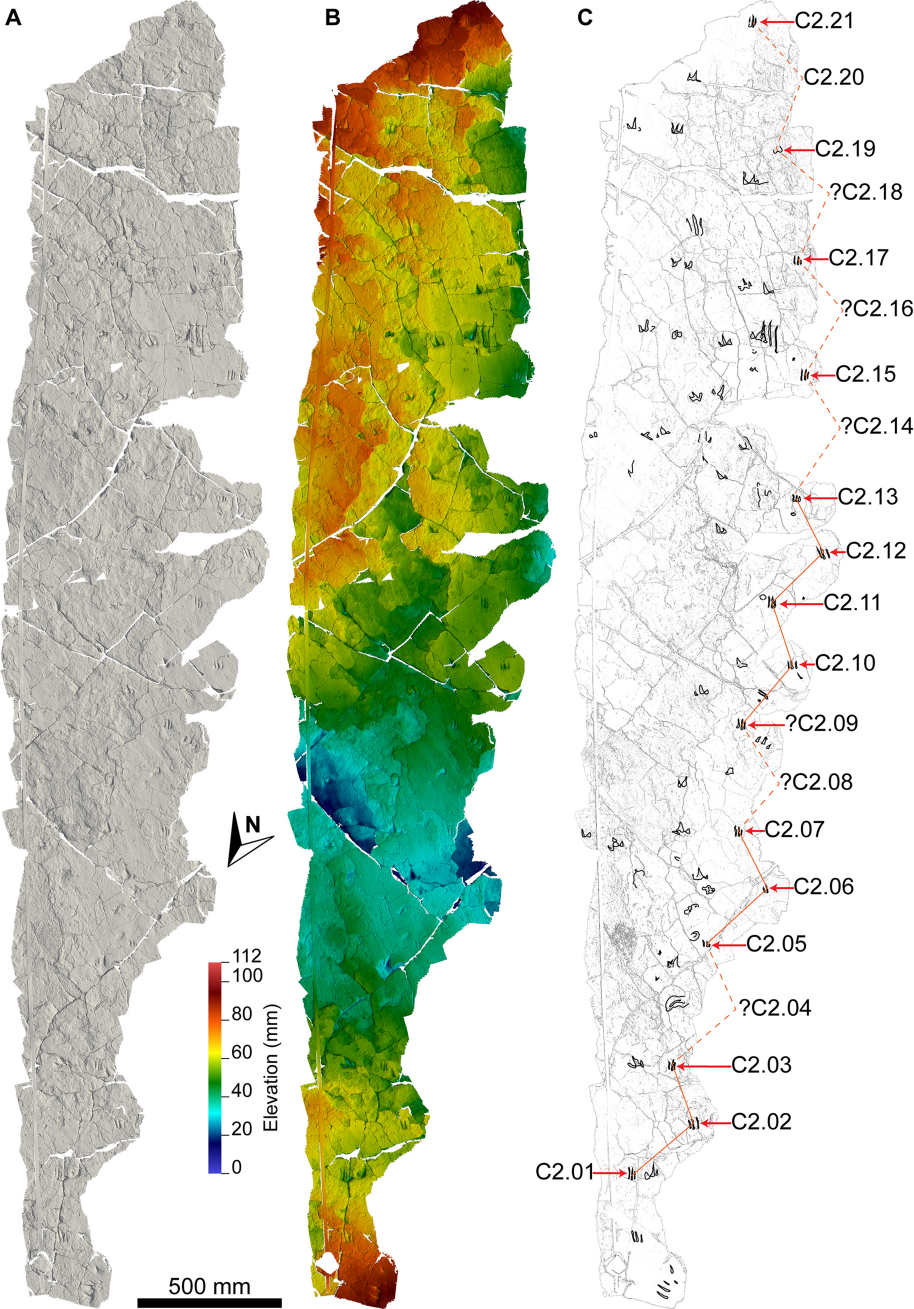
*Hatcherichnus* isp. referred trackway AODF 904.C2, along with additional tridactyl tracks. (A) Monochrome surface model, lit from the southeast (top); (B) colour depth map; (C) outline drawing showing the tracks in the AODF 904.C2 trackway, along with other tridactyl tracks from this section of the site.

**Figure 27:**
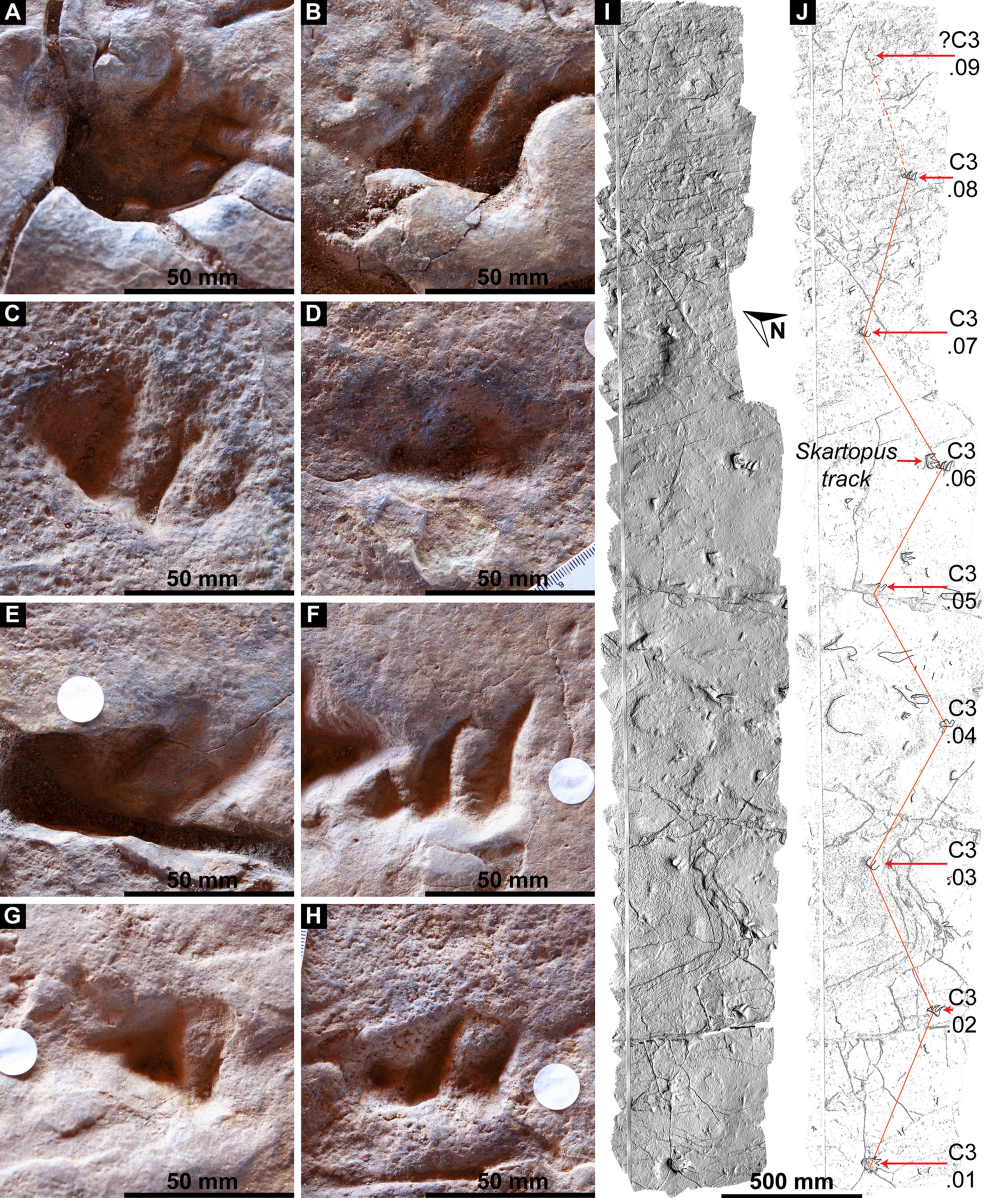
*Hatcherichnus* isp. referred trackway AODF 904.C3. (A–H) Photographs of the *Hatcherichnus* isp. pes tracks from the referred trackway AODF 904.C3. All photographs are illuminated from east (page top): (A) Left pes track AODF 904.C3.01; (B) Right pes track AODF 904.C3.02; (C) Left pes track AODF 904.C3.03; (D) Right pes track AODF 904.C3.04; (E) Indistinct left pes track AODF 904.C3.05; (F) Right pes track AODF 904.C3.06, almost overprinting the impression of digit III of a theropod track assigned to *Skartopus australis*; (G) Left pes track AODF 904.C3.07; and (H) Right pes track AODF 904.C3.08. (I) Monochrome surface model of the AODF 904.C3 trackway, illuminated from the northwest (bottom left); (J) Outline drawing showing the tracks in the AODF 904.C3 trackway, along with a few other tracks from this section of the site.

**Figure 28:**
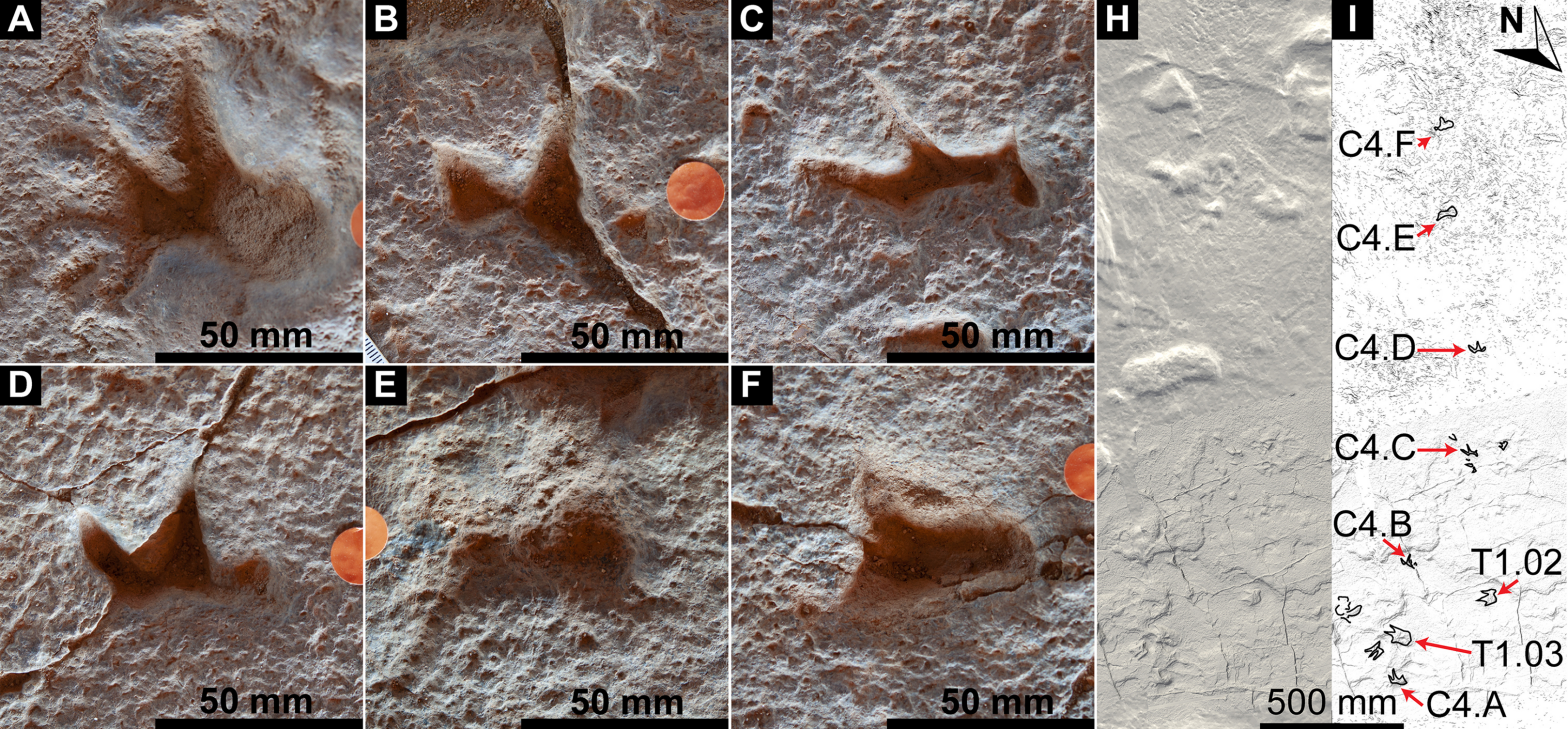
*Hatcherichnus* isp. referred possible trackway AODF 904.C4. (A–F) Photographs of the *Hatcherichnus* isp. pes tracks from the possible trackway AODF 904.C4. All photographs are illuminated from east (page bottom): (A) ?Pes track AODF 904.C4.A; (B) ?Pes track AODF 904.C4.B; (C) ?Pes track AODF 904.C4.C; (D) ?Pes track AODF 904.C4.D; (E) ?Pes track AODF 904.C4.E; (F) ?Pes track AODF 904.C4.F. (G) Monochrome surface model of the AODF 904.C4 possible trackway. (H) Outline drawing showing the AODF 904.C4 possible trackway and other tracks from this section of the site.

**Figure 29:**
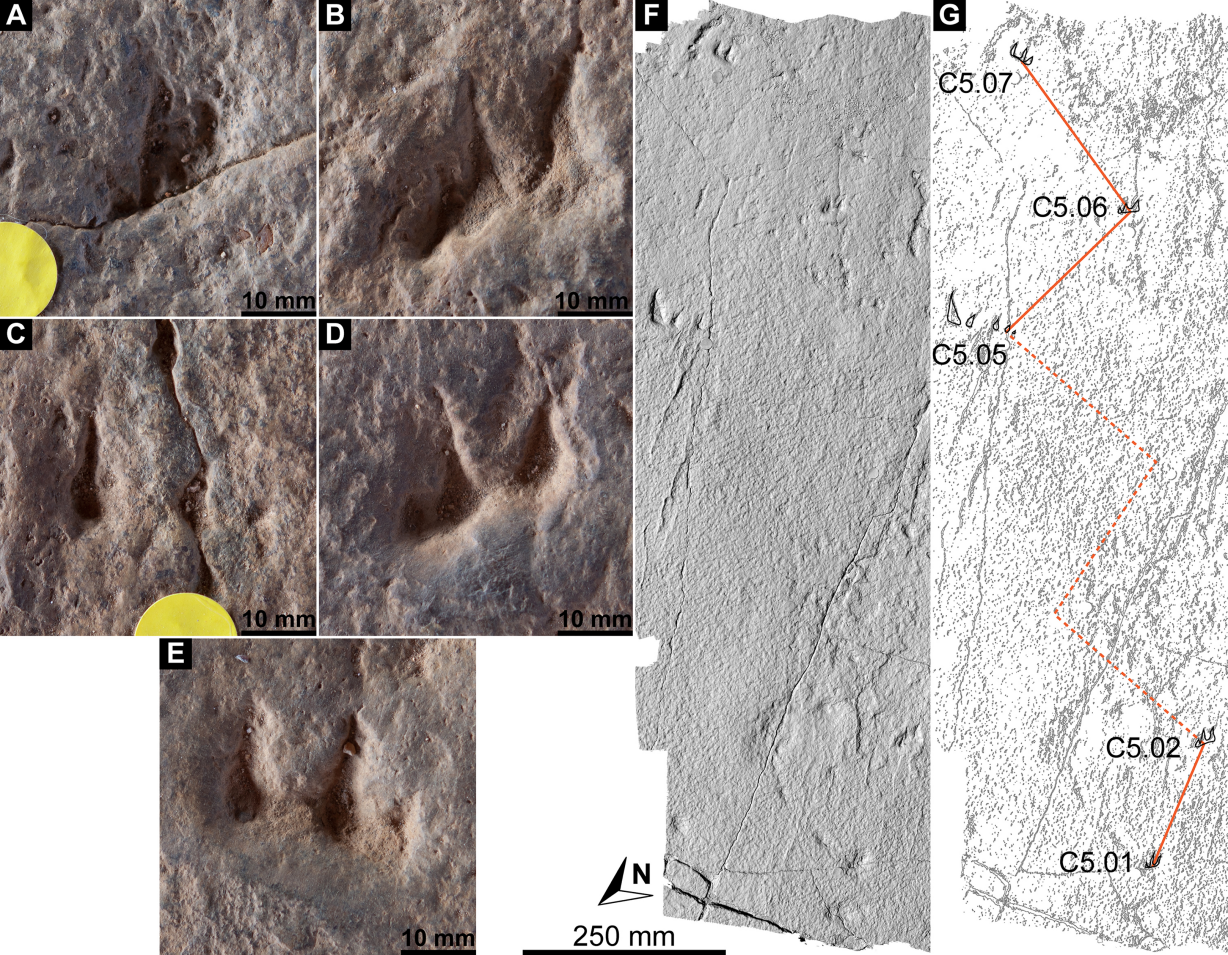
*Hatcherichnus* isp. referred trackway AODF 904.C5. (A) Left pes track AODF 904.C5.01, illuminated from the east (left); (B) Right pes track AODF 904.C5.02, illuminated from the east (left); (C) Left pes track AODF 904.C5.05, illuminated from the east (left); (D) Right pes track AODF 904.C5.06, illuminated from the east (left); (E) Left pes track AODF 904.C5.07, illuminated from the east (left). (F) Monochrome surface model of the AODF 904.C5 trackway. (G) Outline drawing showing the tracks in the AODF 904.C5 trackway.

**Referred tracks.** AODF 904.C1—a northwest–southeast directed trackway that would have comprised at least 37 paces when complete (Figs. 11, 23–24; Table 2), but of which only the following tracks are preserved: ten consecutive alternating tracks (AODF 904.C1.1–10), a gap equivalent to two consecutive tracks, three consecutive tracks (AODF 904.C1.13–15), a gap equivalent to six consecutive tracks, a single right track (AODF 904.C1.22), a gap equivalent to seven consecutive tracks, three consecutive alternating tracks (AODF 904.C1.30–32), and three consecutive left tracks without corresponding right tracks (AODF 904.C1.33, 35, 37); AODF 904.C2—a north–south directed trackway comprising eleven (possibly twelve) consecutive left tracks and four of the corresponding ten right tracks, many of which comprise three parallel scratch marks (Figs. 12, 25–26; Table 2); AODF 904.C3—a southwest–northeast trending trackway preserving eight (possibly nine) consecutive alternating tracks (Figs. 11, 27; Table 2); AODF 904.C4—a southwest–northeast trending possible trackway preserving six tracks (Figs. 11, 28); AODF 904.C5—a northwest–southeast directed trackway comprising two consecutive alternating tracks, a gap equivalent to two consecutive tracks, and three consecutive tracks (Figs. 11, 29); and other isolated tracks or tentative trackways (Figs. 11–12).

**Table 2:** Snake Creek Tracksite crocodyliform track measurements (all in mm).

**AODF 904.C1**	**PL to I**	**PL to II**	**PL to III**	**PW**	**SI (III)**	**TW**	**TF–TT**	**PP III–PP III**	**PP III–PP III**	**TF–TT**	**PM II–PM II**
1	–	43	–	51	–	–	1–3	495	–	1–2	255
2	–	56	62	50	56	260	2–4	–	580	2–3	335
3	–	63	50	46	48	260	3–5	580	–	3–4	333
4	–	40	41	48	44.5	270	4–6	–	512	4–5	347
5	–	69	68	49	58.5	305	5–7	538	–	5–6	335
6	–	–	40	47	43.5	265	6–8	–	519	6–7	318
7	–	60	59	42	50.5	270	7–9	545	–	7–8	315
8	–	52	40	47	43.5	285	8–10	–	544	8–9	357
9	–	59	54	52	53	275	9–11	470	–	9–10	299
10	–	50	50	50	50	280	10–12	–	502	10–11	–
13	–	37	38	46	42	–	13–15	568	–	13–14	–
14	–	41	38	47	42.5	270	–	–	–	14–15	308
15	–	33	32	48	40	–	–	–	–	–	350
Average	–	50.3	47.7	47.9	47.8	274	Average	532.7	531.4	Average	322.9
**AODF 904.C2**	**PL to I**	**PL to II**	**PL to III**	**PW**	**SI (III)**	**TW**	**TF–TT**	**PP III–PP III**	**PP III–PP III**	**TF–TT**	**PP III–PP III**
‘0’	40	–	58	32	45	–	‘0’–1	210	–	–	–
1	40	–	42	30	36	–	1–3	408	–	–	–
3	26	–	30	20	25	–	3–5	448	–	–	–
5	10	–	22	22	22	–	5–7	396	–	5–6	274
6	–	18	30	15	22.5	170	–	–	–	6–7	220
7	21	–	32	25	28.5	–	7–9	371	–	–	–
9	29	–	38	28	33	–	9–11	440	–	9–10	276
10	25	32	24	29	26.5	150	10–12	–	397	10–11	222
11	24	–	38	24	31	155	11–13	374	–	11–12	250
12	34	45	35	29	32	170	12–14	–	440	12–13	222
13	18*	–	21*	25	23	~151	13–15	420	–	13–14	~280
14	19	–	–	–	–	>140	–	–	–	14–15	215
15	24	–	39	27	33	–	15–17	400	–	–	–
17	18*	–	27	25	26	–	–	–	–	–	–
21	24	–	41	22	31.5	–	–	–	–	–	–
Average	25.1	31.7	34.1	25.2	29.6	156	Average	385.2	418.5	Average	244.9
**AODF 904.C3**	**PL to I**	**PL to II**	**PL to III**	**PW**	**SI (III)**	**TW**	**TF–TT**	**PP III–PP III**	**PP III–PP III**	**TF–TT**	**PP III–PP III**
1	40	–	54	78	66	–	1–3	1058	–	1–2	582
2	–	40	42	50	46	270	2–4	–	1016	2–3	580
3	–	31	38	58	48	341	3–5	980	–	3–4	570
4	–	31	25	46	35.5	310	4–6	–	940	4–5	555
5	–	45	47	50	48.5	300	5–7	950	–	5–6	523
6	18	35	42	50	46	318	6–8	–	1085	6–7	574
7	21	28	–	42	–	282	–	–	–	7-?9	614
8	17	30	32	41	26.5	–	–	–	–	–	–
Average	24	34.3	40	51.9	45.2	303.5	Average	996	1013.7	Average	571.1
**AODF 904.C5**	**PL to I**	**PL to II**	**PL to III**	**PW**	**SI (III)**	**TW**	**TF–TT**	**PP III–PP III**	**PP III–PP III**	**TF–TT**	**PP III–PP III**
1	6	17	13	16	14.5	–	–	–	–	1–2	145
2	12	21	25	22	23.5	–	–	–	–	–	–
5	4	13	17	27	22	–	5–7	300	–	5–6	191
6	7	20	20	24	22	145.6	–	–	–	6–7	210
7	9	20	19	27	23	–	–	–	–	–	–
Average	7.6	18.2	18.8	23.2	21	145.6	Average	300	–	Average	182

**Note:**

Abbreviations as follows: PL to #, pes length to tip of digit I, II, or III (anteroposterior); PW, pes width (mediolateral); SI (III), standard index (calculated by adding PL[III] and PW and halving the resultant value); TW, trackway width; TF–TT, track measured from–track measured to; PP III–PP III, stride length measured between posterior margins of pedal digit III in successive ipsilateral pes tracks; PM II–PM II = pace length measured between posteromedial margins of digit II in successive contralateral prints.

**Horizon and locality.** ‘Upper’ Winton Formation, Cenomanian–?lowermost Turonian (Tucker et al., 2013); Snake Creek Tracksite (AODL 251), Karoola Station, NW of Winton, Queensland, Australia.

**Description:** The crocodyliform trackways at the Snake Creek Tracksite generally comprise pes tracks only, and these tend to preserve evidence of only the innermost three digits (I–III). The few tracks in which only two digit impressions are evident (e.g., AODF 904.C2.06 [Figure 25D]) are thought to preserve only digits II and III. The digits are well-separated, and their impressions constitute anteriorly tapered grooves. Digit III is almost always longest, whereas digit I is invariably shortest; thus, the tracks are ectaxonic, and conform with the morphology of crocodyliform pedes, wherein digit length progressively increased from digit I to digit III; digit IV is approximately equivalent in length to digit II, but weakly-developed (Lockley et al., 2014). The interdigital angles between digits I and II, and digits II and III, are acute (≤30°). No evidence of phalangeal pads was identified. The pedal digit impressions in one of the smaller crocodyliform trackways (AODF 904.C2 (Figs. 25–26)) are more or less straight, whereas in one small (AODF 904.C5 (Fig. 29)) and both larger crocodyliform trackways (AODF 904.C1 (Figs. 23–24), and especially AODF 904.C3 (Fig. 27)), the digit impressions curve anterolaterally. This might have been caused by outward rotation as the trackmakers lifted their pedes off the substrate. The posterior margins of the pes tracks are flat or shallowly concave forward and are angled anterolaterally–posteromedially relative to the direction of travel. This gives the impression of distinct inward rotation, imparting the trackways a somewhat ‘pigeon-toed’ appearance. In several pes tracks, claw marks from all three toes intersect the posterior margin. The posterior margins of the tracks are seldom well-defined and are often interrupted by the digit impressions. Several of the tracks are surrounded, in whole or in part, by raised displacement rims; these are generally most prominent posterior to the track.

The only crocodyliform trackway at the Snake Creek Tracksite that appears to preserve any manus tracks is AODF 904.C2. The most obvious possible manus track is situated anterior to the pes track of AODF 904.C2.03 (Fig. 25B), and comprises three small indentations: a mediolaterally narrow, pinprick-like lateral impression; a broad, oval middle impression; and a small, circular medial impression. Single, small indentations can also be seen anterior to the pedal digit III impressions in AODF 904.C2.07 (Fig. 25E) and AODF 904.C2.10 (Fig. 25G). The near-absence of manus tracks, the absence of evidence of belly- or tail-dragging, and the similarity seen between the crocodyliform trackways at the Snake Creek Tracksite and swim tracks from around the world (Milner & Lockley, 2016; Milner, Lockley & Kirkland, 2006; Moreno, Blanco & Tomlinson, 2004; Whyte & Romano, 2001; Xing et al., 2013) indicates that these crocodyliforms were walking underwater, almost exclusively using their hind limbs.

The crocodyliform trackmakers appear to have been quite variable in size (Table 2). Average pes track width varied from 23.2 mm (in AODF 904.C5 [Figure 29]) to 51.9 mm (in AODF 904.C3 [Figure 27]), whereas external trackway width ranged from 145.6 mm (in AODF 904.C5) to 303.5 mm (in AODF 904.C3). Their similar level of preservation quality implies that multiple crocodyliform trackmakers traversed the Snake Creek Tracksite within a relatively short timeframe, at a time when the site was submerged, after the passage of the sauropods.

**Discussion.** Although crocodyliforms have a reasonable Cretaceous–Recent body fossil record in Australia (Willis, 2006; Ristevski et al., 2020), no fossil tracks pertaining to crocodyliforms have ever been reported from the continent (Lockley et al., 2010c). Whereas the Triassic record of crocodylomorph tracks is restricted to North America and Europe (Klein & Lucas, 2010), the Jurassic crocodyliform track record is more extensive (Lockley & Meyer, 2004), spanning North America (Foster & Lockley, 1997; Lockley & Foster, 2010; Lockley, Kirkland & Milner, 2004; Lockley et al., 2018b), Europe (Avanzini et al., 2007; Avanzini, Piñuela & García-Ramos, 2010; Avanzini et al., 2010; Castanera et al., 2021; Moreau et al., 2019; Romano & Whyte, 2010), Asia (Lockley et al., 2010b), Iran (Abbassi et al., 2015), and Africa (Klein et al., 2018; Olsen & Galton, 1984). The Early Cretaceous crocodyliform track record is similarly extensive, with records from North America (Houck et al., 2010; Kukihara & Lockley, 2012; Kukihara, Lockley & Houck, 2010; Lockley, 2010; Lockley, Kukihara & Mitchell, 2006; Lockley et al., 2010a; Lockley et al., 2014; McCrea et al., 2014), Europe (Fuentes Vidarte & Meijide Calvo, 2001; Pascual Arribas et al., 2005), Asia (Kim et al., 2020; Le Loeuff et al., 2010; Lockley et al., 2010b; Lockley et al., 2020), and Brazil (Campos, Da Silva & Milàn, 2010). By contrast, the Late Cretaceous crocodyliform track record is relatively limited, the only reports being from North America (Falkingham, Milàn & Manning, 2010; Lockley et al., 2018a; McCrea et al., 2014; Simpson et al., 2010), Europe (Segura et al., 2016; Vila et al., 2015), and Asia (Lee et al., 2019).

Among Mesozoic crocodylomorph/crocodyliform tracks, the crocodyliform tracks from the Snake Creek Tracksite most closely correspond with those assigned to *Hatcherichnus* (Foster & Lockley, 1997; Avanzini et al., 2010). Some similarities are also evident between these tracks and those designated *Albertasuchipes* from the Palaeocene of Canada (McCrea, Pemberton & Currie, 2004). The crocodyliform tracks at the Snake Creek Tracksite seem to match the descriptions of underwater walking tracks made by modern crocodylians presented by Farlow et al. (2018a), especially given that only claw marks are generally visible. However, the fact that so many relatively continuous crocodyliform trackways have been identified at the Snake Creek Tracksite is somewhat unusual, since the footfall patterns in many fossilised crocodyliform swimming trackways (Lockley et al., 2014) and of modern crocodiles (Farlow et al., 2018b) are often far less regular or clear. It is possible that the relatively consistent pattern of the crocodyliform trackways at the Snake Creek Tracksite was imposed by the depth of the water: it might have been sufficiently deep that the bodies and tails of the trackmakers were buoyed up, but not so deep that they could propel themselves through the water without touching down regularly.

**Turtle tracks.**

Ichnogen. et ichnosp. indet.

**Figure 30:**
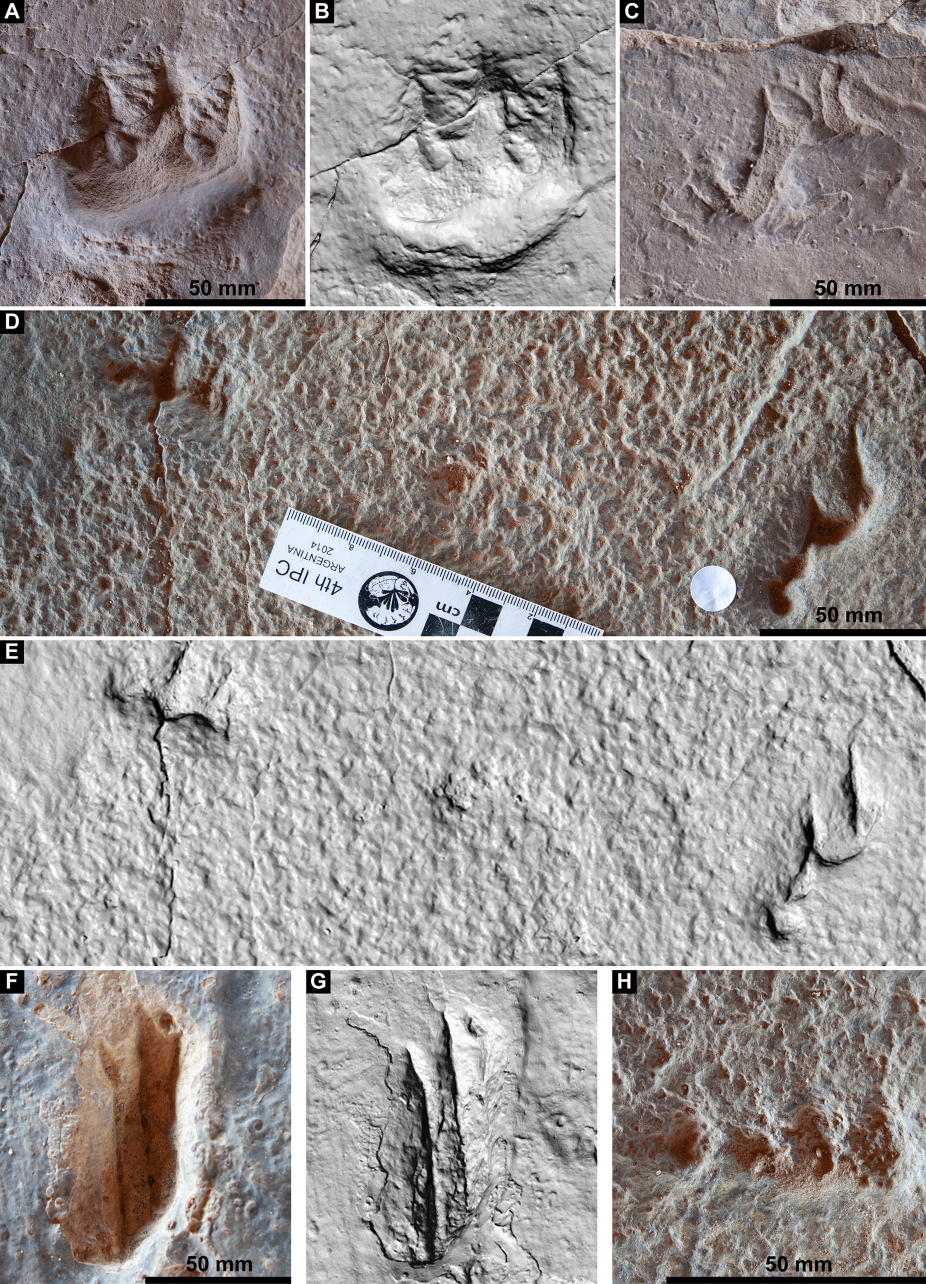
Possible turtle tracks. (A) Photograph of a tridactyl track from the southwest of the site, showing possible interdigital adhesion traces and partial infill, illuminated from the east (page left). (B) Monochrome surface model of the same, illuminated from the south (page top). (C) Possible right tridactyl (possibly tetradactyl) track from the southwest of the site, preserved as an infilled concave epirelief, illuminated from the east (page left). (D) Possible left and right tetradactyl tracks from the southwest of the site, showing inward rotation of the feet relative to the direction of travel, illuminated from the east (page left). (E) Monochrome surface model of the same, illuminated from the north (page bottom). (F) Elongate tridactyl swim track from the northwest of the site, illuminated from the east (page left). (G) Monochrome surface model of the same, illuminated from the northeast (page bottom left). (H) Tetradactyl track from the southwest of the site, illuminated from the east (page left).

**Referred tracks.** Various isolated tracks, some of which might form a trackway (Fig. 30).

**Horizon and locality.** ‘Upper’ Winton Formation, Cenomanian–?lowermost Turonian (Tucker et al., 2013); Snake Creek Tracksite (AODL 251), Karoola Station, NW of Winton, Queensland, Australia.

**Description.** Several isolated non-sauropod tracks from the Snake Creek Tracksite are not readily assignable to any of the morphotypes listed above. Some of these are tridactyl (Figs. 30A–30B, 30D–30E (left), 30F–30G), whereas others either appear to be tetradactyl (Figs. 30C, 30D–30E (right)), or definitively are (Fig. 30H). All appear to be compatible with turtle tracks (Avanzini et al., 2005), although each assignation is tentative.

The best preserved possible turtle track (Figs. 9E, 30A–30B) is situated in the southwest of the site (Fig. 11). It is tridactyl, with the digits more or less equal in length; the median digit is the longest by a small margin. The tips of the digit impressions are pointed, and small bulges are present at the interdigital junctions; these might be indicative of webbing. The heel is straight but inclined; in other words, the posterior margin of the track is closer to the tip of the right digit than it is to that of the left digit. A prominent displacement bulge is present posterior to the track. This track is extremely similar to tracks attributed to turtles from the Early Cretaceous of China (Xing et al., 2014: fig. 4) and Spain (Moratalla, Sanz & Jiménez, 1990), although it also shares similarities with some crocodyliform tracks as well (e.g., Avanzini et al., 2010: fig. 2A).

Another track (Fig. 9H), situated near the track described above, is morphologically similar but has evidently only been exposed via transmission into an overlying sediment layer. Thus, this track is evinced only by three digit impressions of approximately equal length, seemingly united by a straight posterior margin. A third track (Figs. 30D–30E (left)), situated somewhat further northeast, is also similar and might preserve a fourth digit impression.

Two other tracks that are similar to each other, but unlike all others at the Snake Creek Tracksite, are also present in the southwest of the site. The first of these (Figs. 9D, 30C) is situated approximately 300 mm southwest of (and therefore anterior to) the best preserved tridactyl possible turtle track. The track in question remains infilled and is accompanied anteriorly by a circular section of overburden that probably obscures another track; attempts to remove this without damaging the tracked surface proved futile. The second track (Figs. 30D–30E (right)) is preserved as a concave epirelief with no infill, and the following description is largely based on it. It appears to be tetradactyl, with two of the digit impressions much more prominent than the remaining two. If we assume that this track pertains to the right manus, based on a trackway made by a modern European pond turtle (*Emys orbicularis*) walking through shallow water on fine sand (Avanzini et al., 2005), then digits III and IV are more prominent than I and II. Indeed, the latter two digits are manifested as little more than small, circular depressions, with that of I slightly larger than II. The digit III impression is small and triangular with an anterior claw drag mark, whereas the digit IV impressions is similar but more elongate with a similarly prominent claw drag mark. The track appears to be inwardly rotated; if this track is associated with the track immediately to its left (Figs. 30D–30E (left)), then this is indeed the case. Another possible turtle track (Fig. 30H) comprises a row of four small circular depressions, although we note that this track does show some similarity to a track assigned to *Hatcherichnus* (Lockley & Foster, 2010).

The final possible turtle track (Figs. 30F–30G) is a clear swim track, comprising three parallel, elongate striae. It is substantially deeper than most of the other non-sauropod tracks at the Snake Creek Tracksite, and is strikingly similar to a possible turtle swim track from China described by Xing et al. (2016b).

**Discussion.** The tracks described above closely resemble those made by extant bottom-walking turtles (Avanzini et al., 2005: fig. 10E), and are consequently interpreted as such. Curiously, although the body fossil record of freshwater and terrestrial turtles in Australia spans the Early Cretaceous–Recent (Gaffney, 1981; Gaffney et al., 1998), fossilised tracks pertaining to turtles have never been reported from Australia.

Few turtle tracks have been reported from the Mesozoic of Gondwana. The Triassic record of turtle tracks is restricted to Europe and North America (Lichtig et al., 2018; Lovelace & Lovelace, 2012; Reolid et al., 2018; Von Lilienstern, 1939; Thomson & Lovelace, 2014), as is the Jurassic record (Avanzini et al., 2005; Bernier et al., 1982; Foster, Lockley & Brockett, 1999; Gaillard et al., 2003; Lockley & Foster, 2006; Romano & Whyte, 2010). Although Early Cretaceous turtle tracks are more widespread, with numerous reports from Europe (Fuentes Vidarte et al., 2003; Hornung & Reich, 2013; Moratalla, Sanz & Jiménez, 1990; Pascual-Arribas & Hernández-Medrano, 2015; Moratalla & Hernán, 2009), Asia (Kim & Lockley, 2016; Lockley et al., 2015; Lockley et al., 2018c; Lockley, Xing & Xu, 2019; Xing et al., 2014; Xing et al., 2016b; Xing et al., 2019a), and North America (McCrea et al., 2014; Lockley et al., 2018d), the only Gondwanan records are from Croatia (then part of Gondwana; Mezga & Bajraktarević, 2004), Morocco (Klein et al., 2018) and Brazil (Dentzien-Dias et al., 2010). The Late Cretaceous record is largely confined to North America (Fiorillo, 2005 (although see Lichtig et al., 2018); Lockley et al., 2010a; Lockley et al., 2014; Lockley et al., 2018d; Lockley, Smith & King, 2018; McCrea et al., 2014; Wright & Lockley, 2001), with rare reports from Tunisia (Contessi & Fanti, 2012) and Morocco (Belvedere et al., 2013).

Although the tracks from the Snake Creek Tracksite assigned to turtles herein show some degree of morphological variation, each can be aligned with turtle tracks reported from elsewhere, as outlined above. All of the turtle tracks described herein appear to have been made by individuals that were buoyed up by the water column, and only occasionally making contact with the substrate.

**Fish feeding traces.**

Ichnogen. et ichnosp. indet.

**Figure 31:**
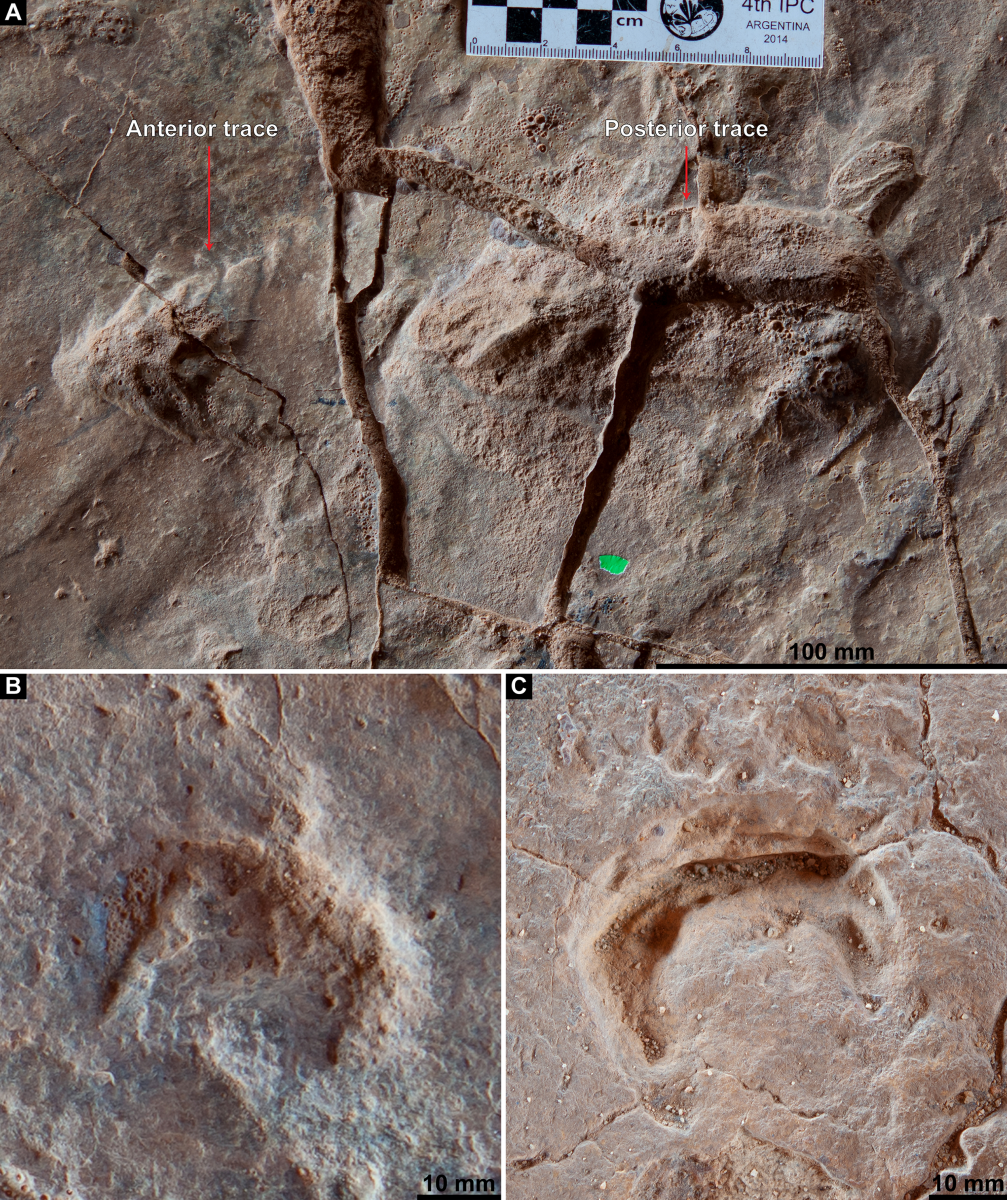
Probable fish feeding traces. (A) Sturgeon-like ?fish feeding trace, illuminated from the east (page bottom). (B) ?Lungfish feeding trace from the northwest of the Tracksite, illuminated from the east (page left). (C) ?Lungfish feeding trace from the southwest of the Tracksite, illuminated from the east (page left).

**Referred tracks.** Various isolated probable fish feeding traces (Fig. 31).

**Horizon and locality.** ‘Upper’ Winton Formation, Cenomanian–?lowermost Turonian (Tucker et al., 2013); Snake Creek Tracksite (AODL 251), Karoola Station, NW of Winton, Queensland, Australia.

**Description.** Two distinct types of fish feeding traces have been recognised at the Snake Creek Tracksite. The first is less common, being represented by only a single ichnite preserved in the southwest of the site (Fig. 11), and tentatively attributed to an actinopterygian (Fig. 31A). The other is represented by at least three ichnites, two of which are illustrated (Figs. 31B–31C), that are fairly widely dispersed along the western margin of the Tracksite (Figs. 11–12).

**Fish feeding trace I.** The first fish feeding trace (Fig. 31A) is approximately 250 mm long anteroposteriorly and comprises two associated indentations. The anterior trace is wedge-shaped and symmetrical, has a slightly rounded anterior tip, and is deepest medially. It is approximately 60 mm long anteroposteriorly, and the posterior margin is approximately 55 mm wide transversely. Internally, this trace appears to be terraced: four subdivisions are evident, approximately evenly spaced, with the anterior margin of each raised and the posterior margin lowered. Each terrace forms a continuous, chevron-like ridge that interrupts each lateral margin. The gap between the anterior and posterior traces is 40 mm long anteroposteriorly. The posterior trace constitutes an ovoid depression, 150 mm long and 85 mm wide, with a raised, radially sloping platform situated on the midline.

**Interpretation.** Superficially, this trace fossil resembles feeding traces made by the modern day sturgeon *Acipenser oxyrinchus* (Pearson et al., 2007). Although Australia’s Mesozoic fossil fish record is extensive (Berrell et al., 2020), the only actinopterygian fish described from the Winton Formation to date is *Cladocyclus geddesi* (Berrell et al., 2014). This taxon is known only from the ‘lower’ Winton Formation, the depositional environment of which was marginal marine (Syme et al., 2016) and therefore a stark contrast to the freshwater floodplain setting inferred for the ‘upper’ Winton Formation (Fletcher, Moss & Salisbury, 2018). We tentatively attribute fish feeding trace I to an actinopterygian fish, despite the present near lack of body fossil evidence for actinopterygians in the ‘upper’ Winton Formation.

**Fish feeding trace II.** The other fish feeding traces at the Snake Creek Tracksite are morphologically uniform (Figs. 31B–31C). Broadly speaking, these indentations are ∩-shaped with a slightly convex anterior margin (~40 mm wide transversely) and abbreviate, straight and parallel lateral processes (~25 mm long anteroposteriorly). In less well-preserved exemplars (Fig. 31B), little detail is preserved beyond a featureless, ∩-shaped concavity; by contrast, in one particularly excellent ichnite (Fig. 31C), much internal detail is preserved. The indentation is most pronounced anteriorly, with the depth of the lateral process decreasing posteriorly. The anterior margin is stepped: the lower section forms a continuous ridge, whereas the upper section undulates. The deepest portion of the trace lies immediately beneath the lower anterior ridge and forms a continuous depression. Posterior to this, a shallow shelf is present, broadest along the midline and pinched in anterolaterally, and set at a slightly lower level than the surface between the lateral processes. The two lateral processes are deepest medially.

**Interpretation.** The ∩-shaped traces are herein tentatively identified as lungfish feeding traces (praedichnia), based on the overall similarity between their shape and the snout/upper jaw of many post-Palaeozoic lungfish (Bemis, 1986; Kemp, 1998; Kemp, 1999). Despite the widespread distribution of lungfish throughout the Palaeozoic and Mesozoic (Clack, Sharp & Long, 2011; Schultze, 2004), feeding traces of lungfishes have only been rarely reported, being presently restricted to the Early Devonian of Poland (Szrek et al., 2016) and the Late Devonian of China (Fan, Zong & Gong, 2019). These traces have been attributed to the ichnogenus *Osculichnus*, which was originally described from the Paleogene of Turkey (Demírcan & Uchman, 2010).

Lungfish toothplates have been known from the Winton Formation since at least the 1980s (Kemp, 1982; Kemp, 1991; Kemp, 1997). Recently, Kemp & Berrell (2013: 503) stated the following: “Few of (the Winton Formation) tooth plates show any evidence of disease, and wear is smooth. This suggests that they ate soft food, and that the environment in which they lived was clean. The tooth plates from this deposit are often large, suggesting a long life untroubled by problems of overcrowding or of getting enough to eat.” In light of this, and the morphology of the ichnites, it is tempting to suggest that the traces at the Snake Creek Tracksite were made by lungfish that were feeding on soft-bodied benthic organisms (animals, plants or microbial mats) or infaunal invertebrates.

## Discussion

### Interpretation of the Snake Creek Tracksite

The Snake Creek Tracksite preserves tracks that were undoubtedly made subaqueously, as well as others that appear to have been made subaerially or in emergent substrate. Thus, it appears to be a time-averaged ichnoassemblage, although the precise timespan it represents is unclear. The levee present along the northern margin of the Tracksite appears to have been in place before any of the tracks were made: the sauropod trackmakers appear to have turned northwest to avoid it, and although there are no tracks preserved within it, there appears to be evidence of burrowing.

Some sections of the Snake Creek Tracksite preserve indistinct depressions that probably represent traces of sauropod passage formed before the substrate was sufficiently drained to hold the shape of a track: a clear example of this is the depression present lateral to the pes track of AODF 904.S1.09 (Fig. 15B), which appears to be but one track in a sauropod trackway that antedates all of the other sauropod trackways preserved at the Snake Creek Tracksite and runs effectively perpendicular to them (southwest–northeast). The section of the site preserving the *Skartopus* trackway AODF 904.T1 also preserves three slightly divergent, fusiform depressions (Fig. 21), each less than 500 mm long along its long axis, that might be resting traces that were made by lungfish or crocodyliforms when the tracked surface was not sufficiently coherent to retain much detail beyond rippled outlines.

The sauropod turning trackway (AODF 904.S3) appears to have been made prior to the two longest continuous sets of sauropod tracks preserved at the Snake Creek Tracksite (Figs. 8, 10). We suggest that this was so because there is no pes track associated with manus track AODF 904.S3.06; instead, the area in which it would be expected to be preserved is occupied by AODF 904.S1.11. Furthermore, we suggest that this track overprinted and obliterated the pes track of AODF 904.S3.06. Similarly, the possible theropod penetrative track in the northwest of the site must antedate the sauropod trackway AODF 904.S1, given that significant distortion of this track appears to have been caused by the sauropod manus track AODF 904.S1.23.

It seems likely that, of the two more or less parallel southwest–northeast trending sauropod trackways, AODF 904.S2 was formed slightly before AODF 904.S1: the two trackways cross over and could not have been made absolutely synchronously. Both AODF 904.S2 and AODF 904.S1 appear to have been made when the surface of the substrate was subaerially exposed. This is inferred because the prominent displacement rims (raised margins of lithified siltstone) that encircle many, if not all, of the sauropod tracks, particularly at the southeast end of the Tracksite, host multiple effectively concentric ridges, apparently produced by contraction of the displaced substrate as it dried under subaerial conditions. These features are similar to the concentric “desiccation cracks” surrounding a dinosaur track from the Cretaceous of South Korea (Paik, Kim & Lee, 2001: p. 87; fig. 7), the “sub-concentrical fractures” or “marginal cracks” accompanying a presumed dinosaur track from the Cretaceous of Italy (Nicosia et al., 2007: p. 73, fig. 10), and the “crinkled edges” on the lateral margin of an artiodactyl track from the Neogene of California by Scrivner & Bottjer (1986: p. 316; fig. 14b). For a sedimentary feature such as this to have formed and been preserved, the tracked surface is presumed to have been exposed subaerially when it was traversed by the sauropods.

The absence of cracks throughout the rest of the Snake Creek Tracksite suggests that the tracked surface was submerged shortly after the formation of the sauropod tracks, with insufficient time for desiccation of the tracked surface to take place in the interim. Few, if any, of the small tridactyl tracks appear to have been made before this inundation took place: perhaps the most likely exceptions are some of the *Wintonopus* tracks scattered throughout the site, some of which are less clear than the other tridactyl tracks. The submergence of the tracked surface does not appear to have resulted in the loss of any detail on the sauropod tracks, suggesting that the substrate was both saturated and quite tenacious despite remaining some degree of plasticity.

Most, if not all, of the tracks made by theropods, crocodyliforms, and possibly turtles and fish, appear to have been made after the sauropod tracks, and most of these were made by swimming individuals. This suggests that the water level rose shortly after the formation of the sauropod tracks. The best evidence for this order of events stems from the crocodyliform trackway AODF 904.C1: tracks AODF 904.C1.31 and AODF 904.C1.32 disrupt some of the ridges present on the displacement rim of the sauropod track AODF 904.S1.07, indicating that they were made after the formation of the ridges (Fig. 9B). The crocodyliforms at least were largely buoyed up by the water column and were walking underwater, often touching down with only their hind feet. The morphological congruence that these tracks share with swim track from other sites around the world supports this interpretation (Milner & Lockley, 2016).

### Reconciling the body fossil and ichnofossil records of the ‘upper’ Winton Formation

Prior to the discovery of the Snake Creek Tracksite, the vertebrate ichnofossil record of the ‘upper’ Winton Formation (Table 3) comprised functionally tridactyl tracks, interpreted as being from small to medium-sized ornithopod and theropod dinosaurs (Romilio & Salisbury, 2011; Romilio & Salisbury, 2014; Romilio, Tucker & Salisbury, 2013; Thulborn, 2013; Thulborn, 2017; Thulborn & Wade, 1979; Thulborn & Wade, 1984; Thulborn & Wade, 1989). The description of the Snake Creek Tracksite presented here allows sauropod, crocodyliform, and ?turtle tracks, and possible lungfish and actinopterygian fish feeding traces, to be added to that list. To date, no other vertebrate tracks have been reported from the Winton Formation.

**Table 3:** ‘Upper’ Winton Formation invertebrate and vertebrate fossil record.

Property	Site name	QM locality	AAOD locality (AODL)	Year(s) excavated	Hexapoda	Gastropoda	Bivalvia	Actinopterygii	Dipnoi	?Sauropterygia	Lepidosauria	Testudines	Crocodyliformes	Pterosauria	Ankylosauria	Ornithopoda	Theropoda	Sauropoda
Selwyn Park	–	–	–	1952	–	–	–	–	–	–	–	–	–	–	–	–	–	B (QM F10916: *Wintonotitan wattsi*)
Alni	–	31	–	1964	–	–	–	–	–	–	–	–	–	–	–	–	–	B (QM F3390)
Lovelle Downs	–	244	–	1972	–	–	–	–	–	–	–	–	–	–	–	–	–	B (QM F7291)
Lovelle Downs	Joyce	245	–	1972, 2012	–	–	–	–	–	–	–	–	–	–	–	–	–	B (QM F7880)
Elderslie	Triangle Paddock (=Clancy)	313	55	1974, 2004, 2006	W	M	–	–	P	–	–	C	O,T	–	–	–	T	S (QM F7292: Wintonotitan wattsi*)
Elderslie	–	314	–	1974	–	–	–	–	–	–	–	–	–	–	–	–	–	B
Lovelle Downs	–	315	–	1974	–	–	–	–	–	–	–	–	–	–	–	–	–	S (QM F6737)
Mount Cameron	Seymour†, Lark†, and New† quarries	–	10 (Lark: plants, bivalves)	1976–1977	–	–	–	–	–	–	–	–	–	–	–	I (QM F10319, etc.: Wintonopus latomorum*)	I (QM F10330, etc.: Skartopus australis*); I (cf. *Tyrannosauropus*)	–
Elderslie	–	1,454	–	–	–	–	–	–	–	–	–	–	–	–	–	–	–	B
Belmont	Lungfish	–	8	1985	–	–	–	–	P	–	–	–	–	–	–	–	–	–
Belmont	Bore Paddock 2	–	–	1990	–	–	–	–	–	–	–	–	–	–	–	–	–	B
Belmont	Bore Paddock 1	–	6	1997	–	–	–	–	–	–	–	–	–	–	–	–	–	B
Belmont	Bore Paddock 3	–	4	1998	–	–	–	–	–	–	–	–	–	–	–	–	–	B
Belmont	Riley’s Paddock	–	9	2001	–	–	–	–	–	–	–	–	–	–	–	–	–	B
Belmont	Elliot (=Kylie’s Corner, =Alex)	1,333	1/126/127	2001–2005	W	–	M	B	P	T	–	C	B,O,T	B	T	–	?B, T	B, S (AODF 836: *Diamantinasaurus matildae*), T
Belmont	Excess	–	2	2002	–	–	–	–	–	–	–	–	–	–	–	–	–	?B
Elderslie	†	–	94	2003	–	–	–	–	–	–	–	–	?I	–	–	–	?I	–
Undisclosed	Mick	–	49	2003	–	–	–	–	–	–	–	–	–	–	–	–	–	S
Belmont	Overshot	–	52	2004	–	–	M	B	P	T	B (QM F52673)	C	B,O,T	–	B	T (QM F52774)	T	B
Belmont	Ho-Hum (=Wade)	–	82	2005, 2012	–	–	–	–	–	–	–	–	–	–	–	–	T (AODF 819)	S (AODF 660: Savannasaurus elliottorum*)
Belmont	Boxing Day (=Packer)	–	83	2005	–	–	–	–	–	–	–	–	–	–	–	–	–	B
Belmont	Bob	–	80	2006	–	–	–	–	–	–	–	–	–	–	–	–	–	B
Belmont	Bob South	–	81	2006	–	–	–	–	–	–	–	–	–	–	–	–	–	B
Elderslie	Matilda (=Banjo)	–	85	2006–2010	–	–	–	–	P	–	–	C	B,O,T	–	–	–	S (AODF 604: Australovenator wintonensis*), T (AODF 822–831)	S (AODF 603: Diamantinasaurus matildae*), T
?Elderslie	Coolibah	–	86	2008	–	–	–	–	–	–	–	–	–	–	–	–	–	B
Elderslie	McKenzie	–	79	2010	–	–	–	–	–	–	–	–	–	–	–	–	–	B
Elderslie	Munro	–	118	2010	–	–	–	–	P	–	–	–	–	–	–	–	–	S
Elderslie	Chookie (=Sparkles)	–	120	2010–2011	–	–	–	–	–	–	–	–	S	–	–	–	–	B
Elderslie	Dixie (=Dicks Creek)	–	117	2011	–	–	–	–	–	–	–	–		–	–	–	–	S
Elderslie	Oliver	–	122	2012	–	–	–	–	–	–	–	–	–	–	–	–	–	S
Elderslie	Shearing Shed Paddock × 2	–	123	–	–	–	–	–	–	–	–	–	–	–	–	–	–	B
Elderslie	Olga	–	121	2012	–	–	–	–	–	–	–	–	–	–	–	–	–	B
Elderslie	Eagle’s Nest	–	135	2012	–	–	M	–	–	–	–	–	–	–	–	–	–	B
Elderslie	Pegler’s	–	124	2012	–	–	–	–	–	–	–	–	B	–	–	–	T (AODF 664)	B
Elderslie	Trixie (=Pete)	–	125	2012–2013	–	–	–	–	–	–	–	–	B	–	–	–	T (AODF 820)	S
Lovelle Downs	Patrice	–	160	2014	–	–	–	–	–	–	–	–	–	–	–	–	–	B
Karoola	Karoola 1	–	213	2014	–	–	–	–	–	–	–	–	–	–	–	–	–	B
Karoola	Karoola 2	–	214	2014	–	–	–	–	–	–	–	–	–	–	–	–	–	B
Lovelle Downs	Mary	–	161	2014	–	–	–	–	–	–	–	–	–	–	–	–	–	B
Lovelle Downs	Jenna	–	163	2014–2015	–	–	–	–	–	–	–	–	–	–	–	–	–	B
Lovelle Downs	Darcy	–	162	2015	–	–	–	–	–	–	–	–	–	–	–	–	–	B
Elderslie	Ian	–	215	2015	–	–	–	–	–	–	–	–	–	–	–	–	–	B
Elderslie	Kate 1	–	216	2015	–	–	–	–	–	–	–	–	–	–	–	–	–	B
Elderslie	Kate 2	–	217	2015	–	–	–	–	–	–	–	–	B	–	–	–	–	–
Elderslie	Kate 3 (=Croc)	–	218	2015	W	–	–	–	–	–	–	–	S	–	–	–	–	–
Elderslie	Matt	–	219	2015	–	–	–	–	–	–	–	–	–	–	–	–	–	B
Lovelle Downs	Mikey	–	226	2016	–	–	–	–	P	–	–	–	–	–	–	–	–	B
Lovelle Downs	Moggsie	–	227	2016	–	–	–	–	–	–	–	C	–	–	–	–	?B	B
Belmont	Devil Dave	–	128	2016–2017	–	–	–	–	–	–	–	–	–	–	–	–	T	B
Belmont	Wardoo (=Pterosaur; =Butch)	–	245	2017	–	–	–	–	–	–	–	–	–	S (AODF 876: Ferrodraco lentoni*)	–	–	–	–
Belmont	Son of the Devil (=Judy)	–	246	2017	–	–	–	–	–	–	–	C	–	–	–	–	T	S,T
Belmont	Judy 2	–	247	2017	–	–	–	–	–	–	–	–	–	–	–	–	–	B
Karoola	Snake Creek Tracksite†	–	251	2018–2020	–	–	–	?I	?I	–		?I	I (AODF 904.C: *Hatcherichnus*)	–	–	I (AODF 904.O: *Wintonopus latomorum*)	I (AODF 904.T: *Skartopus australis*)	I (AODF 904.S)
Elderslie	Ann	–	252	2018	–	–	–	–	–	–	–	–	B	–	–	–	–	S,T
Elderslie	Marilyn	–	261	2018	–	–	–	–	P	–	–	–	–	–	–	–	S (AODF 967–968, 972, 977–979)	B
Elderslie	Mitchell	–	270	2019	–	–	M	–	P	T	–	–	B,O,T	T	–	–	T	S,T

**Note:**

Described specimens are listed by specimen number. A dagger (†) next to a locality name indicates that it is an ichnofossil locality. An asterisk (*) next to a species name indicates that the holotype of that species derives from that site. Note that the bivalves described by Hocknull (1997, 2000), the lungfish toothplates described by Kemp (1991, 1997), and several unexcavated sites are not included. B = five or fewer bones; C = carapace/plastron fragment(s); I = ichnite(s); M = mollusc(s); O = osteoderm(s); P = toothplate(s); S = more than five bones; T = tooth/teeth; W = wing(s) and/or elytrum/elytra.

In stark contrast to the ichnofossil record (prior to the discovery of the Snake Creek Tracksite), the body fossil record of the ‘upper’ Winton Formation is dominated by sauropods (Table 3). Of the more than forty sauropod body fossil specimens discovered to date, only nine have been described (Coombs & Molnar, 1981; Hocknull et al., 2009; Poropat et al., 2015a; Poropat et al., 2015b; Poropat et al., 2016; Poropat et al., 2019a; Poropat et al., 2020; Poropat et al., 2021). Theropods are the next best represented dinosaurs in the ‘upper’ Winton Formation, yet their record comprises only two associated skeletons and numerous isolated teeth (Hocknull et al., 2009; White et al., 2012; White et al., 2013; White et al., 2015a; White et al., 2015b; White et al., 2016; White et al., 2020). Body fossils of ornithischian dinosaurs are rare in the ‘upper’ Winton Formation, with only four teeth reported—one from a small-bodied ornithopod (Herne et al., 2019; Hocknull & Cook, 2008), and three from ankylosaurs (Leahey & Salisbury, 2013). This contrasts markedly with the ‘lower’ Winton Formation, the only dinosaurs known from which are undescribed ornithopods (Salisbury et al., 2019; Syme et al., 2016). Although they have not been described in detail, crocodyliform teeth and osteoderms, chelid turtle carapace and plastron fragments (Hocknull et al., 2009), and ceratodontoid lungfish toothplates (Kemp, 1991; Kemp, 1997) are among the most common vertebrate fossils (other than sauropods) recovered from the ‘upper’ Winton Formation (Table 3). Two partial crocodyliform skeletons with skulls have also been identified, but have not yet been described (White et al., 2019). The ‘upper’ Winton Formation has yielded rare evidence of anhanguerian pterosaurs (i.e. *Ferrodraco lentoni*: Pentland et al., 2019), varanoid lizards (Kear, 2016; Scanlon & Hocknull, 2008), and actinopterygian fish (S. F. Poropat and D. A. Elliott, 2019, personal observation). By contrast, the ‘lower’ Winton Formation has produced articulated skulls and skeletons of crocodyliforms (Salisbury et al., 2006; Syme & Salisbury, 2018) and actinopterygian fish (Berrell et al., 2014), but no evidence of turtles or lungfish (Syme et al., 2016).

The summaries of the vertebrate body fossil and ichnofossil records of the ‘upper’ Winton Formation presented above (and in Table 3) demonstrate substantial incongruence. The Winton Formation would be classified as a Type 4b deposit sensu Lockley (1989, 1991), since the body fossil record has contributed more to our understanding of the vertebrate fauna than the ichnofossil record, and the faunal composition revealed by the body fossils has historically been divergent from that attested to by the tracks. The Snake Creek Tracksite goes some distance towards redressing this balance, and concurrently demonstrates that the environments in the Winton Formation that were favoured by small-bodied theropods and ornithopods, and that preferentially preserved tracks rather than body fossils, did not necessarily exclude sauropods, crocodyliforms or turtles. The question this observation poses is this: why the bias in the body fossil record towards sauropod dinosaurs (and, to a lesser extent, crocodyliforms), and why the bias in the ichnofossil record towards small, functionally tridactyl dinosaurs? Our proposed answer to this question is dependent on several factors.

The first factor is the nature of the Winton Formation itself. The top ~100 m of the Winton Formation (and, where its thickness is less than 100 m, the underlying Mackunda Formation) were subjected to substantial chemical weathering during the Cainozoic, long after the cessation of sedimentation in the Eromanga Basin (Senior, Mond & Harrison, 1978). Thus, despite its great lateral extent, Winton Formation outcrop is limited to nodular sandstones, as well as erosion-resistant mesas comprising chemically weathered silt- and sandstones (Cook, Bryan & Draper, 2013; Syme et al., 2016). The rest of the Winton Formation is blanketed by montmorillonite-rich ‘black soil’ (Senior, Mond & Harrison, 1978). When desiccated, this soil forms deep cracks (up to 1.5 m deep) that propagate into more consolidated Winton Formation sediments below. When hydrated by rain, the soils expand, causing cracks to close and/or become infilled with sediment. The ultimate effect of this process is effectively rotational as the topsoil and open-sided cracks are periodically dissolved and washed down to the base of the ‘black soil’ profile. As fossilised bones become incorporated into the ‘black soil’ profile, the expansion of hydrated soil that has been wedged into fractures within the fossil causes fragmentation; unlike their host sediments, the bones cannot deform in a ductile fashion. Repeated soil rotation over hundreds of years ultimately brings the fragmented bones to the surface. The time spent by the bones within the soil profile is critical: the longer they are exposed to the destructive tendencies of ‘black soil’, the more they degenerate. This poses a problem for smaller fossils as, in effect, they are being continually washed back down the cracks long before they get anywhere near the surface. These fossils remain in the mid-to-lower reaches of the soil profile where they gradually break down completely, having never reached the surface where they might be identified by someone passing by.

The second factor is how fossils are preserved in the Winton Formation. As far as we are aware, all of the body fossils heretofore reported from the ‘lower’ Winton Formation have been discovered in *ex situ* sandstone concretions (Syme & Salisbury, 2018; Syme et al., 2016). By contrast, relatively few fossils in the ‘upper’ Winton Formation are preserved in concretions: an actinopterygian fish, two partial crocodyliforms, and perhaps 15% of the sauropod specimens recovered to date (D. A. Elliott, T. Sloan, M. A. White, & S. F. Poropat, 2002–2020, personal observation). The impact of soil rotation on a fossil would presumably be diminished if said fossil were preserved within a concretion; however, this would only hold true if the concretion remained more or less intact. Most vertebrate fossils in the ‘upper’ Winton Formation are found in deeply weathered, poorly consolidated claystone or siltstone layers, with a few rare examples preserved within similarly poorly consolidated gypsum-rich concretions hosted in such layers.

The final factor that impacts the nature of the body fossil record of the ‘upper’ Winton Formation is how the fossils are found. Almost every site listed in Table 3 was discovered by a grazier, either mustering stock, spraying weeds, or (more rarely) systematically searching for new field sites on a property. Very few of these sites were discovered by professional palaeontologists: examples include Lark Quarry, New Quarry, and the ‘Matt’ site (Table 3).

The combination of the factors listed above makes it somewhat unsurprising that sauropods are more commonly recovered in the ‘upper’ Winton Formation than any other vertebrate group: their bones are more likely to remain larger and more recognisable than those of other vertebrates, thus they are consequently more readily located at the surface. Almost every vertebrate fossil site in the ‘upper’ Winton Formation discovered to date has been identified on the basis of sauropod fragments at the surface. The two notable exceptions, both discovered by grazier and veteran fossil finder Bob Elliott, are the ‘Wardoo’ site, which produced the holotype specimen of the pterosaur *Ferrodraco lentoni* (Pentland et al., 2019); and the ‘Marilyn’ site, which produced a fragmentary megaraptorid theropod skeleton (White et al., 2020). Neither of these exceptions were located on fracturing ‘black soil’ country: the Wardoo site was situated on the banks of a periodically active creek (Pentland et al., 2019), whereas the Marilyn site was predominantly on a scalded claypan (White et al., 2020). Targeted searches of modern day channels and claypans in the Winton area might enable additional discoveries of non-sauropod sites.

The mode of preservation of the Snake Creek Tracksite—a consolidated layer overlain and underlain by unconsolidated sediment—is unlike that of any ichnofossil locality yet identified in the Winton Formation. Prior to the discovery of the Snake Creek Tracksite, the only tracksites known from the Winton Formation were hosted in the chemically weathered sand- and siltstones (duricrusts, laterites, silcretes) of erosion-resistant mesas, locally termed ‘jump-ups’ (McKenzie, 2004; Romilio & Salisbury, 2011; Romilio & Salisbury, 2014; Romilio, Tucker & Salisbury, 2013; Thulborn, 2013; Thulborn, 2017; Thulborn & Wade, 1979; Thulborn & Wade, 1984). Three of the four reported tracksites—Seymour Quarry, New Quarry, and the unnamed Elderslie site—have never been excavated, and their tracked surfaces have barely been exposed. Whereas the unnamed Elderslie site is an isolated layer of track casts (convex hyporeliefs) that was so broken up that there was no way of identifying trackways, both Seymour Quarry and New Quarry are inferred to be quite extensive. As track-bearing exposures at these sites terminate under tens of metres of sedimentary overburden, full excavations appear impractical. More than 200 m^2^ of overburden was moved to expose the tracked surface at Lark Quarry, but the total thickness of the removed strata was less than one metre (Thulborn & Wade, 1984).

The fact that ~35% of the 48 sauropod body fossil sites in the ‘upper’ Winton Formation that are hosted in areas of active ‘black soil’ have also produced non-sauropod vertebrate remains (mostly pertaining to crocodyliforms, turtles and lungfish; Table 3), coupled with the preponderance of theropod and ornithopod tracks in the ichnofossil record, suggests that future discoveries of body fossils of small-bodied dinosaurs and other vertebrates can be expected in the ‘upper’ Winton Formation, especially if more time can be dedicated to finding and excavating sites in future. Given the vast extent of the ‘upper’ Winton Formation, the future use of novel technologies and somewhat unconventional methods (e.g., Kaye & Pittman, 2020) might reap rich rewards.

## Conclusion

The Snake Creek Tracksite sheds much light on the composition of terrestrial and freshwater vertebrate faunas in northeast Australia during the early Late Cretaceous. This time-averaged ichnoassemblage preserves trackways of sauropods and crocodyliforms, the two vertebrate groups that dominate the body fossil record of the ‘upper’ Winton Formation, alongside tracks of theropods and ornithopods—the heretofore best represented taxa in the ichnofossil record of the same unit. The preservation of these tracks, in addition to possible turtle tracks and lungfish and actinopterygian fish feeding traces, provides a unique palaeoenvironmental snapshot of the region, supporting previous interpretations that the Winton Formation was deposited on a wet, temperate, low relief floodplain that was rife with meandering rivers and oxbow lakes, and often characterised by overbank deposits.

The sauropod tracks described herein appear to be distinct from all other sauropod tracks worldwide, but are not sufficiently well-preserved to warrant the erection of a new ichnotaxon. Multiple walking-pace trackways, which display a unique combination of a wide-gauge stance and gait, a mild–moderate degree of heteropody, and manus tracks with prominent ungual impressions on digit I, are preserved, and are ascribed to an early branching titanosaur. Collectively, the sauropods trackways at the Snake Creek Tracksite—which were made within a short timeframe, albeit not precisely synchronously—provide some support for the notion that sauropods were gregarious. In addition, the partial sauropod turning trackway preserved at the Snake Creek Tracksite—which is herein attributed to a titanosaur—reinforces the previously advanced hypothesis that titanosaurs were more manoeuvrable and agile than other sauropods.

The Snake Creek Tracksite preserves new tracks attributable to the small theropod ichnotaxon *Skartopus australis* and the small ornithopod ichnotaxon *Wintonopus latomorum*. However, many other non-sauropodan tracks preserved at the Tracksite, thought to pertain to small theropods at the time of their discovery, are in fact attributable to the crocodyliform ichnotaxon *Hatcherichnus*. The presence of crocodyliform underwater walking trackways, isolated possible turtle tracks, and probable feeding traces assigned to both lungfish and actinopterygian fish at the Snake Creek Tracksite, indicates that it was, for the most part, subaqueous at the time of track formation (the sauropod tracks notwithstanding).

The Snake Creek Tracksite appears to preserve evidence of all of the most common vertebrate groups found in the Winton Formation within a relatively small area. This suggests that the apparent incongruence between the vertebrate ichno- and body fossil records might not be a consequence of environmental segregation of sauropods and crocodyliforms in areas more likely to preserve body fossils, and theropods and ornithopods in settings conducive to track preservation. Instead, it appears that this disparity is a function of both taphonomic process bias and collection bias towards larger body fossils, greater impact of weathering and montmorillonite soil rotation on smaller fossils, and lack of outcrop. The application of novel techniques to field exploration in Central West Queensland might facilitate the identification of additional tracksites with a preservation mode more akin to that of the Snake Creek Tracksite than that of Lark Quarry, as well as the discovery of more non-sauropod dinosaur and non-crocodyliform body fossils in the ‘upper’ Winton Formation. This might shed light on the identity of the tridactyl trackmakers at Lark Quarry and the Snake Creek tracksites, heretofore barely represented by body fossils from the Winton Formation.

## Institutional abbreviations

AAOD: Australian Age of Dinosaurs Museum of Natural History, Winton, Queensland
AODF: Australian Age of Dinosaurs Fossil
AODL: Australian Age of Dinosaurs Locality
QM F: Queensland Museum Fossil, Brisbane, Australia

## Additional Information and Declarations

### Data Availability

The following information was supplied regarding data availability: Snake Creek Tracksite: Sauropod, theropod (*Skartopus australis*), ornithopod (*Wintonopus latomorum*), crocodyliform (*Hatcherichnus* isp.), and ?turtle tracks, and ?lungfish and ?actinopterygian feeding traces: DOI 10.17602/M2/M355391. *Skartopus australis* trackway AODF 904.T1, ?*Wintonopus latomorum* possible trackway AODF 904.O1, and ?*Hatcherichnus* possible trackway AODF 904.C4: DOI 10.17602/M2/M355396. *Hatcherichnus* trackway AODF 904.C1, with one right track from *Hatcherichnus* trackway AODF 904.C5: DOI 10.17602/M2/M355400. *Hatcherichnus* trackway AODF 904.C2: DOI 10.17602/M2/M355404.
